# *p*-Nitrophenyl carbonate promoted ring-opening reactions of DBU and DBN affording lactam carbamates

**DOI:** 10.3762/bjoc.12.197

**Published:** 2016-09-26

**Authors:** Madhuri Vangala, Ganesh P Shinde

**Affiliations:** 1 Department of Chemistry, Indian Institute of Science Education and Research, Pune 411 008, India

**Keywords:** carbonates, DBN, DBU, lactams, *p*-nitrophenyl

## Abstract

The amidine bases DBU (1,8-diazabicyclo[5.4.0]undec-7-ene) and DBN (1,5-diazabicyclo[4.3.0]non-5-ene) display nucleophilic behaviour towards highly electrophilic *p*-nitrophenyl carbonate derivatives with ring opening of the bicyclic ring to form corresponding substituted ε-caprolactam and γ-lactam derived carbamates. This simple method presents a unified strategy to synthesize structurally diverse ε-caprolactam and γ-lactam compounds with a large substrate scope.

## Introduction

Among various organic bases, amidines such as DBU (1,8-diazabicyclo[5.4.0]undec-7-ene) and DBN (1,5-diazabicyclo[4.3.0]non-5-ene) having an imino group attached to the α-carbon of the amine are synthetically useful and strong neutral bases. Besides, DBU and DBN catalyze various organic reactions such as dehydrohalogenations [1], carbonylations [2], amidations [3] and Baylis–Hillman reactions [4]. These bicyclic amidines have been thought to be non-nucleophilic bases, but meanwhile numerous examples unveiled their ability to act as C and N nucleophiles [5–8]. In 1981, McCoy and Mal first isolated an adduct of DBU with dimethyl 1-chloro-3-methyl cyclopropane-1,2-dicarboxylate during the dehydrohalogenation of halocyclopropanes [9]. In 1993, Bertrand and co-workers, showed that DBU and DBN act as nucleophiles towards halo derivatives of main group elements where the DBU and DBN bicyclic rings remained unaffected [10–11]. Later in 1994, Lammers et al. observed the nucleophilicity of amidine bases with 4-halo-3,5-dimethyl-1-nitro-1*H*-pyrazole and their subsequent ring opening leading to the lactam products [12]. Subsequently, Ma and Dolphin isolated chlorin-*e*_6_ lactams from the reaction of methyl pheophorbide with DBU and DBN promoted by trialkyl triflates [13]. Additionally, the conjugate addition reaction of DBU to diarylpyrone [14] and Baylis–Hillman acetates [15] also gave caprolactam products. A closer look at these results suggested that the nucleophilic behavior of DBU highly depends on specific substrates. Vaidyanathan and co-workers reported the DBU-catalyzed addition of amines to acyl imidazoles [16], however, using a stoichiometric amount of DBU, Rajagopal et al. observed nucleophile behavior of DBU towards imidazolides providing ε-caprolactam-derived carbamates and amides [17]. Here, in this report we present the results obtained by the reaction of DBU and DBN with highly electrophilic *p*-nitrophenyl carbonates leading to ε-caprolactam and γ-lactam carbamates.

*p*-Nitrophenyl carbonates are highly reactive compounds that are usually treated with alcohols or amines to give either a new carbonate or a carbamate-linked compound depending on the nucleophile. In one of our earlier reports, polycarbamate nucleic acids were synthesized from *p*-nitrophenyl carbonates with amines of nucleic acid derivatives [18]. Very recently, Hotha et al. utilized 1-ethynylcyclohexyl *p*-nitrophenyl carbonate to synthesize alkynyl glycosyl carbonate donors from hemiacetals [19]. Also, glycocarbamates [20] obtained from glycosyl *p*-nitrophenyl carbonates [21–24], were explored in studies of carbohydrate–protein interactions [25], ligation and surfactant properties [26–27]. Although *p*-nitrophenyl carbonates were extensively utilized in these reactions, the nucleophilicity of amidine bases towards these carbonates was not encountered so far. In continuation of our interest in carbohydrates [28–29] and the synthesis of carbamate-linked compounds using *p*-nitrophenyl carbonates, we herein report our results from nucleophilic ring opening reactions of DBU and DBN using *p*-nitrophenyl carbonates.

## Results and Discussion

**Scheme 1:**
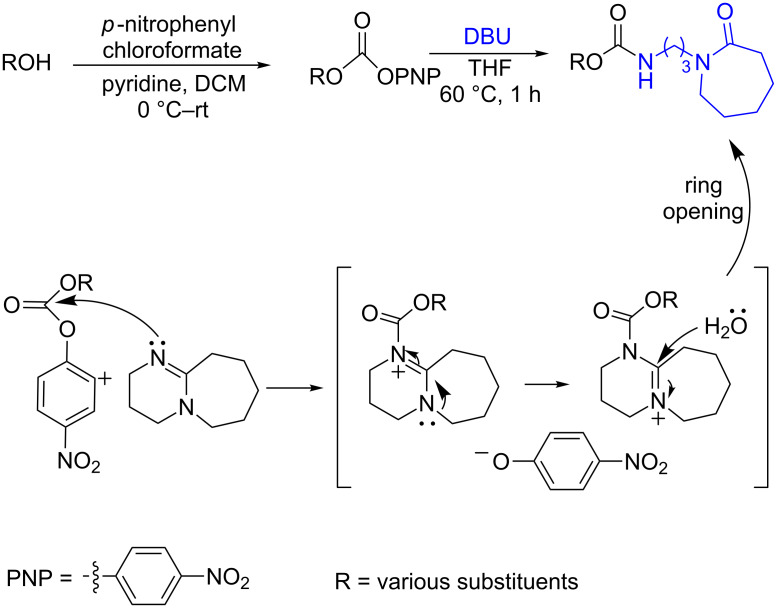
Reaction of DBU with *p*-nitrophenyl carbonate.

In the course of this study, we observed that in the absence of a nucleophile the *p*-nitrophenyl carbonate of 1-ethynylcyclohexanol (Table 1, **1a**) in THF, exclusively afforded the ε-caprolactam product **1b** when 2 equiv of DBU were used. After 1 h reaction at room temperature almost all starting material was consumed (90%, TLC) and heating the reaction mixture to 60 °C led to complete consumption of the starting material within 1 h to give product **1b**. The transformation involves a nucleophilic attack of the imine nitrogen onto the carbonyl carbon followed by the elimination of a *p*-nitrophenoxide ion. Subsequently, the imine carbon of DBU is attacked by water molecules present in the solvent, leading to the ring opening and formation of the corresponding caprolactam carbamates (Scheme 1).

**Scheme 2:**
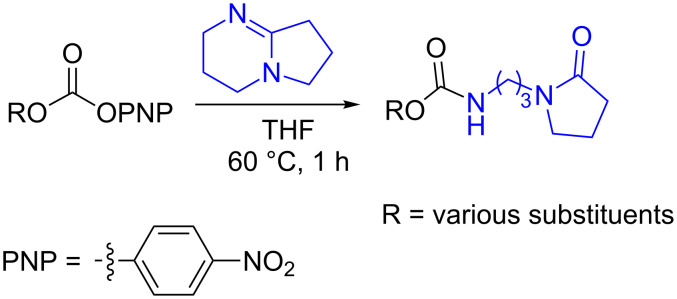
Reaction of DBN with *p*-nitrophenyl carbonates.

The structure of **1b** was confirmed by ^1^H and ^13^C spectroscopy, which showed the characteristic carbamate NH triplet at 5.80 ppm in the ^1^H NMR spectrum and the expected peaks at 176.6 and 155.0 ppm in the ^13^C NMR spectrum for the caprolactam and carbamate carbonyl carbons, respectively. Additionally, HRMS and IR absorptions of the carbonyl groups at 1713, 1624 and carbamate NH at 3301 cm^−1^ also confirmed the ring opening of DBU. Excited with the outcome of the reaction, we set out to explore the structural diversity using different *p*-nitrophenyl carbonates which were prepared by treating an alcohol with *p*-nitrophenyl chloroformate in CH_2_Cl_2_ using pyridine as a base. Thus, homopropargyl alcohol, decanol, cholesterol and *N*-Boc-*trans*-4-hydroxy-L-proline methyl ester gave the corresponding carbonates (Table 1, **2a–5a**) in good to excellent yields. Subsequently, the purified carbonates dissolved in THF were treated with 2 equiv of DBU. Heating the reaction mixture to 60 °C for 1 h gave the caprolactam carbamate products **2b–5b** along with *p*-nitrophenol as byproduct. In case of the ε-caprolactam of *N*-Boc-*trans*-4-hydroxy-L-proline methyl ester **5b**, rotamers due to flipping of the *N*-Boc group were obtained. Owing to the importance of sugar caprolactams in polymerizations, 2,3-di-*O*-benzyl-4-*O*-*p*-methoxybenzyl-α-methyl-D-glucopyranoside and 2,3,4-tri-*O*-benzoyl-α-methyl-D-glucopyranoside [30–31] were converted into the *p*-nitrophenyl carbonates **6a** and **7a** in good yields. The corresponding carbonate of per-*O*-benzoyl glucopyranose **8a** [30–31] was obtained in only moderate yield and as a mixture of α and β anomers which was used without further purification. The carbonates were subsequently reacted with DBU under the same conditions as described above, giving **6b** and **7b** in 82% and 87% yield, and **8b** as a mixture of α and β anomers in 48% yield. Encouraged by these results, we turned to evaluate the nucleophilicity of DBN towards *p*-nitrophenyl carbonate derivatives (Scheme 2).

**Table 1:** Synthesis of ε-caprolactam-derived carbamates **1b–8b**.

No	carbonate	product	yield

1	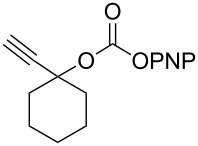 **1a**	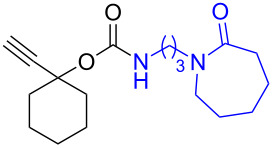 **1b**	85%
2	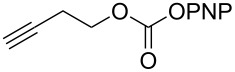 **2a**	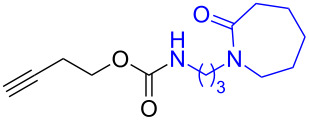 **2b**	79%
3	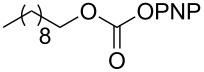 **3a**	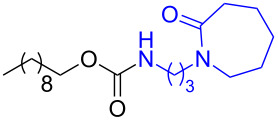 **3b**	88%
4	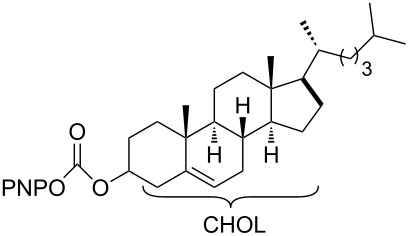 **4a**	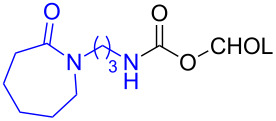 **4b**	69%
5	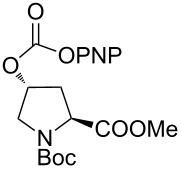 **5a**	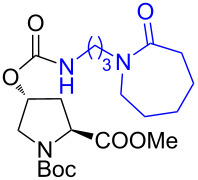 **5b**	71%
6	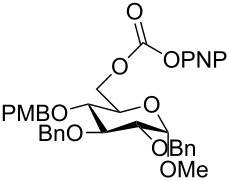 **6a**	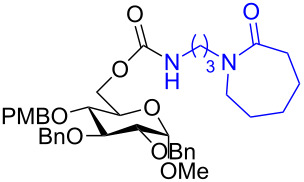 **6b**	82%
7	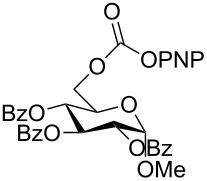 **7a**	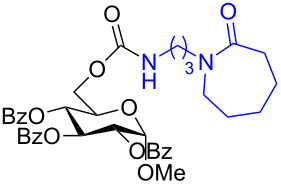 **7b**	87%
8	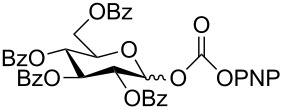 **8a**	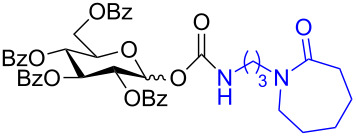 **8b**	48%

Next, the reaction of the *p*-nitrophenyl carbonate of homopropargyl alcohol **2a** in THF with 2 equiv of DBN was examined. Similar to the results observed with DBU, more than 90% of the reaction was complete at room temperature in 1 h. However, heating the reaction mixture to 60 °C for 1 h resulted in completion of the reaction giving the γ-lactam carbamate **2ab** in 56% yield (Table 2). The ^1^H NMR spectrum of the product **2ab** showed a broad singlet at 5.70 ppm assigned to the carbamate NH. The corresponding peaks at 175.6, 156.3 ppm in the ^13^C NMR spectrum as well as the HRMS and IR data confirmed the ring opening of DBN. To evaluate the substrate feasibility, one phenol, an allylic alcohol and three sugar alcohols were subjected to the reaction. The 3,4-dimethylphenyl *p*-nitrophenyl carbonate (**9a**) and geranyl carbonate **10a** gave the corresponding γ-lactams **9b** and **10b** in 62% and 46% yields, respectively. Similarly, the *p*-nitrophenyl carbonate of D-psicofuranose [28], *n*-pentenyl 2,3,4-tri-*O*-benzyl-α-D-mannopyranoside [32–34] and 2,3-di-*O*-benzyl-α-methyl-D-arabinofuranoside [35] (Table 2, **11a–13a)** gave the γ-lactam-derived carbamates **11b–13b** in 53%, 63% and 67% yield, respectively.

**Table 2:** Synthesis of γ-lactam-derived carbamates **2ab**, **9b–16b**.

No	carbonate	product	yield

1	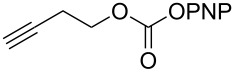 **2a**	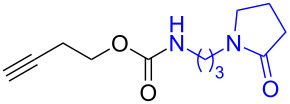 **2ab**	56%
2	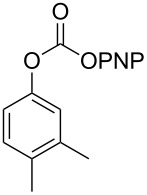 **9a**	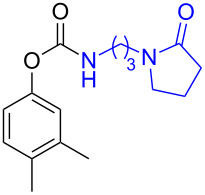 **9b**	62%
3	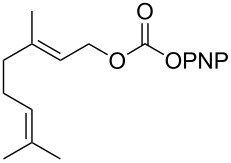 **10a**	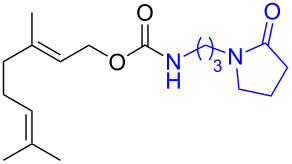 **10b**	46%
4	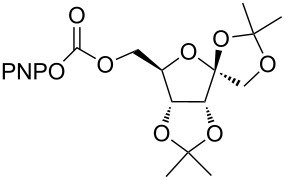 **11a**	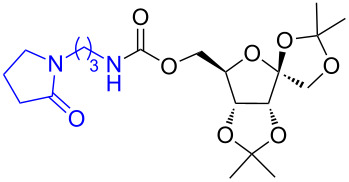 **11b**	53%
5	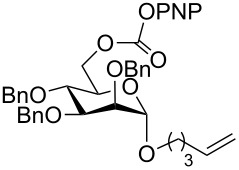 **12a**	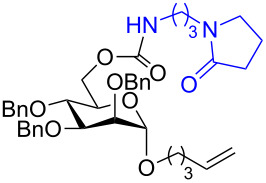 **12b**	63%
6	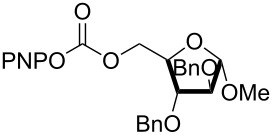 **13a**	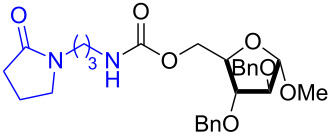 **13b**	67%
7	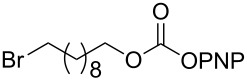 **14a**	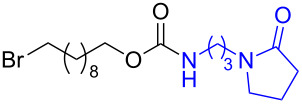 **14b** 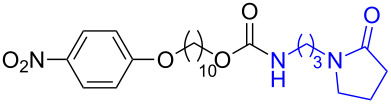 **14c**	64%
8	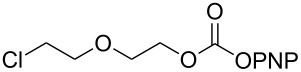 **15a**	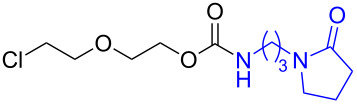 **15b**	41%^a^ 30%^b^
9	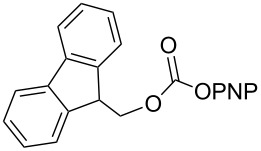 **16a**	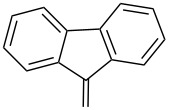 **16b**	
10	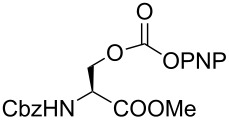 **17a**	mixture of products	

^a^reaction at 60 °C and ^b^at rt.

As DBU and DBN are known to promote dehydrohalogenation reactions, we turned our attention to halogenated alcohols. Thus, the *p*-nitrophenyl carbonate of 10-bromo-1-decanol **14a** was reacted with DBN at 60 °C for 1 h affording a single polar spot on TLC. To our surprise, the ^1^H NMR spectrum showed the existence of two compounds with the carbamate NH showing a multiplet rather than a triplet and an additional signal for the *p*-nitrophenyl group. In the ^13^C NMR spectrum, peaks at 175.7 and 157.0 ppm for the cyclic amide and carbamate carbon confirmed the ring opening of DBN. However, the appearance of new peaks in the ^13^C NMR at 164.3, 141.2 and 126.0 ppm and the upfield shift of C2 of *p*-nitrophenyl substituent from 122 ppm to 114.4 ppm, suggested that DBN displaced a bromide with the *p*-nitrophenoxide ion to give compound **14c**. Thus, DBN played a dual role in the reaction with **14a** – namely as nucleophile and as base giving products **14b** and **14c** in a 1:1 ratio. The peaks at 164.3 and 114.4 ppm can therefore be assigned to the ipso carbon and ortho carbon of the *p*-nitrophenyl substituent in **14c**. This observation was particularly interesting as N-alkylation of DBN [36] was not favored and instead underwent substitution. To test if the reaction favors both – nucleophilic addition as well as substitution – at lower temperature, the reaction was performed at room temperature for 1.5 h. Although both products were observed the substitution product was minor product, as seen by the integration of *p*-nitrophenol peaks in the ^1^H NMR. On the contrary, the reaction of the 2-(2-chloroethoxy)ethanol carbonate **15a** with DBN at room temperature and at 60 °C gave only the ring-opened product of DBN **15b** in 30% and 41% yield, due to the poor leaving ability of chloride relative to bromide. Further, addition of DBN to the acidic-proton containing substrates such as the *p*-nitrophenyl carbonate of 9-fluorenemethanol **16a** and *N*-Cbz-L-serine methyl ester **17a**, resulted in the dibenzofulvene product **16b** in the former, and a mixture of products in the latter, with DBN acting as a base. Even though the γ-lactam products were the only major compounds noticed, the yields were substantially lower than the caprolactam products, presumably due to the ease of ring opening of DBU.

To check if the nucleophilicity of DBU/DBN was specific to the highly electron deficient *p*-nitrophenyl carbonate, a set of three different carbonates of 1-ethynylcyclohexanol were synthesized using phenyl, benzyl and ethyl chloroformate, respectively. The reaction of the phenyl carbonate (Table 3, **A**) with DBU at 60 °C for 20 h gave ε-caprolactam (**1b**) with 12% yield. In contrast, in the reaction of benzyl and ethyl carbonates (**B**, **C**) with DBU no trace of lactam (**1b**) was formed and the substrates remained unaffected due to the poor leaving nature of the alkoxides in comparison to phenolates. This suggests that a highly electrophilic center is the prerequisite for the nucleophilic behavior of DBU and DBN to come into play.

**Table 3:** Reactivity of different carbonates with DBU.

	R = PhNO_2_ (**1a**)	R = Ph (**A**)	R = CH_2_Ph (**B**)	R = C_2_H_5_ (**C**)
	
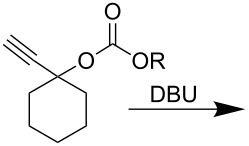	Product **1b** 85% (60 °C, 1 h)	Product **1b** 12% (60 °C, 20 h)	**no rxn** (60 °C, 20 h)	**no rxn** (60 °C, 20 h)

Finally, nearly quantitative large scale transformations were achieved, when 3.5 g of substrates **3a** and **7a** were reacted with DBU at 60 °C for 1 h giving lactams **3b** and **7b** in 90% and 94% yield, respectively.

## Conclusion

In conclusion, we have shown an operationally simple synthesis of carbamate-derived ε-caprolactam and γ-lactam compounds utilizing the nucleophilicity of DBU/DBN and highly electrophile *p*-nitrophenyl carbonate derivatives. The reactions proceeded even at room temperature and displayed the nucleophilic addition and substitution with the *p*-nitrophenyl carbonate derivative of 10-bromodecanol. These caprolactam derivatives may find application in polymer chemistry.

## Supporting Information

File 1 Detailed experimental procedures, compound characterization and copies of 1H and 13C NMR spectra of all new compounds.
