# Supplementary material for: p-Nitrophenyl carbonate promoted ring-opening reactions of DBU and DBN affording lactam carbamates
Source: Beilstein J Org Chem. 2016 Sep 26;12:2086–92. doi: 10.3762/bjoc.12.197 (PMC5082618; doi:10.3762/bjoc.12.197)
Supplement: File 1 — Detailed experimental procedures, compound characterization and copies of 1H and 13C NMR spectra of all new compounds. [file Beilstein_J_Org_Chem-12-2086-s001.pdf]

Supporting Information

for

p-Nitrophenyl carbonate promoted ring-opening reactions of DBU and DBN affording lactam carbamates

Madhuri Vangala* and Ganesh P Shinde

Address: Department of Chemistry, Indian Institute of Science Education and Research, Pune 411 008, India

Email: Madhuri Vangala* - madhuri@iiserpune.ac.in

* Corresponding author

Contents **Page**

Experimental section and general procedures S2

Experimental data of **2a**–**7a**, **9a**–**17a** S2

Experimental data of **1b**–**7b**, **2ab**, **9b**–**15b** and compounds **A**, **B**, **C** S4

^{1}H, ^{13}C NMR Spectra of compounds **1a**–**7a**, **9a**–**17a** S8

^{1}H, ^{13}C NMR Spectra of compounds **1b**–**15b**, **2ab** S23

Experimental section

General Methods. All chemicals and reagents were purchased from commercial sources and used without purification. ^{1}H NMR and ^{13}C NMR were recorded in $CDCl_3$ using TMS as internal standard on 400 and 100 MHz spectrometer, respectively. Coupling constants (J) are reported in hertz (Hz) and signal multiplicity is abbreviated as s = singlet, d = doublet, dd = doublet of doublet, dt = doublet of triplet, td = triplet of doublet, t = triplet, q = quartet, p = pentet, m = multiplet, br = broad. IR spectra were recorded on a FT-IR spectrometer, and only major peaks were reported in cm^{-1}. Optical rotations were measured on a polarimeter using sodium light (D line 589 nm). High resolution mass spectra (HRMS) were obtained by ESI (TOF) ionization. Column chromatography was performed over silica gel (100–200 mesh). Melting points were determined in capillary tubes on an electrically heated block and are uncorrected.

General procedure for the synthesis of *p*-nitrophenyl carbonate derivatives

The alcohol (0.5 g) was dissolved in dry DCM (6 mL) and cooled to 0 °C in an ice bath. Dry pyridine (2 equiv) was added and the mixture was allowed to stir for 10 min. *p*-Nitrophenyl chloroformate (1.2 equiv) dissolved in DCM (2 mL) was added stirring continued for 1.5 h at room temperature. After completion, the reaction mixture was extracted with DCM (2 × 30 mL), the organic layer washed with water, brine and dried over anhydrous Na_2SO_4. The solvent was evaporated and the crude compound was purified by column chromatography using hexane/ethylacetate.

General procedure for the synthesis of ε-caprolactam and γ-lactam derived compounds

The *p*-nitrophenyl carbonate derivative (0.3 g) was dissolved in AR grade THF (5 mL) at room temperature and DBU or DBN (2 equiv) was added. The temperature of the reaction mixture was raised to 60 °C and stirring was continued for 1 h. After completion, the reaction mixture was extracted with ethyl acetate (2 × 30 mL), the organic layer washed with saturated $NaHCO_3$ solution, water, brine and dried over anhydrous Na_2SO_4. The solvent was evaporated and the crude compound was purified by column chromatography.

General procedure for the synthesis of carbonates A, B, C

A. To an ice-cooled solution of 1-ethynylcyclohexanol (1 g) in anhydrous DCM (15 mL) was added pyridine (2 equiv) and stirred for 10 min. Phenyl chloroformate (1.5 equiv) was added, and the reaction mixture was allowed to warm to rt and stirred overnight. The reaction mixture was extracted with DCM (2 × 25 mL), the organic layer washed with saturated $NaHCO_3$ solution, brine and dried over anhydrous Na_2SO_4. The concentrated crude mixture was purified by silica gel column chromatography (*n*-hexane/EtOAc) to afford compound **A**.

B. To an ice-cooled solution of 1-ethynylcyclohexanol (1 g) in anhydrous DMF (15 mL) was added NaH (2 equiv) in portions and stirred for 10 min. Subsequently, benzyl chloroformate or ethyl chloroformate (1.5 equiv) was added. The reaction mixture was allowed to warm to rt and stirred overnight. The reaction mixture was extracted with CH_2Cl_2 (2 × 25 mL), the organic layer washed with saturated $NaHCO_3$ solution, brine and dried over anhydrous Na_2SO_4. The concentrated crude mixture was purified by silica gel column chromatography (*n*-hexane/EtOAc) to afford compound **B** or **C**.

Structural data of *p*-nitrophenyl carbonates 1a–7a, 9a–17a

Compound **1a.** ^{1}H NMR (400 MHz, $CDCl_3$) δ 8.30 – 8.26 (m, 2H), 7.44 – 7.40 (m, 2H), 2.72 (s, 1H), 2.34-2.17 (m, 2H), 2.04-1.91 (m, 2H), 1.80-1.64 (m, 4H), 1.63-1.54 (m, 1H), 1.41-1.32 (m, 1H); ^{13}C NMR (100 MHz, $CDCl_3$) δ 155.5, 150.0, 145.2, 125.2, 121.8, 82.0, 79.7, 75.9, 36.8, 25.0, 22.6. (Data consistent with the literature reference 19).

Compound **2a.** R_f = 0.5 (hexane/EtOAc, 80:20); white solid (86% yield), mp 73-74 °C; IR ($CHCl_3$) ν 3290, 1774, 1589, 1493, 1272, 1216, 773 cm^{-1}; ^{1}H NMR (400 MHz, $CDCl_3$) δ 8.34 – 8.22 (m, 2H), 7.45 – 7.35 (m, 2H), 4.39 (t, J = 6.8 Hz, 2H), 2.70 – 2.65 (m, 2H), 2.08 (t, J = 2.7 Hz, 1H); ^{13}C NMR (100 MHz, $CDCl_3$) δ 155.5, 152.4, 145.6, 125.5, 121.9, 79.1, 70.8, 66.8, 19.1; HRMS (ESI): m/z $[M + Na]^+$ calcd for $C_{11}H_9NNaO_5$:258.0378, Found: 258.0372.

Compound **3a.** R_f = 0.7 (hexane/EtOAc, 80:20); thick liquid (90% yield); IR ($CHCl_3$) ν 1768, 1528, 1349, 1218 cm^{-1}; ^1H NMR (400 MHz, $CDCl_3$) δ 8.28 – 8.22 (m, 2H), 7.40 – 7.34 (m, 2H), 4.27 (t, *J* = 6.7 Hz, 2H), 1.78 – 1.70 (m, 2H), 1.45 – 1.23 (m, 14H), 0.86 (t, *J* = 6.8 Hz, 3H); ^{13}C NMR (100 MHz, $CDCl_3$) δ 155.7, 152.6, 145.4, 125.3, 121.8, 69.7, 32.0, 29.6, 29.5, 29.4, 29.2, 28.6, 25.7, 22.7, 14.2; HRMS (ESI): *m/z* $[M + H]^+$ calcd for $C_{17}H_{26}NO_5$:324.1811, Found: 324.1822.

Compound **4a.** R_f = 0.5 (hexane/EtOAc, 85:15); white solid (80% yield), mp 160-162 °C; $[\alpha]_D^{25}$ = -28.42 (c 0.42, CH_2Cl_2); IR ($CHCl_3$) *v* 1760, 1521, 1350, 1257 cm^{-1}; ^1H NMR (400 MHz, $CDCl_3$) δ 8.24 (dd, *J* = 7.4, 1.8 Hz, 2H), 7.36 (d, *J* = 9.1 Hz, 2H), 5.40 (d, *J* = 3.7 Hz, 1H), 4.66 – 4.50 (m, 1H), 2.51 – 2.38 (m, 2H), 2.05 – 1.87 (m, 4H), 1.85 – 1.65 (m, 2H), 1.60 – 1.41 (m, 6H), 1.39 – 1.21 (m, 5H), 1.20 – 1.05 (m, 7H), 1.02 (s, 3H), 0.99 – 0.92 (m, 2H), 0.90 (d, *J* = 6.5 Hz, 3H), 0.84 (dd, *J* = 6.6, 1.4 Hz, 6H), 0.66 (s, 3H); ^{13}C NMR (100 MHz, $CDCl_3$) δ 155.7, 151.8, 145.3, 138.9, 125.3, 123.6, 121.8, 79.8, 56.7, 56.2, 50.0, 42.4, 39.8, 39.6, 37.9, 36.9, 36.6, 36.3, 35.9, 32.0, 31.9, 28.3, 28.1, 27.6, 24.4, 23.9, 22.9, 22.7, 21.1, 19.3, 18.8, 11.9; HRMS (ESI): *m/z* $[M + Na]^+$ calcd for $C_{34}H_{49}NNaO_5$:574.3508, Found: 574.3512.

Compound **5a.** R_f = 0.4 (hexane/EtOAc, 75:25); thick liquid (79% yield); $[\alpha]_D^{25}$ = -24.62 (c 0.46, CH_2Cl_2); IR ($CHCl_3$) *v* 1757, 1698, 1526, 1201, 758 cm^{-1}; ^1H NMR (400 MHz, $CDCl_3$) δ 8.32 – 8.25 (m, 2H), 7.41 – 7.34 (m, 2H), 5.36 – 5.28 (m, 1H), 4.53 – 4.39 (m, 1H), 3.91 – 3.71 (m, 2H), 3.75 (s, 3H), 2.64 – 2.50 (m, 1H), 2.35 – 2.23 (m, 1H), 1.43 (d, 9H); ^{13}C NMR (100 MHz, $CDCl_3$) δ 172.9, 172.7, 155.3, 154.1, 153.6, 151.9, 145.7, 125.5, 121.8, 81.0, 78.0, 77.3, 57.8, 57.4, 52.6, 52.4, 52.1, 52.0, 36.5, 35.6, 28.5, 28.3; HRMS (ESI): *m/z* $[M + Na]^+$ calcd for $C_{18}H_{22}N_2NaO_9$:433.1222, Found: 433.1227.

Compound **6a.** R_f = 0.4 (hexane/EtOAc, 75:25); white solid (88% yield), mp 94-95 °C; $[\alpha]_D^{25}$ = +64.80 (c 0.38, CH_2Cl_2); IR ($CHCl_3$) *v* 1768, 1609, 1520, 1211, 1036, 740; ^1H NMR (400 MHz, $CDCl_3$) δ 8.31 – 8.24 (m, 2H), 7.44 – 7.30 (m, 12H), 7.25 – 7.19 (m, 2H), 6.89 – 6.83 (m, 2H), 5.04 (d, *J* = 10.8 Hz, 1H), 4.91 – 4.79 (m, 3H), 4.68 (d, *J* = 12.1 Hz, 1H), 4.62 (d, *J* = 3.6 Hz, 1H), 4.57 (d, *J* = 10.8 Hz, 1H), 4.43 (dd, *J* = 11.4, 2.4 Hz, 1H), 4.37 (dd, *J* = 11.5, 4.4 Hz, 1H), 4.04 (t, *J* = 9.2 Hz, 1H), 3.90 – 3.83 (m, 1H), 3.75 (s, 3H), 3.61 – 3.49 (m, 2H), 3.40 (s, 3H). ^{13}C NMR (100 MHz, $CDCl_3$) δ 159.5, 155.5, 152.3, 145.4, 138.7, 138.0, 129.9, 128.6, 128.6, 128.2, 128.1, 128.0, 127.8, 125.3, 121.7, 114.0, 113.8, 98.2, 82.1, 79.8, 76.4, 75.8, 74.7, 73.5, 68.4, 67.7, 55.5, 55.3; HRMS (ESI): *m/z* $[M + Na]^+$ calcd for $C_{36}H_{37}NNaO_{11}$:682.2264, Found: 682.2271.

Compound **7a.** R_f = 0.5 (hexane/EtOAc, 80:20); thick liquid (89% yield); $[\alpha]_D^{25}$ = +76.05 (c 0.65, CH_2Cl_2); IR ($CHCl_3$) *v* 1771, 1725, 1526, 1252, 1097, 701 cm^{-1}; ^1H NMR (400 MHz, $CDCl_3$) δ 8.28 – 8.22 (m, 2H), 8.01 – 7.93 (m, 4H), 7.90 – 7.85 (m, 2H), 7.53 – 7.45 (m, 2H), 7.43 – 7.33 (m, 7H), 7.29 – 7.23 (m, 2H), 6.23 (t, *J* = 9.8 Hz, 1H), 5.69 (t, *J* = 9.9 Hz, 1H), 5.33 (dt, *J* = 9.9, 3.6 Hz, 2H), 4.57 (dd, *J* = 12.0, 4.6 Hz, 1H), 4.49 (dd, *J* = 12.0, 2.5 Hz, 1H), 4.38 (ddd, *J* = 10.2, 4.4, 2.6 Hz, 1H), 3.50 (s, 3H); ^{13}C NMR (100 MHz, $CDCl_3$) δ 165.9, 165.8, 165.5, 155.5, 152.4, 145.5, 133.7, 133.5, 133.3, 129.9, 129.9, 129.7, 129.1, 128.9, 128.7, 128.5, 128.5, 128.4, 125.3, 121.9, 97.2, 71.9, 70.2, 69.0, 67.4, 66.8, 55.9; HRMS (ESI): *m/z* $[M + Na]^+$ calcd for $C_{35}H_{29}NNaO_{13}$:694.1536, Found: 694.1531.

Compound **9a.** R_f = 0.5 (hexane/EtOAc, 85:15); white solid (82% yield), mp 119-121 °C; IR ($CHCl_3$) ν 1770, 1600, 1523, 1247 cm^{-1}; ^1H NMR (400 MHz, $CDCl_3$) δ 8.34 – 8.27 (m, 2H), 7.50 – 7.45 (m, 2H), 7.17 (d, *J* = 8.2 Hz, 1H), 7.05 (d, *J* = 2.3 Hz, 1H), 7.00 (dd, *J* = 8.2, 2.5 Hz, 1H), 2.29 (s, 3H), 2.27 (s, 3H); ^{13}C NMR (100 MHz, $CDCl_3$) δ 155.5, 151.4, 148.8, 145.7, 138.5, 135.3, 130.7, 125.5, 121.9, 121.7, 117.8, 20.0, 19.3; HRMS (ESI): *m/z* $[M + H]^+$ calcd for $C_{15}H_{14}NO_5$:288.0872, Found: 288.0901.

Compound **10a.** R_f = 0.6 (hexane/EtOAc, 85:15); thick liquid (77% yield); IR ($CHCl_3$) ν 1763, 1525, 1447, 1200 cm^{-1}; ^1H NMR (400 MHz, $CDCl_3$) δ 8.31 – 8.23 (m, 2H), 7.42 – 7.35 (m, 2H), 5.48 – 5.41 (m, 1H), 5.12 – 5.05 (m, 1H), 4.80 (d, *J* = 7.3 Hz, 2H), 2.19 – 2.05 (m, 4H), 1.77 (d, *J* = 0.8 Hz, 3H), 1.68 (s, 3H), 1.61 (s, 3H); ^{13}C NMR (100 MHz, $CDCl_3$) δ 155.8, 152.7, 145.4, 145.0, 132.2, 125.4, 123.6, 121.9, 116.8, 66.2, 39.7, 26.3, 25.8, 17.8, 16.7; HRMS (ESI): *m/z* $[M + H]^+$ calcd for $C_{17}H_{22}NO_5$:320.1498, Found: 320.1502.

Compound **11a.** R_f = 0.5 (hexane/EtOAc, 70:30); thick liquid (77% yield); $[\alpha]_D^{25}$ = -37.27 (c 0.44, CH_2Cl_2); IR ($CHCl_3$) *v* 1770, 1526, 1213, 1069, 858 cm^{-1}; ^1H NMR (400 MHz, $CDCl_3$) δ 8.31 – 8.26 (m, 2H), 7.41 – 7.36 (m, 2H), 4.79 (d, *J* = 5.6 Hz, 1H), 4.66 (d, *J* = 5.8 Hz, 1H), 4.44 – 4.35 (m, 3H), 4.31 (d, *J* = 9.8 Hz, 1H), 4.09 (d, *J* = 9.8 Hz, 1H), 1.46 (s, 3H), 1.45 (s, 3H), 1.38 (s, 3H), 1.34 (s, 3H); ^{13}C NMR (100 MHz, $CDCl_3$) δ 155.6, 152.4, 145.6, 125.5, 121.8, 113.9, 113.2, 112.1, 85.2, 82.4, 82.0, 69.9, 68.9, 26.5, 26.4, 25.2; HRMS (ESI): *m/z* $[M + Na]^+$ calcd for $C_{19}H_{23}NNaO_{10}$:448.1219, Found: 448.1227.

Compound **12a.** R_f = 0.5 (hexane/EtOAc, 70:30); thick liquid (84% yield); $[\alpha]_D^{25}$ = +36.63 (c 1.09, CH_2Cl_2); IR ($CHCl_3$) *ν* 1769, 1525, 1215, 1094, 741 cm^{-1}; ^{1}H NMR (400 MHz, $CDCl_3$) δ 8.25 (d, *J* = 5.9 Hz, 2H), 7.48 – 7.27 (m, 17H), 5.90 – 5.75 (m, 1H), 5.10 – 4.96 (m, 3H), 4.93 – 4.86 (m, 1H), 4.84 – 4.75 (m, 2H), 4.72 – 4.65 (m, 3H), 4.62 – 4.55 (m, 1H), 4.53 – 4.46 (m, 1H), 4.06 – 3.96 (m, 2H), 3.95 – 3.82 (m, 2H), 3.76 – 3.66 (m, 1H), 3.49 – 3.37 (m, 1H), 2.17 – 2.06 (m, 2H), 1.74 – 1.61 (m, 2H); ^{13}C NMR (100 MHz, $CDCl_3$) δ 155.6, 152.4, 145.4, 138.3, 138.2, 138.0, 128.6, 128.5, 128.5, 128.2, 127.9, 127.9, 127.8, 125.3, 121.9, 115.1, 98.1, 80.2, 75.3, 74.7, 74.4, 72.9, 72.2, 69.9, 68.3, 67.3, 30.3, 28.6; HRMS (ESI): *m/z* $[M + Na]^+$ calcd for $C_{39}H_{41}NNaO_{10}$:706.2627, Found: 706.2631.

Compound **13a.** R_f = 0.4 (hexane/EtOAc, 80:20); thick liquid (82% yield); $[\alpha]_D^{25}$ = +55.64 (c 0.61, CH_2Cl_2); IR ($CHCl_3$) *ν* 1768, 1596, 1524, 1213 cm^{-1}; ^{1}H NMR (400 MHz, $CDCl_3$) δ 8.28 – 8.23 (m, 2H), 7.39 – 7.28 (m, 12H), 4.99 (s, 1H), 4.66 – 4.44 (m, 5H), 4.38 – 4.28 (m, 2H), 4.04 (dd, *J* = 2.8, 0.8 Hz, 1H), 3.91 (dd, *J* = 6.2, 2.7 Hz, 1H), 3.42 (s, 3H); ^{13}C NMR (100 MHz, $CDCl_3$) δ 155.5, 152.4, 145.5, 137.5, 137.3, 128.6, 128.2, 128.1, 128.1, 128.0, 125.4, 121.8, 107.6, 87.8, 83.3, 79.0, 72.5, 72.3, 68.3, 55.3; HRMS (ESI): *m/z* $[M + Na]^+$ calcd for $C_{27}H_{27}NNaO_9$:532.1583, Found: 532.1577.

Compound **14a.** R_f = 0.5 (hexane/EtOAc, 85:15); thick liquid (86% yield); IR ($CHCl_3$) ν 1764, 1525, 1252, 858 cm^{-1}; ^{1}H NMR (400 MHz, $CDCl_3$) δ 8.31 – 8.22 (m, 2H), 7.42 – 7.33 (m, 2H), 4.27 (t, *J* = 6.7 Hz, 2H), 3.40 (t, *J* = 6.8 Hz, 2H), 1.89 – 1.80 (m, 2H), 1.79 – 1.70 (m, 2H), 1.46 – 1.37 (m, 4H), 1.30 (s, 8H); ^{13}C NMR (100 MHz, $CDCl_3$) δ 155.7, 152.6, 145.4, 125.4, 121.9, 69.8, 34.1, 32.9, 29.4, 29.2, 28.8, 28.6, 28.2, 25.7; HRMS (ESI): *m/z* $[M + Na]^+$ calcd for $C_{17}H_{24}BrNNaO_5$:424.0736, Found: 424.0737.

Compound **15a.** R_f = 0.5 (hexane/EtOAc, 80:20); thick liquid (83% yield); IR ($CHCl_3$) ν 1765, 1593, 1520, 1339, 1206, 812 cm^{-1}; ^{1}H NMR (400 MHz, $CDCl_3$) δ 8.29 – 8.23 (m, 2H), 7.41 – 7.34 (m, 2H), 4.47 – 4.42 (m, 2H), 3.85 – 3.77 (m, 4H), 3.65 (t, *J* = 5.7 Hz, 2H); ^{13}C NMR (100 MHz, $CDCl_3$) δ 155.6, 152.5, 145.5, 125.4, 121.9, 71.4, 68.7, 68.1, 42.8; HRMS (ESI): *m/z* $[M + H]^+$ calcd for $C_{11}H_{13}ClNO_6$:290.0431, Found: 290.0440.

Compound **16a.** R_f = 0.4 (hexane/EtOAc, 80:20); light yellow solid (81% yield), mp 87-88 °C; IR ($CHCl_3$) ν 1766, 1528, 1453, 1225, 913 cm^{-1}; ^{1}H NMR (400 MHz, $CDCl_3$) δ 8.23 (d, *J* = 9.0 Hz, 2H), 7.81 (d, *J* = 7.5 Hz, 2H), 7.68 (d, *J* = 7.4 Hz, 2H), 7.47 (t, *J* = 7.4 Hz, 2H), 7.39 (t, *J* = 7.3 Hz, 2H), 7.32 (d, *J* = 9.1 Hz, 2H), 4.62 (d, *J* = 7.0 Hz, 2H), 4.34 (t, *J* = 7.0 Hz, 1H); ^{13}C NMR (100 MHz, $CDCl_3$) δ 155.4, 152.3, 145.3, 142.8, 141.3, 128.1, 127.2, 125.2, 125.0, 121.7, 120.2, 70.8, 46.5; HRMS (ESI): *m/z* $[M + Na]^+$ calcd for $C_{21}H_{15}NNaO_5$:384.0848, Found: 384.0842.

Compound **17a.** R_f = 0.4 (hexane/EtOAc, 70:30); white solid (68% yield), mp 87-88 °C; $[\alpha]_D^{25}$ = +16.02 (c 0.26, CH_2Cl_2); IR ($CHCl_3$) *ν* 3368, 1762, 1714, 1520, 1203, 858 cm^{-1}; ^{1}H NMR (400 MHz, $CDCl_3$) δ 8.18 (d, *J* = 9.1 Hz, 2H), 7.36 – 7.24 (m, 7H), 5.91 (d, *J* = 8.0 Hz, 1H), 5.17 – 5.05 (m, 2H), 4.77 – 4.71 (m, 1H), 4.65 – 4.54 (m, 2H), 3.77 (s, 3H); ^{13}C NMR (100 MHz, $CDCl_3$) δ 169.3, 155.8, 155.2, 152.1, 145.4, 135.9, 128.5, 128.2, 128.1, 125.2, 121.6, 68.2, 67.3, 53.1, 53.1; HRMS (ESI): *m/z* $[M + Na]^+$ calcd for $C_{19}H_{18}N_2NaO_9$:441.0909, Found: 441.0909.

Structural data of lactam compounds 1b–7b, 2ab and 9b–15b

Compound **1b.** R_f = 0.5 (EtOAc); thick liquid (85% yield); IR ($CHCl_3$) ν 3301, 1713, 1624, 1497, 1237 cm^{-1}; ^{1}H NMR (400 MHz, $CDCl_3$) δ 5.76 (br s, 1H), 3.41 (t, *J* = 6.3 Hz, 2H), 3.33 – 3.26 (m, 2H), 3.10 (dd, *J* = 12.2, 6.2 Hz, 2H), 2.53 (s, 1H), 2.52 – 2.45 (m, 2H), 2.12 – 2.02 (m, 2H), 1.87 – 1.76 (m, 2H), 1.74 – 1.53 (m, 12H), 1.50 – 1.40 (m, 1H), 1.33 – 1.23 (m, 1H); ^{13}C NMR (100 MHz, $CDCl_3$) δ 176.7, 155.0, 84.7, 74.4, 73.6, 49.6, 45.3, 37.4, 37.2, 37.1, 30.0, 28.6, 27.8, 25.2, 23.5, 22.5; HRMS (ESI): *m/z* $[M + Na]^+$ calcd for $C_{18}H_{28}N_2NaO_3$:343.1997, Found: 343.1996.

Compound **2b.** R_f = 0.2 (EtOAc); thick liquid (79% yield); IR ($CHCl_3$) ν 3294, 1708, 1620, 1525, 1440, 1244 cm^{-1}; ^{1}H NMR (400 MHz, $CDCl_3$) δ 5.92 (s, 1H), 4.05 (t, *J* = 7.0 Hz, 2H), 3.35 (t, *J* = 6.3 Hz, 2H), 3.28 – 3.22 (m, 2H), 3.05 (dd, *J* = 12.2, 6.2 Hz, 2H), 2.48 – 2.38 (m, 4H), 1.92 (t, *J* = 2.6 Hz, 1H), 1.70 – 1.51 (m, 8H); ^{13}C NMR (100 MHz, $CDCl_3$) δ 176.6, 156.2, 80.4, 69.7, 62.2, 49.5, 45.0, 37.2, 37.1, 29.9, 28.5, 27.7, 23.3, 19.3; HRMS (ESI): *m/z* $[M + H]^+$ calcd for $C_{14}H_{23}N_2O_3$:267.1709, Found: 267.1718.

Compound **3b.** R_f = 0.2 (EtOAc); white solid (88% yield), mp 68-70 °C; IR ($CHCl_3$) ν 3322, 1709, 1629, 1520, 1246, 731 cm^{-1}; ^{1}H NMR (400 MHz, $CDCl_3$) δ 5.80 – 5.72 (m, 1H), 3.96 (t, *J* = 6.7 Hz, 2H), 3.39 (t, *J* = 6.3 Hz, 2H), 3.31 – 3.24 (m, 2H), 3.08 (dd, *J* = 12.2, 6.2 Hz, 2H), 2.52 – 2.44 (m, 2H), 1.75 – 1.47 (m, 10H), 1.33 – 1.14 (m, 14H), 0.83 (t, *J* = 6.9 Hz, 3H); ^{13}C NMR (100 MHz, $CDCl_3$) δ 176.6, 157.1, 64.8, 49.6, 45.2, 37.3, 37.2,

31.9, 30.0, 29.6, 29.4, 29.3, 29.1, 28.6, 27.9, 25.9, 23.5, 22.7, 14.1; HRMS (ESI): *m/z* $[M + H]^+$ calcd for $C_{20}H_{39}N_2O_3$:355.296, Found: 355.2967.

Compound **4b.** R_f = 0.3 (EtOAc); white solid (69% yield), mp 162-164 °C; $[\alpha]_D^{25}$ = -16.14 (c 0.52, CH_2Cl_2); IR ($CHCl_3$) *ν* 3355, 1708, 1629, 1512, 1247, 733 cm^{-1}; ^1H NMR (400 MHz, $CDCl_3$) δ 5.74 (t, *J* = 6.1 Hz, 1H), 5.29 (d, *J* = 4.9 Hz, 1H), 4.46 – 4.34 (m, 1H), 3.38 (t, *J* = 6.2 Hz, 2H), 3.30 – 3.24 (m, 2H), 3.07 (dd, *J* = 12.0, 6.1 Hz, 2H), 2.49 – 2.44 (m, 2H), 2.32 – 2.16 (m, 2H), 1.91 (ddd, *J* = 12.0, 9.9, 2.7 Hz, 2H), 1.77 (dd, *J* = 9.8, 3.7 Hz, 3H), 1.63 (ddd, *J* = 18.5, 11.0, 5.4 Hz, 8H), 1.52 – 1.35 (m, 7H), 1.25 (ddd, *J* = 23.8, 18.3, 11.0 Hz, 4H), 1.05 (ddd, *J* = 24.7, 12.1, 6.6 Hz, 7H), 0.94 (s, 3H), 0.97 – 0.88 (m, 3H), 0.85 (d, *J* = 6.5 Hz, 3H), 0.80 (dd, *J* = 6.7, 1.7 Hz, 6H), 0.61 (s, 3H); ^{13}C NMR (100 MHz, $CDCl_3$) δ 176.5, 156.3, 140.0, 122.2, 73.9, 56.7, 56.1, 50.0, 49.5, 45.1, 42.3, 39.7, 39.5, 38.6, 37.2, 37.0, 36.5, 36.2, 35.8, 31.9, 31.8, 30.0, 28.6, 28.2, 28.1, 28.0, 27.8, 24.3, 23.8, 23.4, 22.8, 22.6, 21.0, 19.3, 18.7, 11.8; HRMS (ESI): *m/z* $[M + Na]^+$ calcd for $C_{37}H_{62}N_2NaO_3$:605.4658, Found: 605.4670.

Compound **5b.** R_f = 0.2 (EtOAc); thick liquid (71% yield); $[\alpha]_D^{25}$ = -30.87 (c 0.61, CH_2Cl_2); IR ($CHCl_3$) ν 3328, 1699, 1626, 1400, 1158, 1074 cm^{-1}; ^1H NMR (400 MHz, $CDCl_3$) δ 6.02 – 5.94 (m, 1H), 5.10 (br s, 1H), 4.30 – 4.18 (m, 1H), 3.62 (s, 3H), 3.60 – 3.40 (m, 2H), 3.38 – 3.29 (m, 2H), 3.27 – 3.19 (m, 2H), 3.08 – 2.95 (m, 2H), 2.47 – 2.38 (m, 2H), 2.33 – 2.21 (m, 1H), 2.09 – 1.97 (m, 1H), 1.69 – 1.48 (m, 8H), 1.33 (d, *J* = 14.9 Hz, 9H rotamer); ^{13}C NMR (100 MHz, $CDCl_3$) δ (rotamers) 176.7, 173.2, 172.9, 155.7, 155.5, 154.1, 153.4, 80.2, 72.6, 71.9, 57.9, 57.5, 52.4, 52.3, 52.1, 51.9, 49.5, 44.9, 37.0, 36.7, 35.7, 29.8, 28.4, 28.3, 28.1, 27.6, 27.6, 23.3; HRMS (ESI): *m/z* $[M + Na]^+$ calcd for $C_{21}H_{35}N_3NaO_7$:464.2372, Found: 464.2375.

Compound **6b.** R_f = 0.2 (EtOAc); thick liquid (82% yield); $[\alpha]_D^{25}$ = +37.80 (c 0.29, CH_2Cl_2); IR ($CHCl_3$) ν 3351, 1717, 1625, 1513, 1067, 732 cm^{-1}; ^1H NMR (400 MHz, $CDCl_3$) δ 7.40 – 7.27 (m, 10H), 7.20 (d, *J* = 8.6 Hz, 2H), 6.83 (d, *J* = 8.6 Hz, 2H), 5.79 (t, *J* = 6.1 Hz, 1H), 4.99 (d, *J* = 11.0 Hz, 1H), 4.86 – 4.74 (m, 3H), 4.65 (d, *J* = 12.1 Hz, 1H), 4.58 (d, *J* = 3.5 Hz, 1H), 4.50 (d, *J* = 10.3 Hz, 1H), 4.31 (dd, *J* = 11.7, 4.4 Hz, 1H), 4.24 (dd, *J* = 11.6, 1.6 Hz, 1H), 3.97 (t, *J* = 9.3 Hz, 1H), 3.77 (s, 3H), 3.76 – 3.72 (m, 1H), 3.55 – 3.51 (m, 1H), 3.47 (t, *J* = 9.5 Hz, 1H), 3.43 – 3.37 (m, 2H), 3.35 (s, 3H), 3.32 – 3.27 (m, 2H), 3.17 – 3.08 (m, 2H), 2.54 – 2.47 (m, 2H), 1.75 – 1.58 (m, 8H); ^{13}C NMR (100 MHz, $CDCl_3$) δ 176.7, 159.4, 156.4, 138.9, 138.2, 130.3, 129.9, 128.5, 128.4, 128.2, 128.0, 127.9, 127.6, 113.9, 98.1, 82.1, 79.9, 75.7, 74.9, 73.5, 69.1, 63.3, 55.4, 55.3, 49.6, 45.1, 37.5, 37.2, 30.0, 28.6, 28.0, 23.5; HRMS (ESI): *m/z* $[M + H]^+$ calcd for $C_{39}H_{51}N_2O_9$:691.3594, Found: 691.3602.

Compound **7b.** R_f = 0.2 (EtOAc); white solid (87% yield), mp 70-72 °C; $[\alpha]_D^{25}$ = +61.72 (c 0.32, CH_2Cl_2); IR ($CHCl_3$) *ν* 3397, 1722, 1628, 1447, 1250, 1096, 704 cm^{-1}; ^1H NMR (400 MHz, $CDCl_3$) δ 8.00 – 7.90 (m, 4H), 7.89 – 7.84 (m, 2H), 7.53 – 7.46 (m, 2H), 7.44 – 7.33 (m, 5H), 7.31 – 7.25 (m, 2H), 6.13 (t, *J* = 9.7 Hz, 1H), 5.82 (t, *J* = 6.2 Hz, 1H), 5.60 – 5.51 (m, 1H), 5.25 (dt, *J* = 10.6, 3.6 Hz, 2H), 4.31 – 4.22 (m, 3H), 3.47 (s, 3H), 3.41 (dd, *J* = 10.9, 6.2 Hz, 2H), 3.34 – 3.28 (m, 2H), 3.15 – 3.08 (m, 2H), 2.54 – 2.50 (m, 2H), 1.75 – 1.58 (m, 8H); ^{13}C NMR (100 MHz, $CDCl_3$) δ 176.7, 165.9, 165.9, 165.3, 156.2, 133.4, 133.2, 130.0, 130.0, 129.8, 129.3, 129.2, 129.1, 128.5, 128.4, 97.0, 72.2, 70.6, 69.4, 68.0, 63.0, 55.7, 49.7, 45.2, 37.6, 37.3, 30.1, 28.7, 27.9, 23.5; HRMS (ESI): *m/z* $[M + H]^+$ calcd for $C_{38}H_{43}N_2O_{11}$:703.2867, Found: 703.2888.

Compound **8b.** R_f = 0.2-0.3 (EtOAc); thick liquid (48% yield); IR ($CHCl_3$) ν 3364, 1727, 1627, 1448, 1251, 1096, 700 cm^{-1}; HRMS (ESI): *m/z* $[M + Na]^+$ calcd for $C_{44}H_{44}N_2NaO_{12}$:815.2972, Found: 815.294.

Compound **2ab.** R_f = 0.2 (EtOAc); thick liquid (56% yield); IR ($CHCl_3$) ν 3289, 1705, 1657, 1532, 1251 cm^{-1}; ^1H NMR (400 MHz, $CDCl_3$) δ 5.69 (br s, 1H), 4.09 (t, *J* = 6.9 Hz, 2H), 3.31 (dt, *J* = 13.0, 6.7 Hz, 4H), 3.08 (q, *J* = 6.3 Hz, 2H), 2.51 – 2.41 (m, 2H), 2.34 (t, *J* = 8.1 Hz, 2H), 2.03 – 1.96 (m, 2H), 1.95 (t, *J* = 2.6 Hz, 1H), 1.64 (p, *J* = 6.4 Hz, 2H); ^{13}C NMR (100 MHz, $CDCl_3$) δ 175.7, 156.3, 80.4, 69.8, 62.4, 47.3, 39.6, 37.6, 30.9, 27.0, 19.4, 18.0; HRMS (ESI): *m/z* $[M + Na]^+$ calcd for $C_{12}H_{18}N_2NaO_3$:261.1214, Found: 261.1220.

Compound **9b.** R_f = 0.2 (EtOAc); white solid (62% yield), mp 85-87 °C; IR ($CHCl_3$) ν 3306, 1727, 1660, 1533, 1492, 1232 cm^{-1}; ^1H NMR (400 MHz, $CDCl_3$) δ 7.06 (d, *J* = 8.1 Hz, 1H), 6.88 (s, 1H), 6.82 (dd, *J* = 8.2, 1.8 Hz, 1H), 6.10 (br s, 1H), 3.41 – 3.33 (m, 4H), 3.22 – 3.14 (m, 2H), 2.43 – 2.35 (m, 2H), 2.21 (s, 3H), 2.20 (s, 3H), 2.07 – 1.97 (m, 2H), 1.77 – 1.68 (m, 2H); ^{13}C NMR (100 MHz, $CDCl_3$) δ 175.9, 155.2, 149.1, 137.6, 133.4, 130.1, 122.7, 118.8, 47.3, 39.5, 37.7, 30.9, 26.9, 19.9, 19.2, 18.0; HRMS (ESI): *m/z* $[M + Na]^+$ calcd for $C_{16}H_{22}N_2NaO_3$:313.1528, Found: 313.1535.

Compound **10b.** R_f = 0.2 (EtOAc); thick liquid (46% yield); IR ($CHCl_3$) ν 3322, 1669 (br), 1524, 1441, 1249 cm^{-1}; ^1H NMR (400 MHz, $CDCl_3$) δ 5.53 (br s, 1H), 5.35 – 5.27 (m, 1H), 5.09 – 5.03 (m, 1H), 4.54 (d, *J* = 6.9 Hz, 2H), 3.38 – 3.29 (m, 4H), 3.11 (q, *J* = 6.3 Hz, 2H), 2.37 (t, *J* = 8.0 Hz, 2H), 2.11 – 1.97 (m, 6H), 1.71 – 1.62

(m, 8H), 1.57 (s, 3H); ^{13}C NMR (100 MHz, $CDCl_3$) δ 175.7, 156.9, 141.5, 131.8, 123.9, 123.9, 119.1, 61.7, 47.3, 39.6, 37.6, 31.0, 29.8, 27.1, 26.4, 25.8, 18.0, 17.8, 16.5; HRMS (ESI): *m/z* $[M + Na]^+$ calcd for $C_{18}H_{30}N_2NaO_3$:345.2153, Found: 345.2154.

Compound **11b.** R_f = 0.2 (EtOAc); thick liquid (53% yield); $[\alpha]_D^{25}$ = -48.04 (c 1.7, CH_2Cl_2); IR ($CHCl_3$) ν 3325, 1715, 1666, 1528, 1376, 1250, 1060, 856 cm^{-1}; ^{1}H NMR (400 MHz, $CDCl_3$) δ 5.69 (t, *J* = 5.9 Hz, 1H), 4.70 (d, *J* = 5.8 Hz, 1H), 4.58 (d, *J* = 5.8 Hz, 1H), 4.21 (t, *J* = 7.3 Hz, 2H), 4.08 (d, *J* = 7.3 Hz, 2H), 4.00 (d, *J* = 9.7 Hz, 1H), 3.34 (d, *J* = 8.7 Hz, 2H), 3.30 (dd, *J* = 7.0, 6.2 Hz, 2H), 3.09 (dd, *J* = 7.5, 6.1 Hz, 2H), 2.35 (t, *J* = 8.1 Hz, 2H), 2.06 – 1.94 (m, 2H), 1.69 – 1.60 (m, 2H), 1.39 (s, 6H), 1.32 (s, 3H), 1.27 (s, 3H); ^{13}C NMR (100 MHz, $CDCl_3$) δ 175.7, 156.2, 113.6, 112.7, 111.8, 85.1, 83.1, 82.3, 69.8, 64.6, 47.3, 39.6, 37.7, 30.9, 27.0, 26.5, 26.5, 26.3, 25.2, 18.0; HRMS (ESI): *m/z* $[M + Na]^+$ calcd for $C_{20}H_{32}N_2NaO_8$:451.2056, Found: 451.2054.

Compound **12b.** R_f = 0.2 (EtOAc); thick liquid (63% yield); $[\alpha]_D^{25}$ = +19.18 (c 0.39, CH_2Cl_2); IR ($CHCl_3$) ν 3328, 1716, 1670, 1522, 1249, 1090, 742 cm^{-1}; ^{1}H NMR (400 MHz, $CDCl_3$) δ 7.41 – 7.27 (m, 15H), 5.84 – 5.71 (m, 1H), 5.52 – 5.44 (m, 1H), 5.03 – 4.94 (m, 2H), 4.92 (d, *J* = 10.6 Hz, 1H), 4.81 (s, 1H), 4.73 (d, *J* = 3.1 Hz, 2H), 4.64 – 4.58 (m, 3H), 4.38 – 4.31 (m, 2H), 3.91 (d, *J* = 5.8 Hz, 2H), 3.80 – 3.74 (m, 2H), 3.65 (dt, *J* = 9.7, 6.6 Hz, 1H), 3.39 – 3.28 (m, 5H), 3.18 – 3.09 (m, 2H), 2.37 (t, *J* = 8.1 Hz, 2H), 2.10 – 1.96 (m, 4H), 1.72 – 1.57 (m, 4H); ^{13}C NMR (100 MHz, $CDCl_3$) δ 175.6, 156.5, 138.5, 138.4, 138.3, 138.1, 128.5, 128.4, 128.4, 128.3, 128.0, 127.8, 127.7, 127.6, 115.0, 98.0, 80.2, 75.3, 74.7, 72.7, 72.2, 70.5, 67.1, 64.0, 47.3, 39.8, 38.0, 31.0, 30.3, 28.6, 27.3, 18.0; HRMS (ESI): *m/z* $[M + Na]^+$ calcd for $C_{40}H_{50}N_2NaO_8$:709.3464, Found: 709.3459.

Compound **13b.** R_f = 0.2 (EtOAc); thick liquid (67% yield); $[\alpha]_D^{25}$ = +41.82 (c 1.48, CH_2Cl_2); IR ($CHCl_3$) ν 3340, 1718, 1669, 1455, 1257, 1106, 746 cm^{-1}; ^{1}H NMR (400 MHz, $CDCl_3$) δ 7.36 – 7.23 (m, 10H), 5.52 (t, *J* = 6.1 Hz, 1H), 4.91 (s, 1H), 4.55 – 4.41 (m, 4H), 4.25 (dd, *J* = 11.1, 3.1 Hz, 1H), 4.21 – 4.10 (m, 2H), 3.96 (dd, *J* = 2.7, 0.7 Hz, 1H), 3.81 (dd, *J* = 6.2, 2.7 Hz, 1H), 3.36 (s, 3H), 3.35 – 3.25 (m, 4H), 3.09 (q, *J* = 6.3 Hz, 2H), 2.36 (t, *J* = 8.1 Hz, 2H), 2.03 – 1.94 (m, 2H), 1.68 – 1.59 (m, 2H); ^{13}C NMR (100 MHz, $CDCl_3$) δ 175.7, 156.3, 137.7, 137.5, 128.5, 128.4, 127.9, 127.9, 127.8, 107.4, 87.9, 83.5, 79.9, 72.3, 72.0, 64.4, 55.0, 47.3, 39.6, 37.8, 30.9, 27.1, 18.0; HRMS (ESI): *m/z* $[M + Na]^+$ calcd for $C_{28}H_{36}N_2NaO_7$:535.242, Found: 535.2417.

Compound **14b** and **14c** (reaction at 60 °C)**.** R_f = 0.2 (EtOAc); thick liquid (64% yield); IR ($CHCl_3$) ν 3321, 1668 (br), 1594, 1252, 850 cm^{-1}; ^{1}H NMR (400 MHz, $CDCl_3$) δ 8.20 – 8.11 (m, 2H), 6.96 – 6.85 (m, 2H), 5.62 – 5.49 (m, 2H), 4.06 – 3.93 (m, 6H), 3.41 – 3.27 (m, 10H), 3.14 – 3.03 (m, 4H), 2.41 – 2.31 (m, 4H), 2.01 (qd, *J* = 7.5, 3.9 Hz, 4H), 1.87 – 1.72 (m, 4H), 1.70 – 1.61 (m, 4H), 1.55 (s, 4H), 1.47 – 1.34 (m, 4H), 1.35 – 1.19 (m, 20H); ^{13}C NMR (100 MHz, $CDCl_3$) δ 175.7, 164.3, 157.0, 141.3, 126.0, 114.5, 68.9, 64.9, 47.3, 39.6, 37.5, 34.2, 32.9, 31.0, 29.7, 29.5, 29.4, 29.3, 29.1, 29.0, 28.8, 28.2, 27.0, 25.9, 18.0; HRMS (ESI) for **14b**: *m/z* $[M + H]^+$ calcd for $C_{18}H_{34}BrN_2O_3$:405.1753, Found: 405.1756. HRMS (ESI) for **14c**: *m/z* $[M + H]^+$ calcd for $C_{24}H_{38}N_3O_6$:464.2761, Found: 464.2770.

Compound **14b** and **14c** (reaction at rt)**.** Thick liquid, (60% yield); ^{1}H NMR (400 MHz, $CDCl_3$) δ 8.22 – 8.10 (m, 1H), 6.91 (dd, *J* = 7.3, 5.1 Hz, 1H), 5.52 (br s, 2H), 4.06 – 3.94 (m, 5H), 3.41 – 3.27 (m, 11H), 3.10 (dd, *J* = 12.4, 6.2 Hz, 4H), 2.37 (t, *J* = 8.1 Hz, 4H), 2.06 – 1.96 (m, 4H), 1.87 – 1.77 (m, 4H), 1.70 – 1.62 (m, 4H), 1.61 – 1.52 (m, 4H), 1.44 – 1.36 (m, 4H), 1.26 (s, 20H); ^{13}C NMR (100 MHz, $CDCl_3$) δ 175.7, 164.3, 157.0, 141.4, 126.0, 114.5, 69.0, 64.9, 47.3, 39.6, 37.5, 34.1, 32.9, 31.0, 29.5, 29.4, 29.3, 29.1, 28.8, 28.2, 27.1, 25.9, 18.0;

Compound **15b.** R_f = 0.2 (EtOAc); thick liquid (41% yield); IR ($CHCl_3$) ν 3340, 1671, 1592, 1522, 1210, 816 cm^{-1}; ^{1}H NMR (400 MHz, $CDCl_3$) δ 5.63 (br s, 1H), 4.22 – 4.15 (m, 2H), 3.73 (t, *J* = 6.0 Hz, 2H), 3.70 – 3.66 (m, 2H), 3.60 (t, *J* = 5.7 Hz, 2H), 3.38 – 3.29 (m, 4H), 3.11 (q, *J* = 6.3 Hz, 2H), 2.37 (t, *J* = 8.0 Hz, 2H), 2.06 – 1.97 (m, 2H), 1.70 – 1.63 (m, 2H); ^{13}C NMR (100 MHz, $CDCl_3$) δ 175.7, 156.5, 71.3, 69.7, 63.8, 47.3, 42.8, 39.6, 37.7, 31.0, 27.1, 18.0; HRMS (ESI): *m/z* $[M + Na]^+$ calcd for $C_{12}H_{21}ClN_2NaO_4$:315.1087, Found: 315.1093.

Structural data of carbonates A, B, C

Table 3, compound **(A).** R_f = 0.5 (hexane/EtOAc, 85:15); white solid (82% yield), mp 53-54 °C; IR ($CHCl_3$) ν 3292, 1766, 1595, 1491, 1233, 1016 cm^{-1}; ^{1}H NMR (400 MHz, $CDCl_3$) δ 7.43 – 7.31 (m, 2H), 7.29 – 7.15 (m, 3H), 2.64 (s, 1H), 2.26 – 2.16 (m, 2H), 2.00 – 1.89 (m, 2H), 1.76 – 1.60 (m, 4H), 1.58 – 1.50 (m, 1H), 1.42 – 1.29 (m, 1H); ^{13}C NMR (100 MHz, $CDCl_3$) δ 151.2, 151.1, 129.5, 126.0, 121.3, 82.6, 78.7, 75.4, 36.9, 25.1, 22.7.

Table 3, compound **(B).** R_f = 0.5 (hexane/EtOAc, 85:15); thick liquid; IR ($CHCl_3$) ν 3289, 1744, 1452, 1225, 912 cm^{-1}; ^1H NMR (400 MHz, $CDCl_3$) δ 7.42 – 7.31 (m, 5H), 5.16 (s, 2H), 2.64 (s, 1H), 2.24 – 2.15 (m, 2H), 1.92 – 1.83 (m, 2H), 1.73 – 1.60 (m, 4H), 1.58 – 1.49 (m, 1H), 1.38 – 1.26 (m, 1H); ^{13}C NMR (100 MHz, $CDCl_3$) δ 155.1, 135.3, 128.6, 128.5, 128.5, 83.0, 77.7, 74.9, 69.3, 36.8, 25.0, 22.6.

Table 3, compound **(C).** R_f = 0.5 (hexane/EtOAc, 85:15); thick liquid; IR ($CHCl_3$) ν 3285, 1747, 1369, 1271, 1231, 1014; ^1H NMR (400 MHz, $CDCl_3$) δ 4.19 (t, *J* = 10.7 Hz, 2H), 2.62 (s, 1H), 2.23 – 2.12 (m, 2H), 1.92 – 1.80 (m, 2H), 1.72 – 1.59 (m, 4H), 1.58 – 1.48 (m, 1H), 1.38 – 1.26 (m, 1H), 1.30 (t, *J* = 7.2 Hz, 3H); ^{13}C NMR (100 MHz, $CDCl_3$) δ 153.0, 83.2, 77.4, 74.8, 63.7, 37.0, 25.1, 22.7, 14.4.

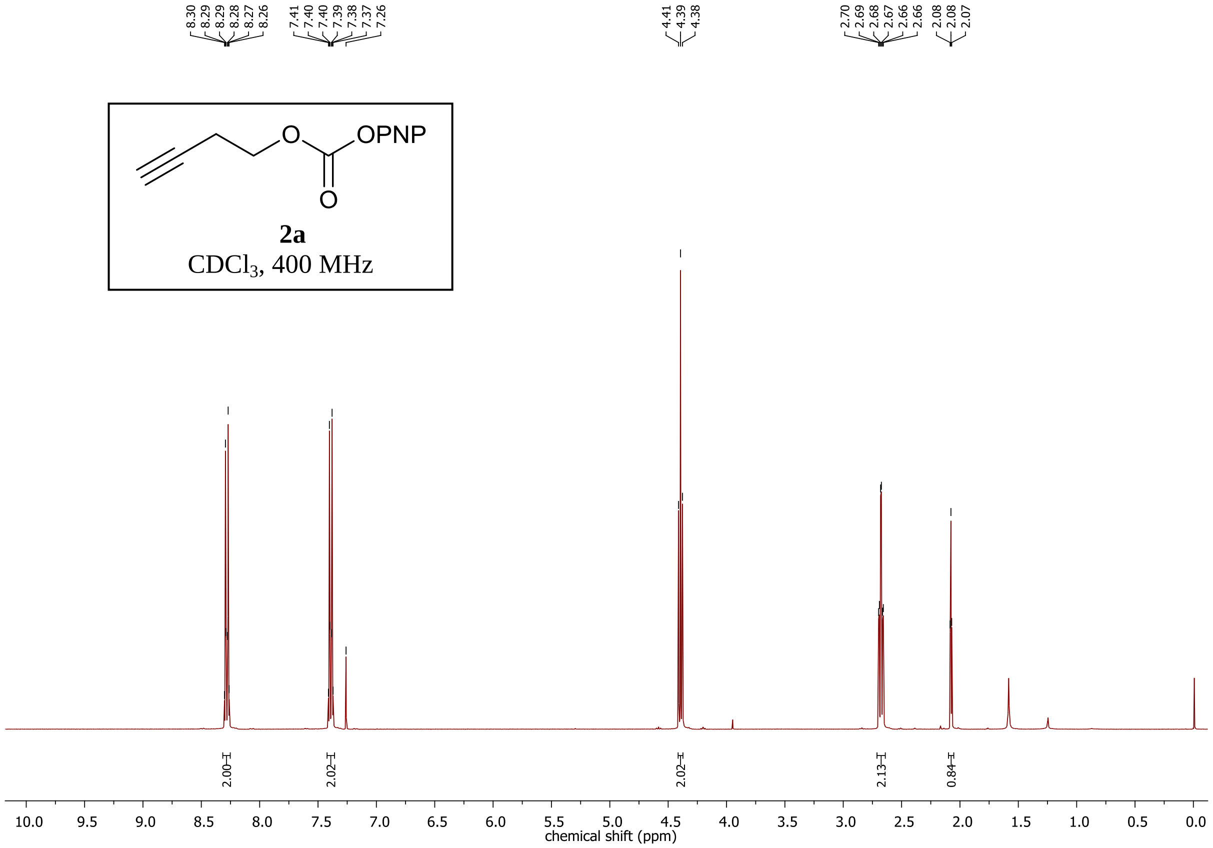

8.30
8.29
8.29
8.28
8.27
8.26
7.41
7.40
7.40
7.39
7.38
7.37
7.26
4.41
4.39
4.38
2.70
2.69
2.68
2.67
2.66
2.66
2.08
2.08
2.07
OPNP
O
O
2a
CDCl3, 400 MHz
2.00
2.02
2.02
2.13
0.84
10.0
9.5
9.0
8.5
8.0
7.5
7.0
6.5
6.0
5.5
5.0
4.5
4.0
3.5
3.0
2.5
2.0
1.5
1.0
0.5
0.0
chemical shift (ppm)

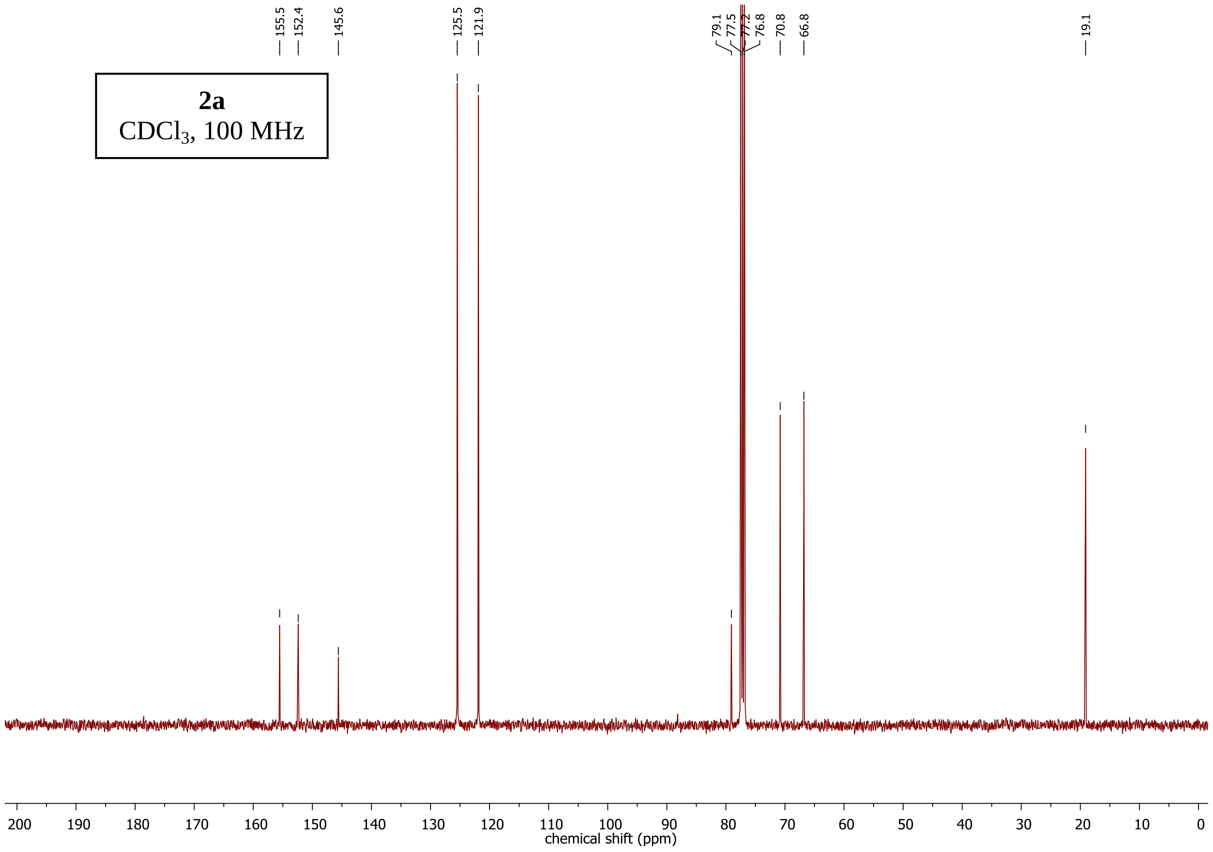

155.5
152.4
145.6
125.5
121.9
79.1
77.5
77.2
76.8
70.8
66.8
19.1
2a
CDCl3, 100 MHz
200
190
180
170
160
150
140
130
120
110
100
90
80
70
60
50
40
30
20
10
0
chemical shift (ppm)

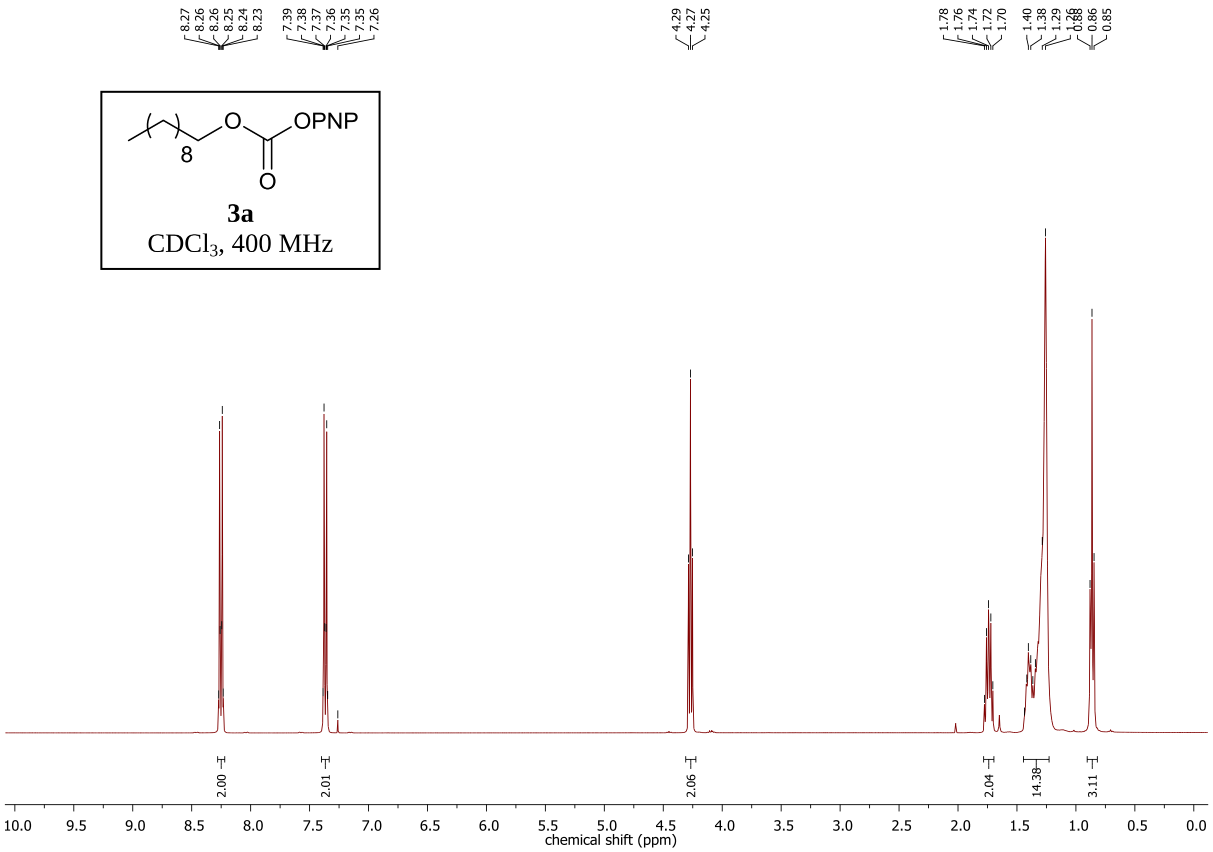
OPNP
8
O
O
O
3a
CDCl3, 400 MHz
2.00
2.01
2.06
2.04
14.38
3.11
chemical shift (ppm)

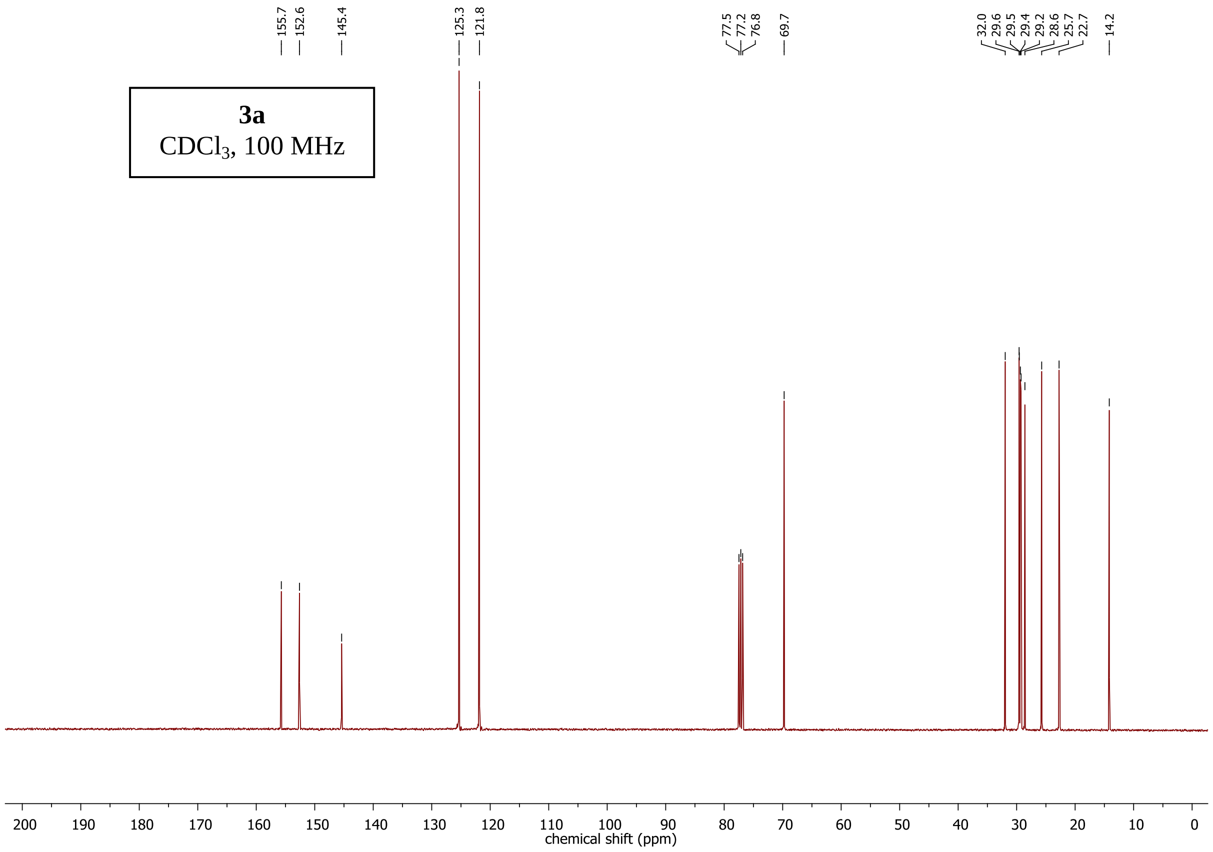
3a
CDCl3, 100 MHz
155.7
152.6
145.4
125.3
121.8
77.5
77.2
76.8
69.7
32.0
29.6
29.5
29.4
29.2
28.6
25.7
22.7
14.2
chemical shift (ppm)

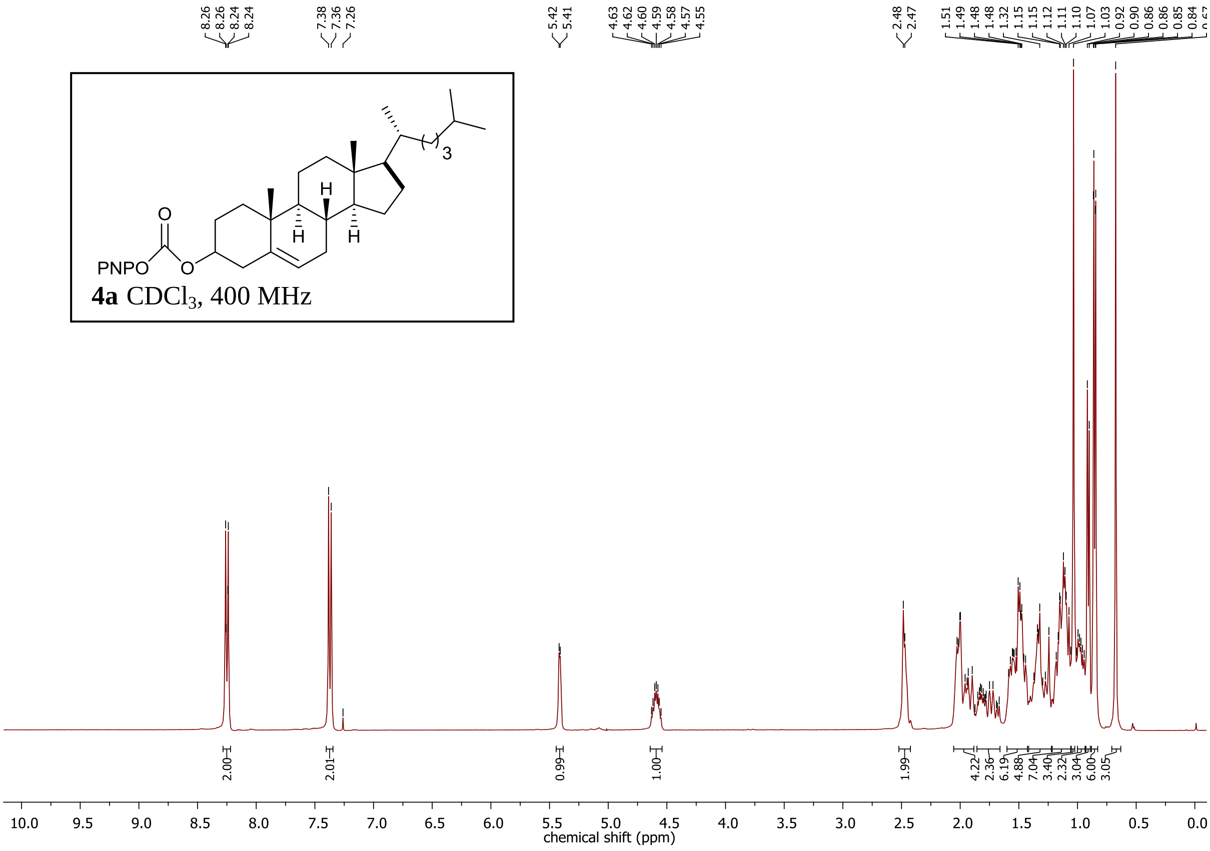
8.26 8.26 8.24 8.24
7.38 7.36 7.26
5.42 5.41
4.63 4.62 4.60 4.59 4.58 4.57 4.55
2.48 2.47
1.51 1.49 1.48 1.48 1.32 1.15 1.15 1.12 1.11 1.10 1.07 1.03 0.92 0.90 0.86 0.85 0.84
PNPO
O
O
H
H
H
3
4a CDCl3, 400 MHz
2.00
2.01
0.99
1.00
1.99
4.22 2.36 6.19 4.88 7.04 3.40 2.32 3.04 6.00 3.05
10.0 9.5 9.0 8.5 8.0 7.5 7.0 6.5 6.0 5.5 5.0 4.5 4.0 3.5 3.0 2.5 2.0 1.5 1.0 0.5 0.0
chemical shift (ppm)

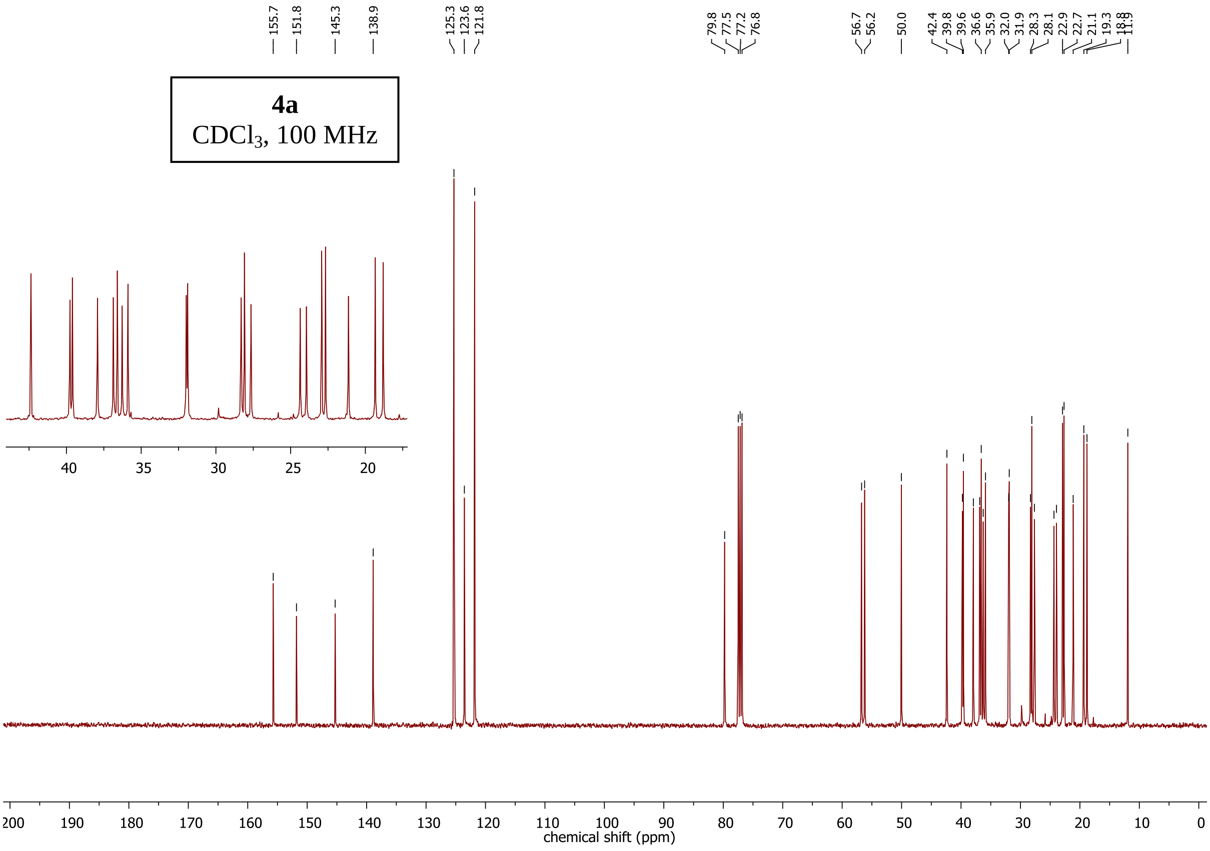
155.7
151.8
145.3
138.9
125.3 123.6 121.8
79.8 77.5 77.2 76.8
56.7 56.2
50.0
42.4 39.8 39.6 36.6 35.9 32.0 31.9 28.3 28.1 22.9 22.7 21.1 19.3 18.8 11.9
4a
CDCl3, 100 MHz
40 35 30 25 20
200 190 180 170 160 150 140 130 120 110 100 90 80 70 60 50 40 30 20 10 0
chemical shift (ppm)

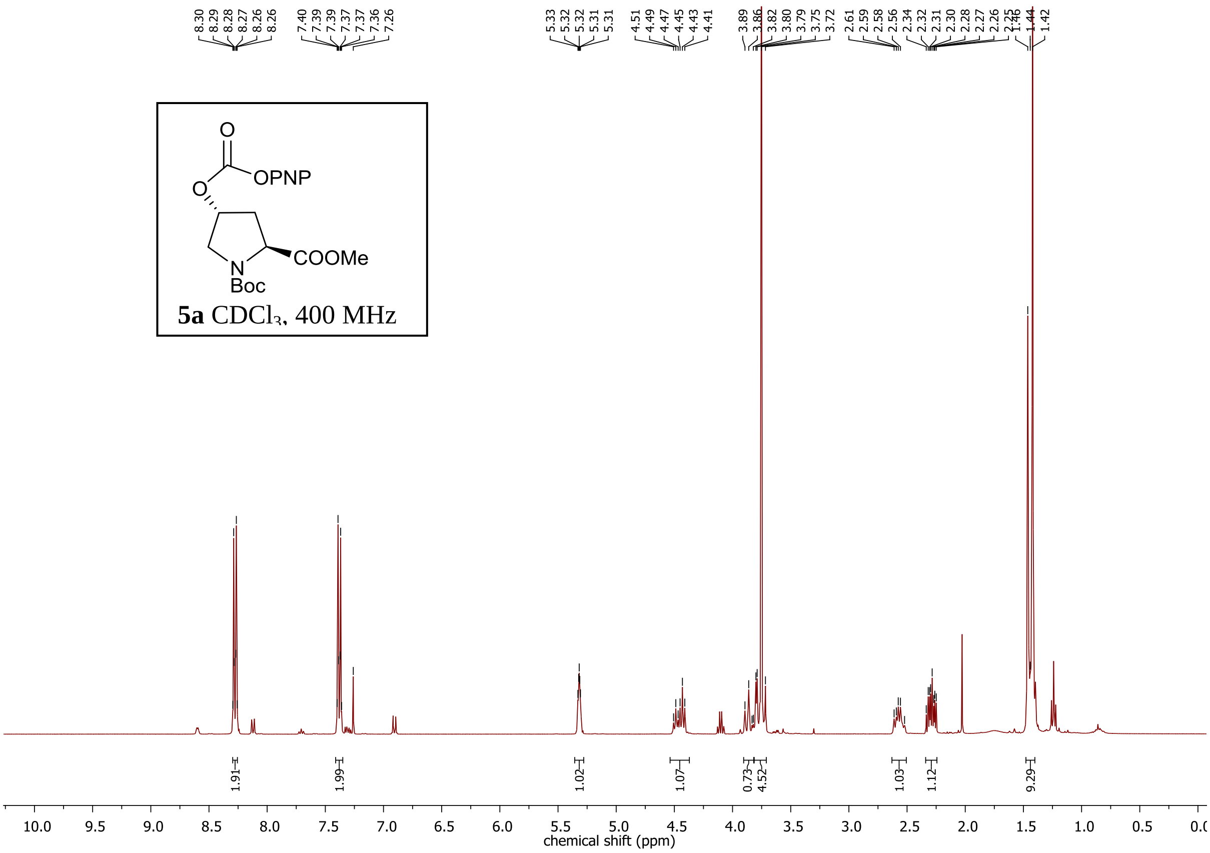
OPNP
COOMe
N
Boc
5a CDCl3, 400 MHz
chemical shift (ppm)

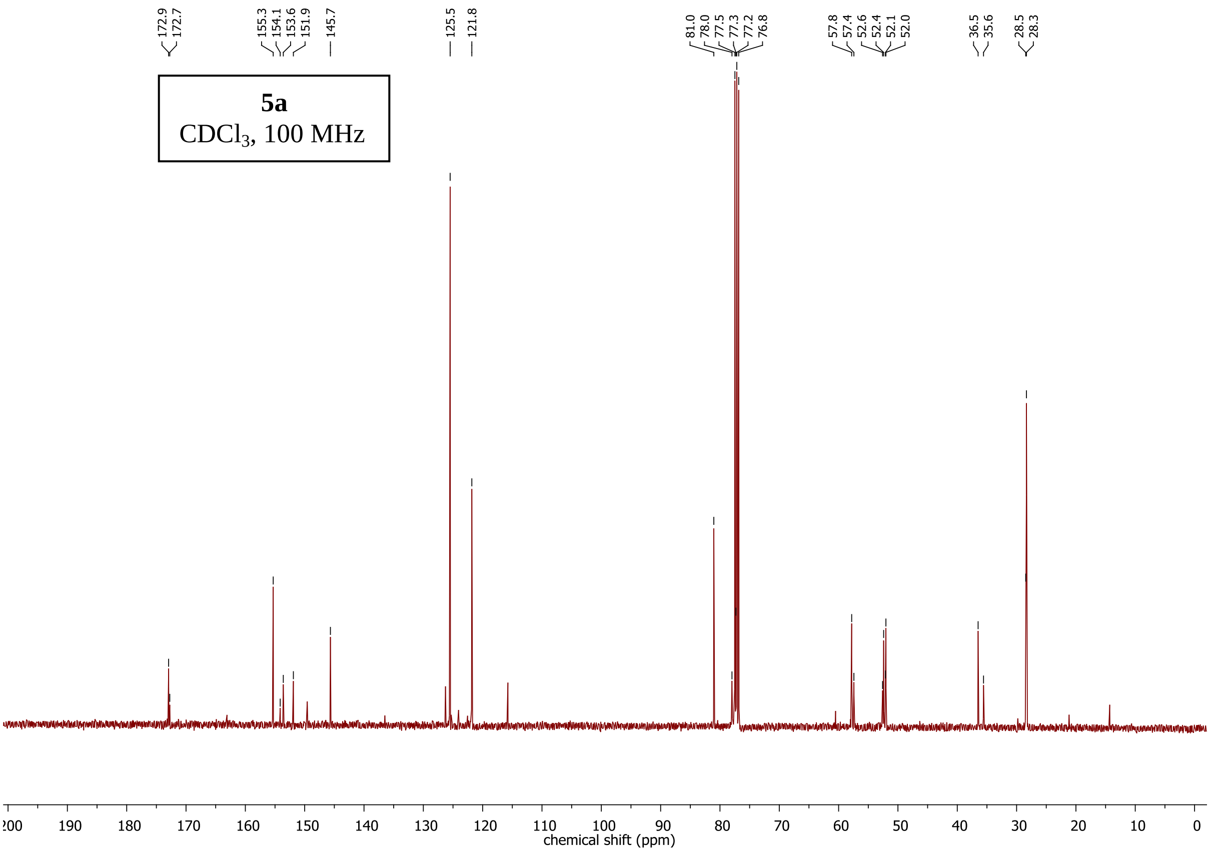
5a
CDCl3, 100 MHz
chemical shift (ppm)

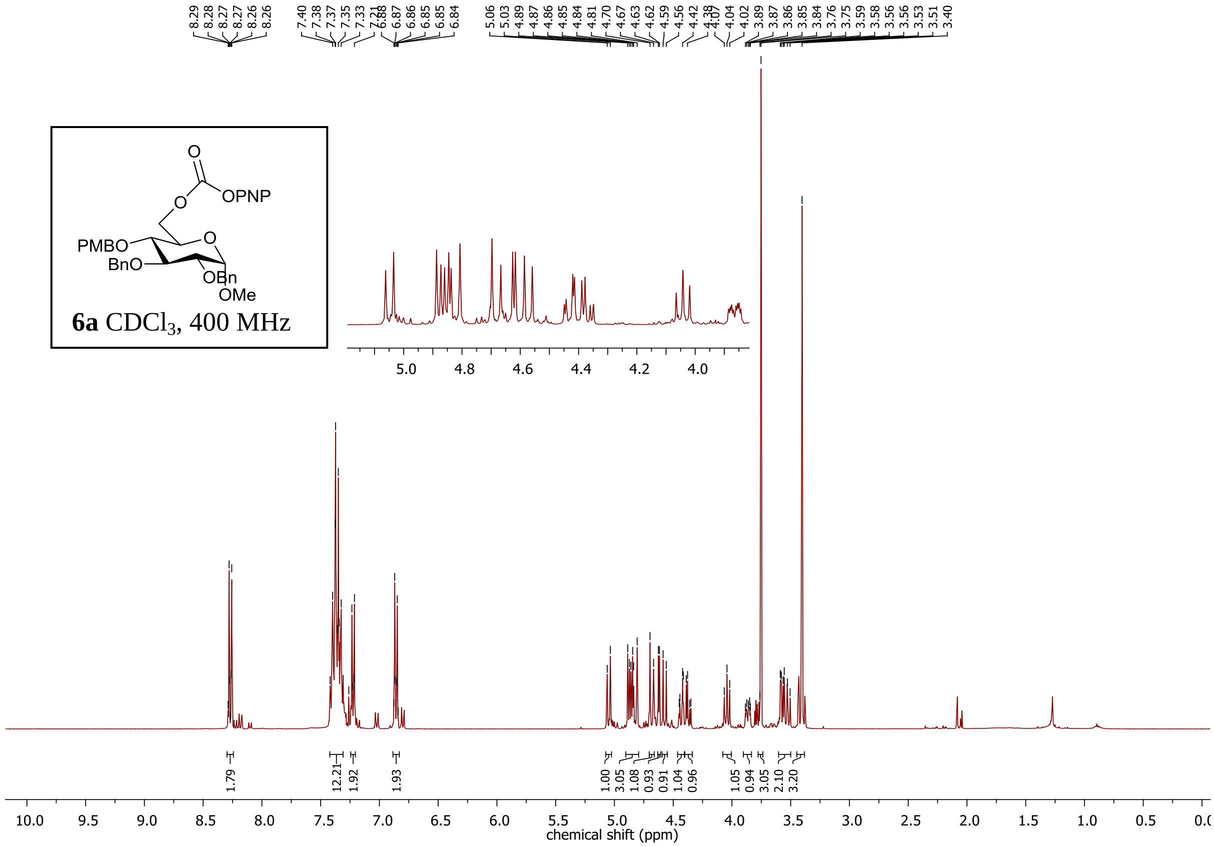
8.29
8.28
8.27
8.27
8.26
8.26
7.40
7.38
7.37
7.35
7.33
7.21
6.88
6.87
6.86
6.85
6.85
6.84
5.06
5.03
4.89
4.87
4.86
4.85
4.84
4.81
4.70
4.67
4.63
4.62
4.59
4.56
4.42
4.38
4.07
4.04
4.02
3.89
3.87
3.86
3.85
3.84
3.76
3.75
3.59
3.58
3.56
3.53
3.51
3.40
O
O
OPNP
O
PMBO
BnO
OBn
OMe
6a CDCl3, 400 MHz
5.0
4.8
4.6
4.4
4.2
4.0
1.79
12.21
1.92
1.93
1.00
3.05
1.08
0.93
0.91
1.04
0.96
1.05
0.94
3.05
2.10
3.20
10.0 9.5 9.0 8.5 8.0 7.5 7.0 6.5 6.0 5.5 5.0 4.5 4.0 3.5 3.0 2.5 2.0 1.5 1.0 0.5 0.0
chemical shift (ppm)

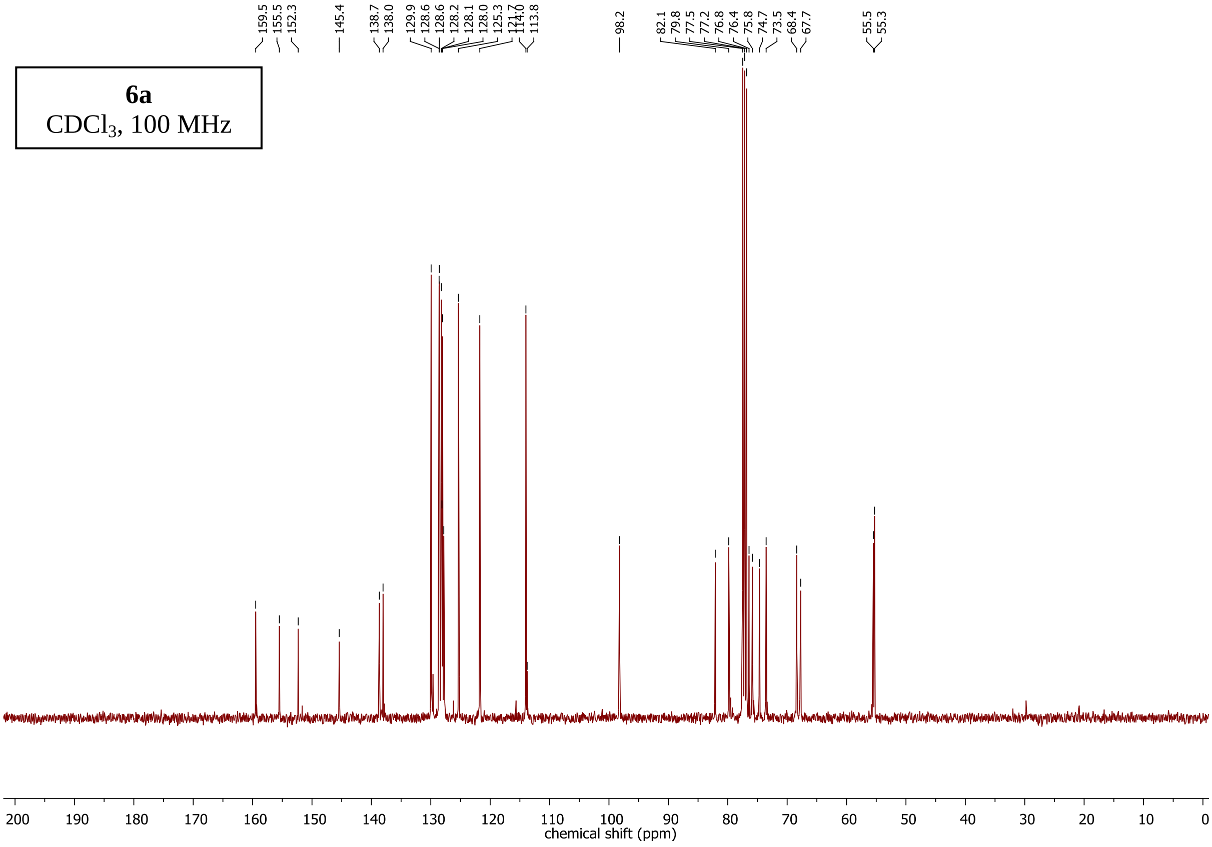
159.5
155.5
152.3
145.4
138.7
138.0
129.9
128.6
128.6
128.2
128.1
128.0
125.3
121.7
114.0
113.8
98.2
82.1
79.8
77.5
77.2
76.8
76.4
75.8
74.7
73.5
68.4
67.7
55.5
55.3
6a
CDCl3, 100 MHz
200 190 180 170 160 150 140 130 120 110 100 90 80 70 60 50 40 30 20 10 0
chemical shift (ppm)

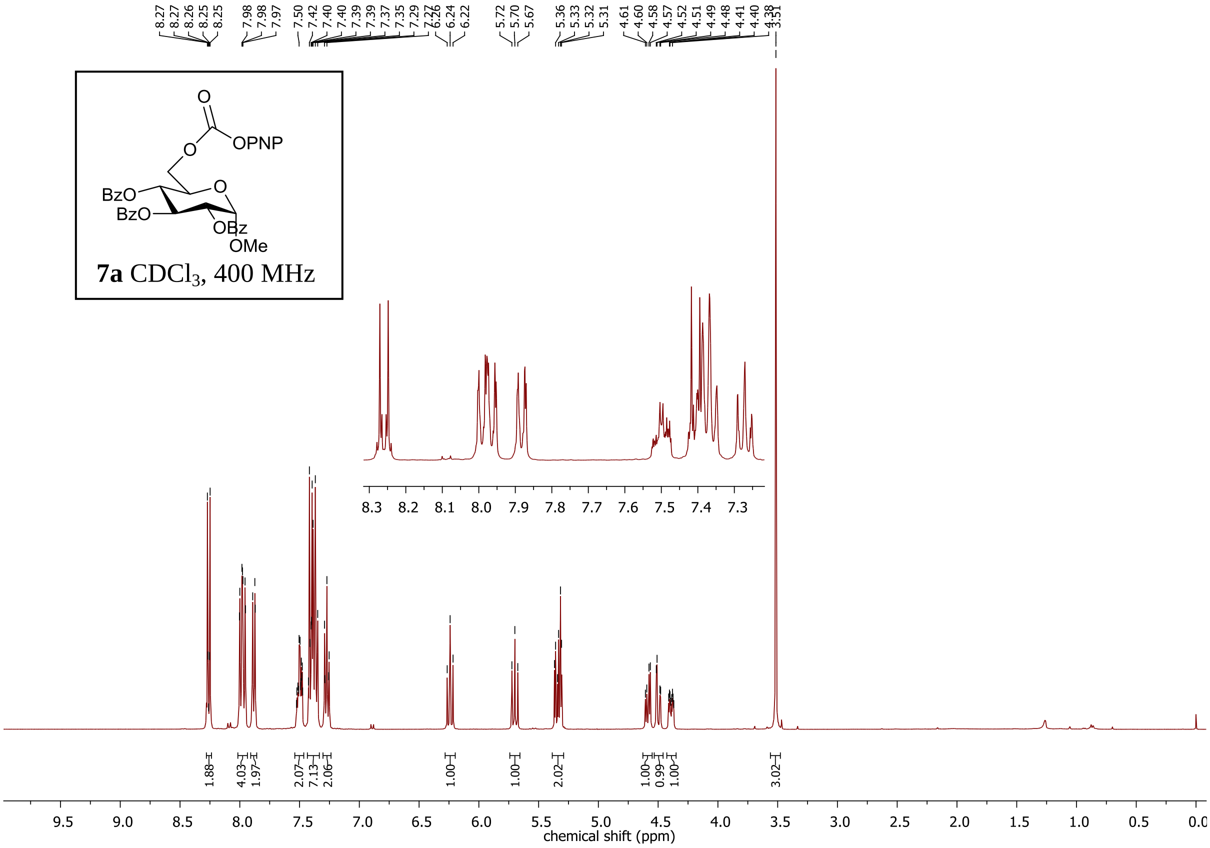

OPNP
BzO
BzO
OBz
OMe
7a CDCl3, 400 MHz
chemical shift (ppm)

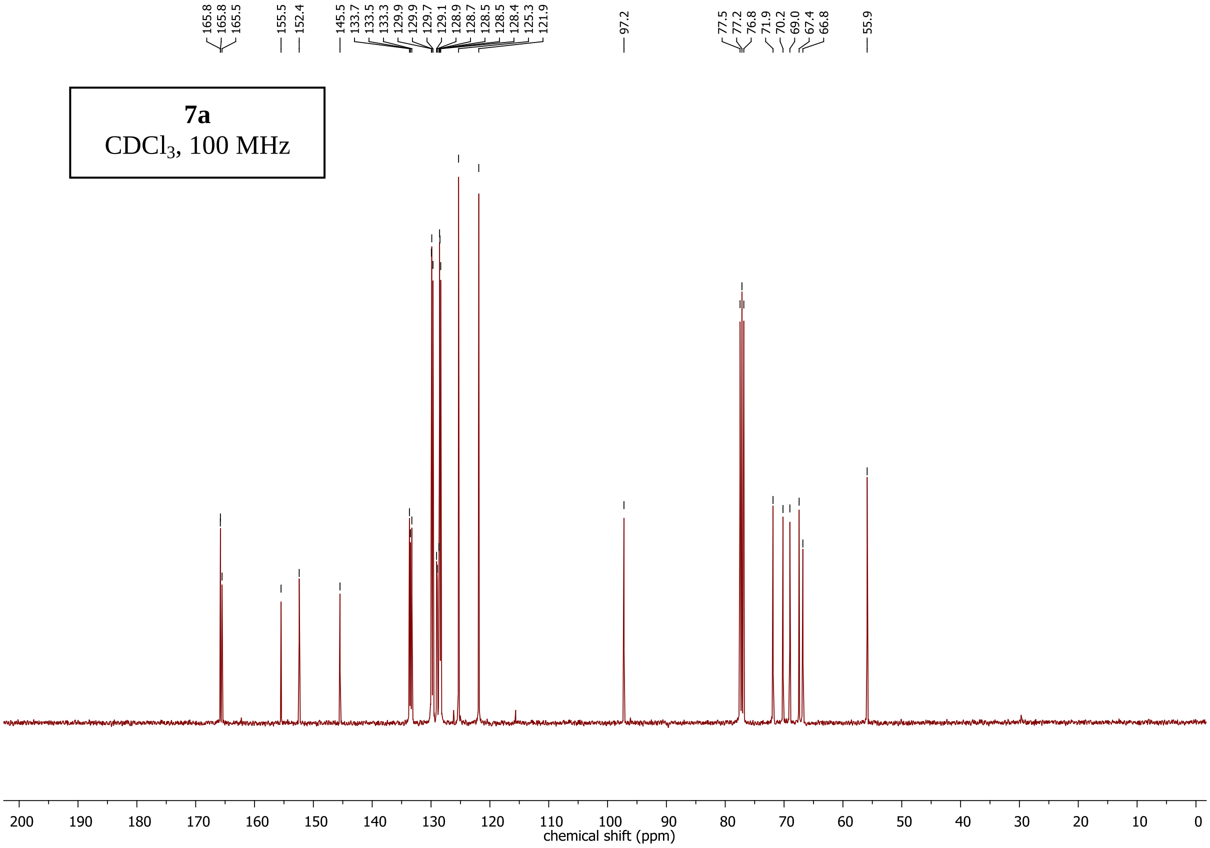

7a
CDCl3, 100 MHz
chemical shift (ppm)

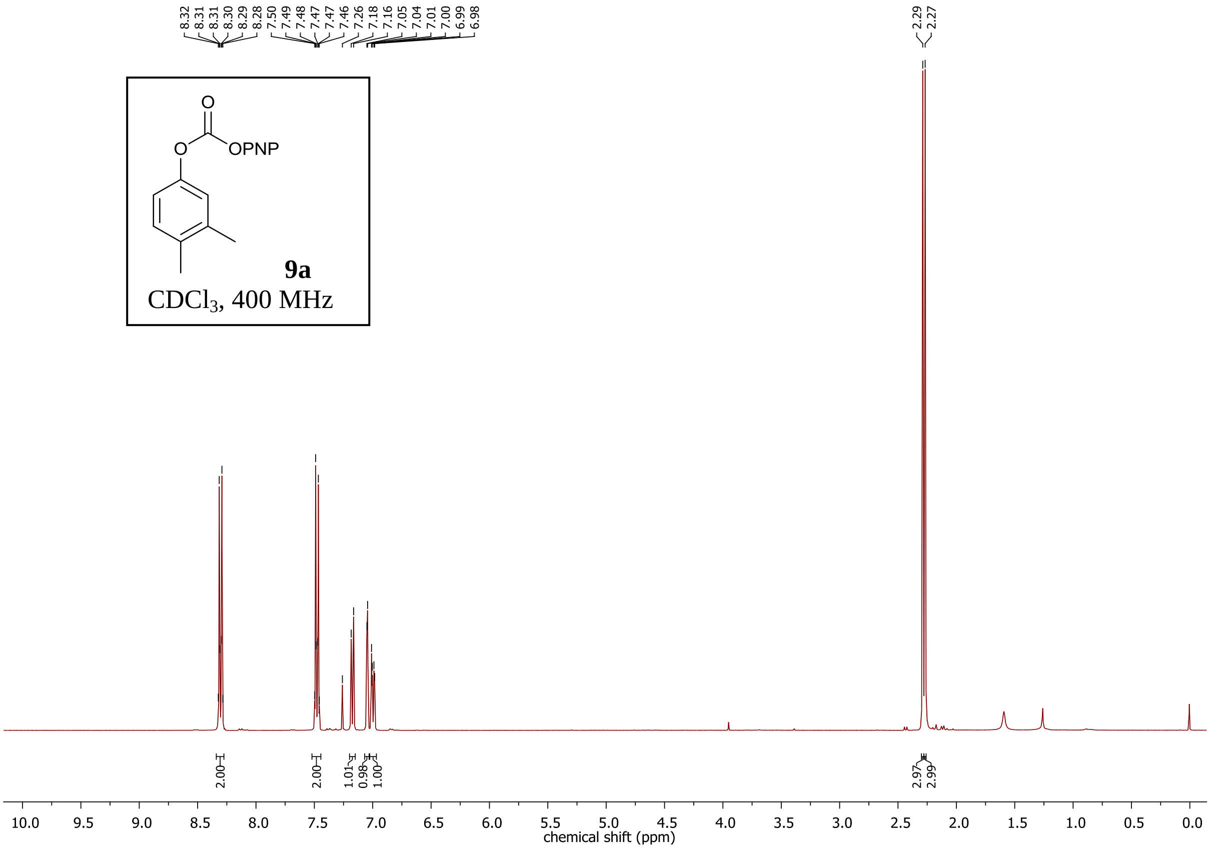
8.32 8.31 8.31 8.30 8.29 8.28 7.50 7.49 7.48 7.47 7.47 7.46 7.26 7.18 7.16 7.05 7.04 7.01 7.00 6.99 6.98
2.29 2.27
O
O
OPNP
9a
CDCl3, 400 MHz
2.00 2.00 1.01 0.98 1.00 2.97 2.99
10.0 9.5 9.0 8.5 8.0 7.5 7.0 6.5 6.0 5.5 5.0 4.5 4.0 3.5 3.0 2.5 2.0 1.5 1.0 0.5 0.0
chemical shift (ppm)

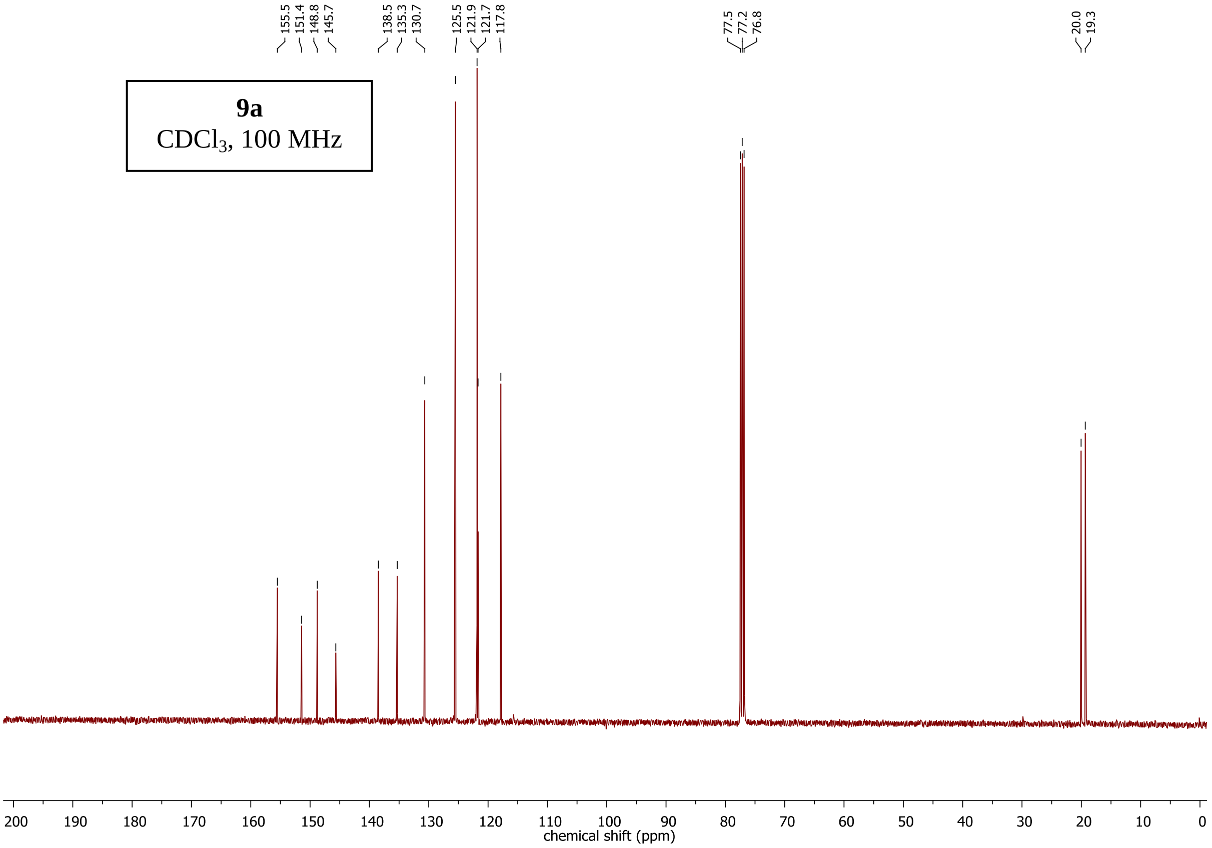
155.5 151.4 148.8 145.7 138.5 135.3 130.7 125.5 121.9 121.7 117.8
77.5 77.2 76.8
20.0 19.3
9a
CDCl3, 100 MHz
200 190 180 170 160 150 140 130 120 110 100 90 80 70 60 50 40 30 20 10 0
chemical shift (ppm)

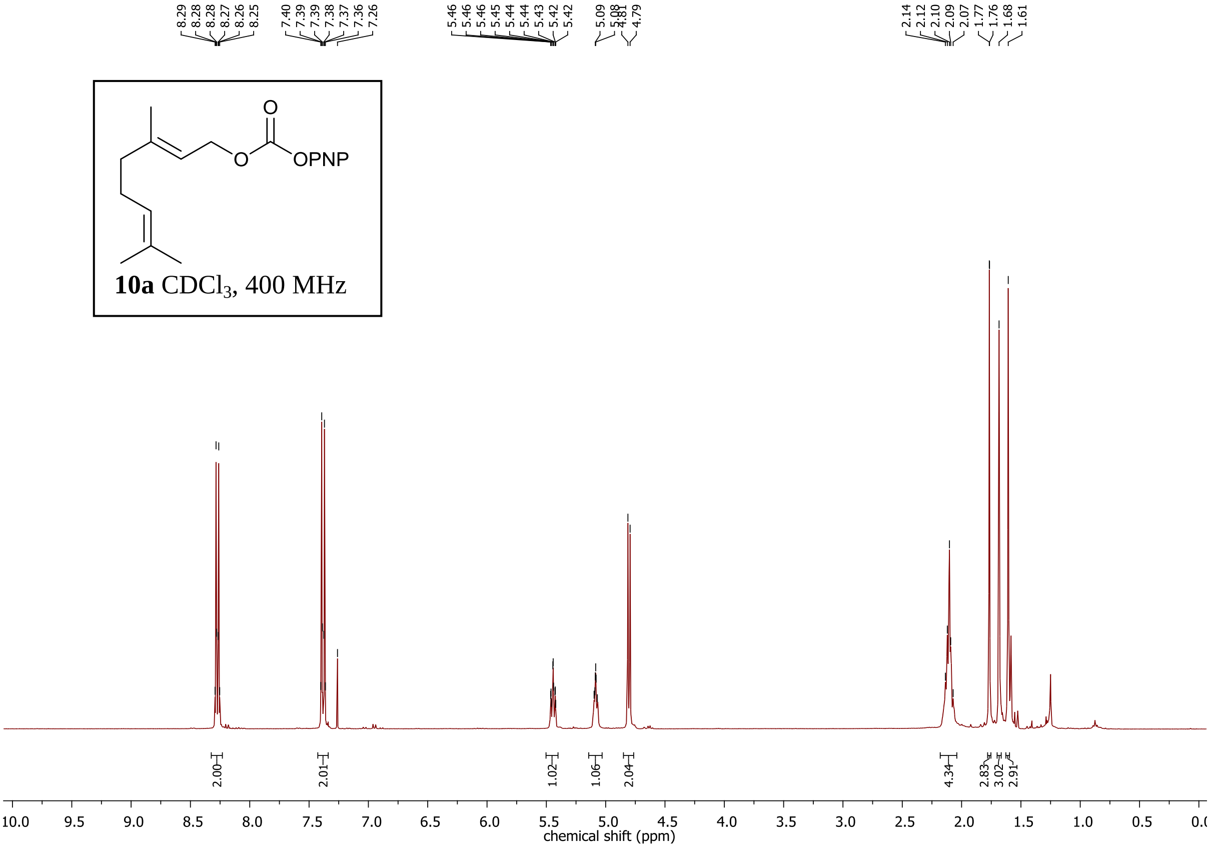
OPNP
O
O
10a CDCl3, 400 MHz
8.29
8.28
8.28
8.27
8.26
8.25
7.40
7.39
7.39
7.38
7.37
7.36
7.26
5.46
5.46
5.46
5.45
5.44
5.44
5.43
5.42
5.42
5.09
5.08
4.81
4.79
2.14
2.12
2.10
2.09
2.07
1.77
1.76
1.68
1.61
2.00
2.01
1.02
1.06
2.04
4.34
2.83
3.02
2.91
chemical shift (ppm)

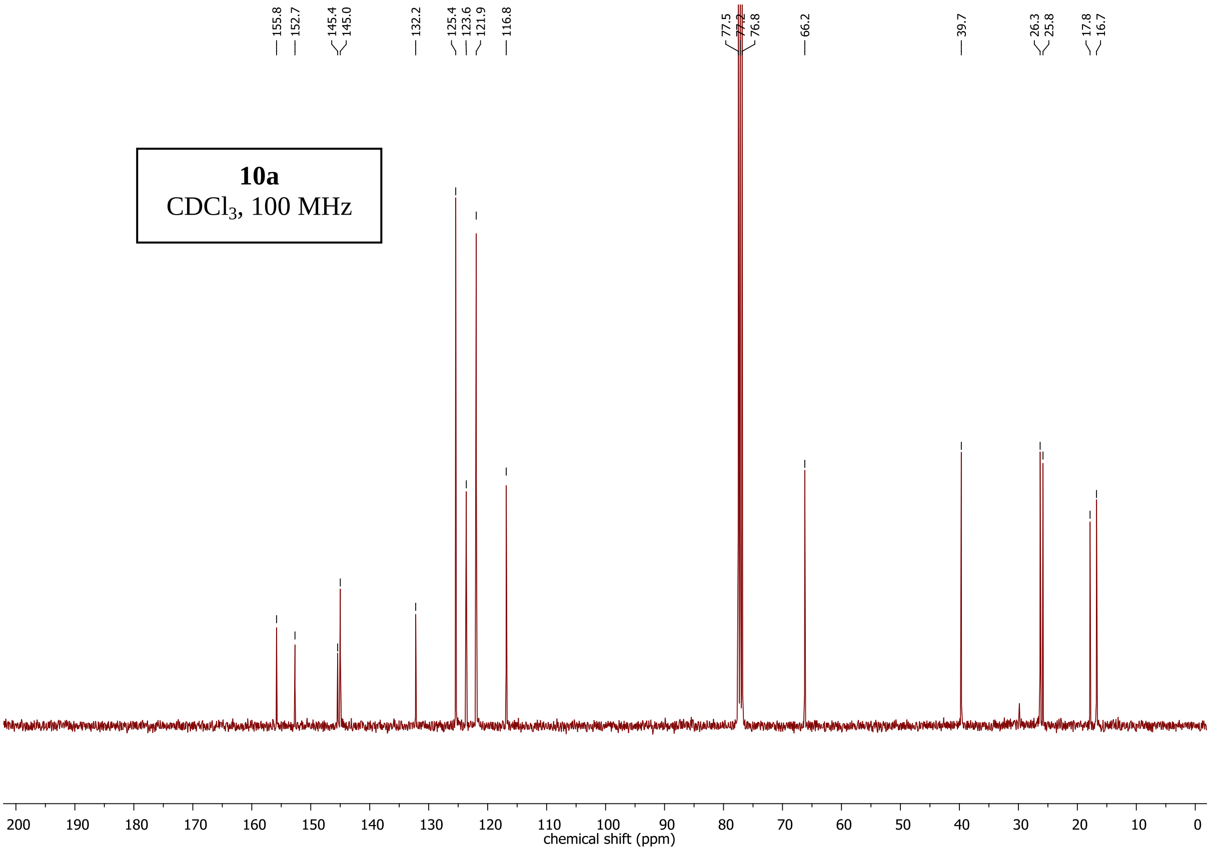
10a
CDCl3, 100 MHz
155.8
152.7
145.4
145.0
132.2
125.4
123.6
121.9
116.8
77.5
77.2
76.8
66.2
39.7
26.3
25.8
17.8
16.7
chemical shift (ppm)

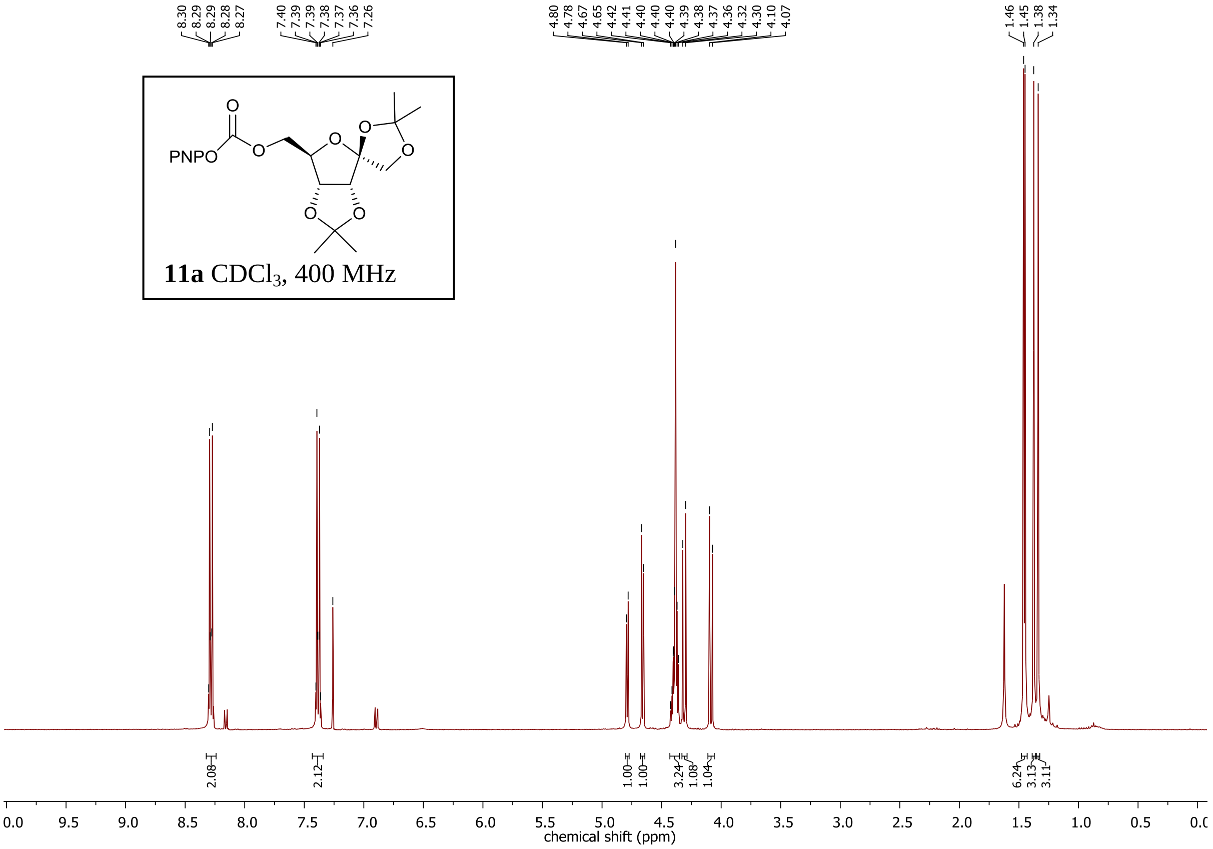
PNPO
11a CDCl$_3$, 400 MHz
chemical shift (ppm)

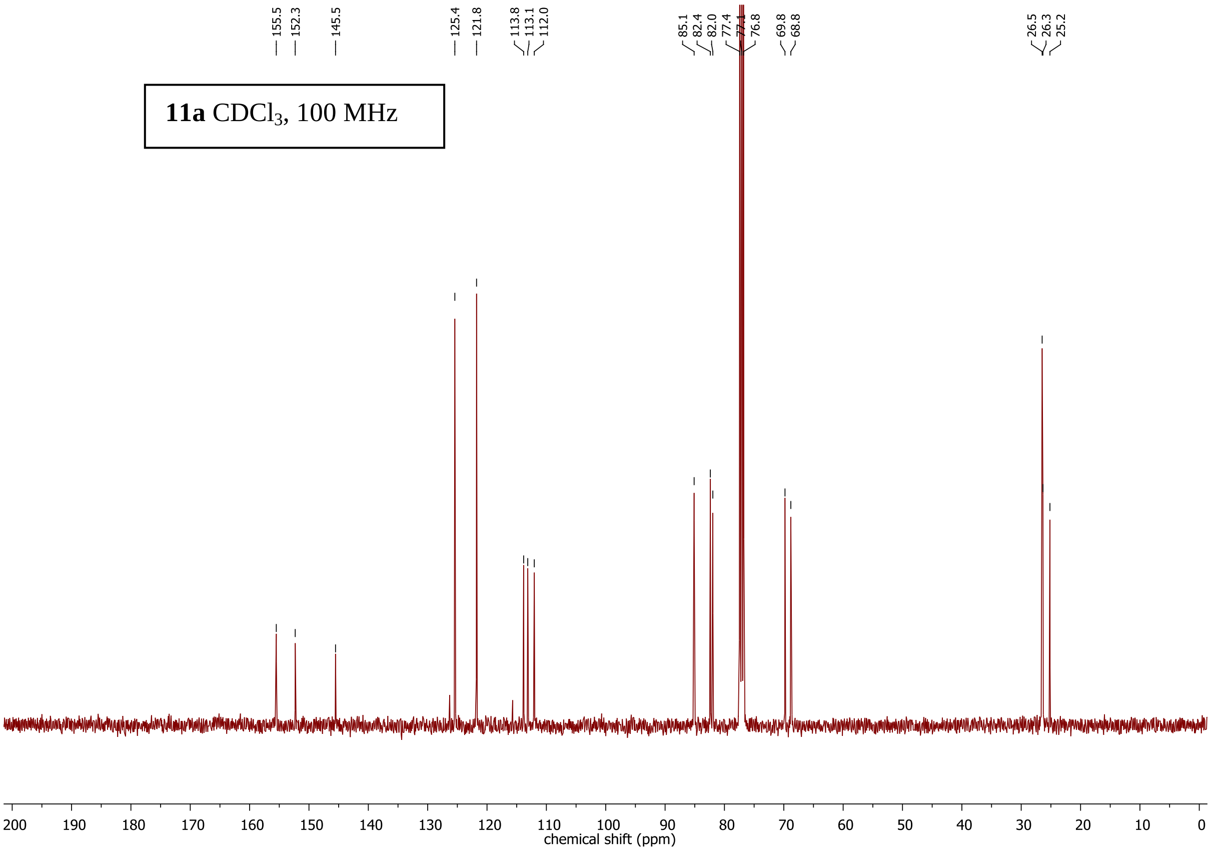
11a CDCl$_3$, 100 MHz
chemical shift (ppm)

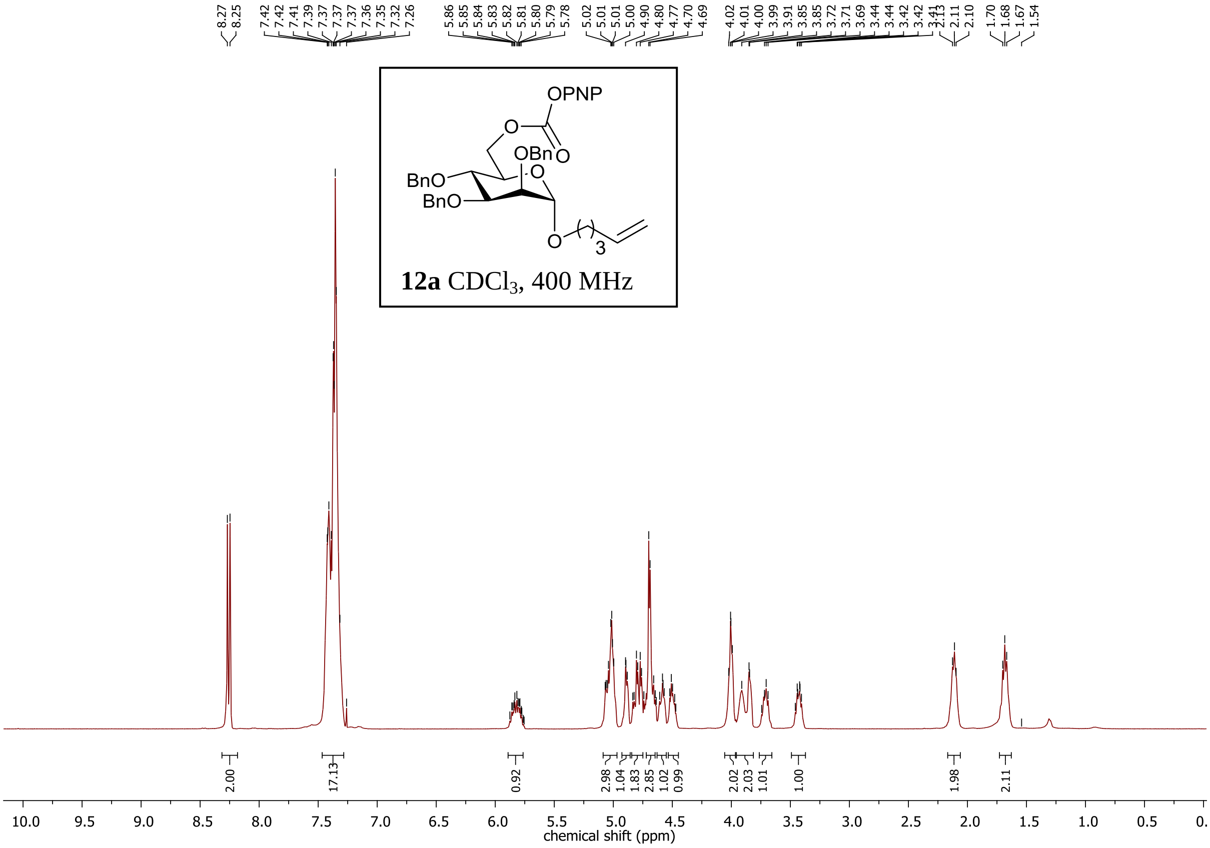

OPNP
OBn
BnO
BnO
3
12a CDCl3, 400 MHz
chemical shift (ppm)

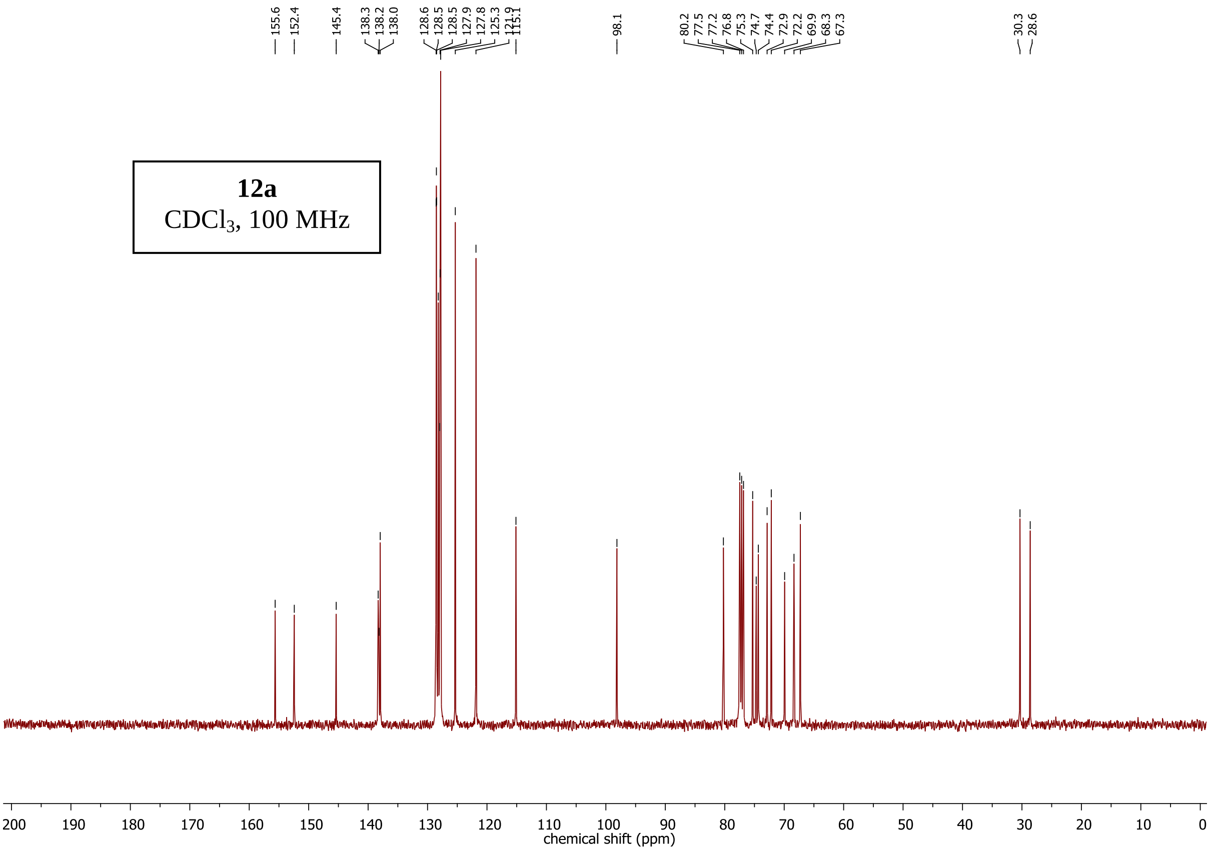

12a
CDCl3, 100 MHz
chemical shift (ppm)

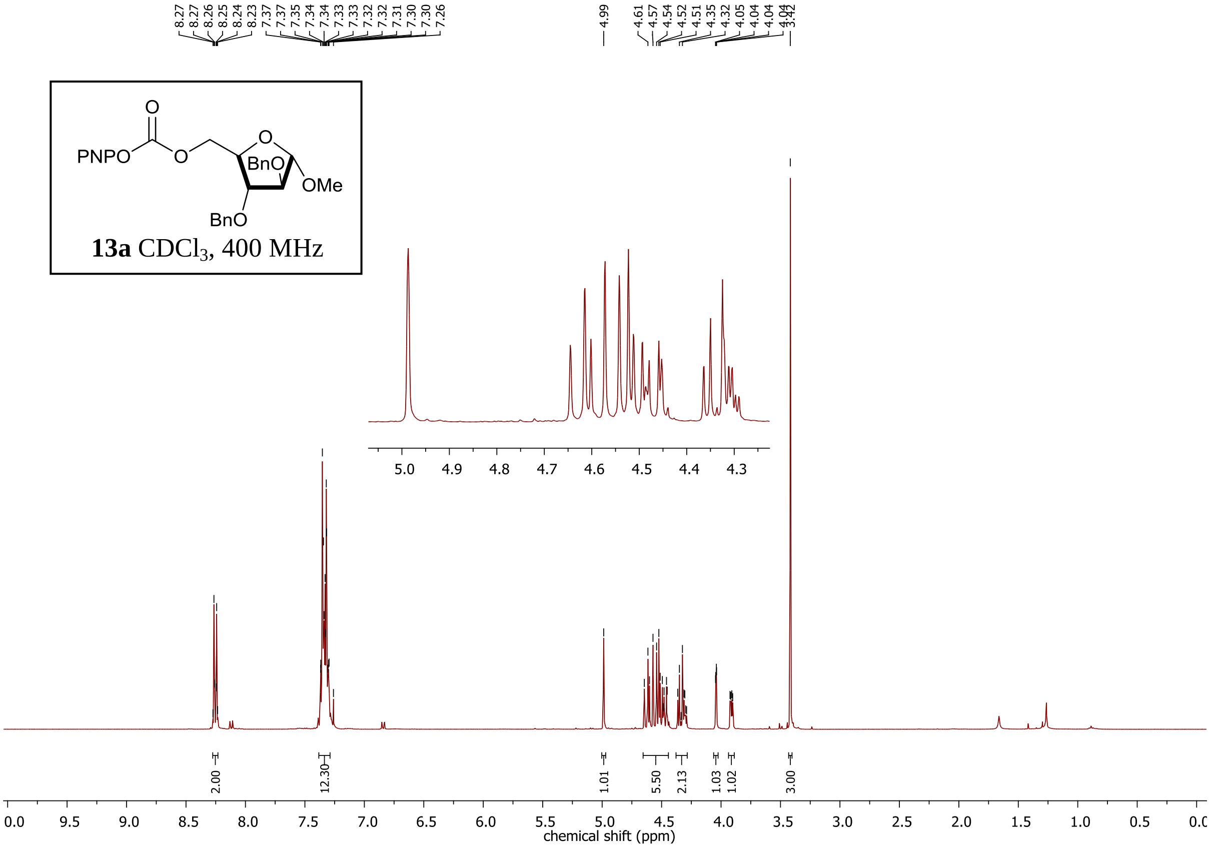
PNPO
O
BnO
OMe
BnO
13a CDCl3, 400 MHz
8.27 8.27 8.26 8.25 8.24 8.23 7.37 7.37 7.35 7.34 7.34 7.33 7.33 7.32 7.32 7.31 7.30 7.30 7.26
4.99
4.61 4.57 4.54 4.52 4.51 4.35 4.32 4.05 4.04 4.04 4.04 3.42
2.00
12.30
1.01
5.50
2.13
1.03
1.02
3.00
chemical shift (ppm)

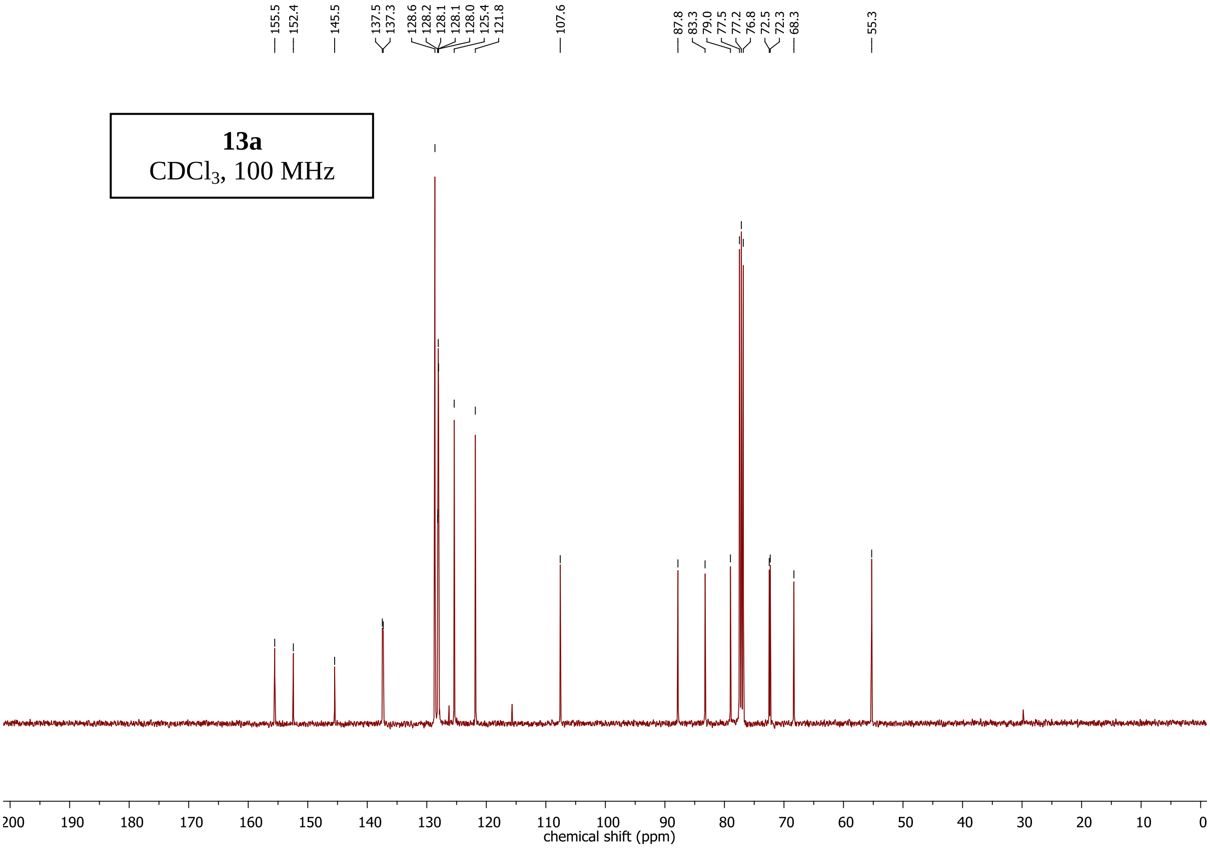
13a
CDCl3, 100 MHz
155.5 152.4 145.5 137.5 137.3 128.6 128.2 128.1 128.1 128.0 125.4 121.8 107.6
87.8 83.3 79.0 77.5 77.2 76.8 72.5 72.3 68.3 55.3
chemical shift (ppm)

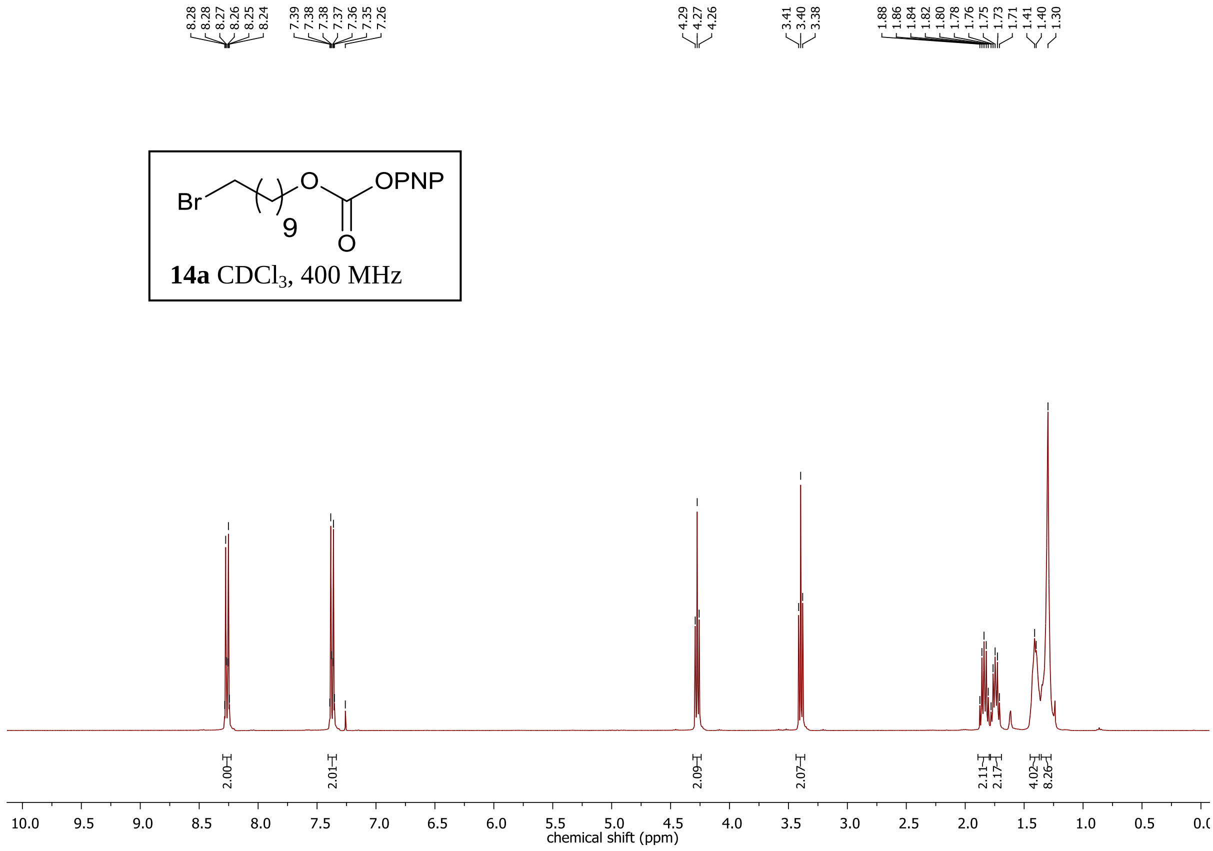
8.28 8.28 8.27 8.26 8.25 8.24
7.39 7.38 7.38 7.37 7.36 7.35 7.26
4.29 4.27 4.26
3.41 3.40 3.38
1.88 1.86 1.84 1.82 1.80 1.78 1.76 1.75 1.73 1.71 1.41 1.40 1.30
Br
O
OPNP
9
O
14a CDCl3, 400 MHz
2.00
2.01
2.09
2.07
2.11
2.17
4.02
8.26
10.0 9.5 9.0 8.5 8.0 7.5 7.0 6.5 6.0 5.5 5.0 4.5 4.0 3.5 3.0 2.5 2.0 1.5 1.0 0.5
chemical shift (ppm)

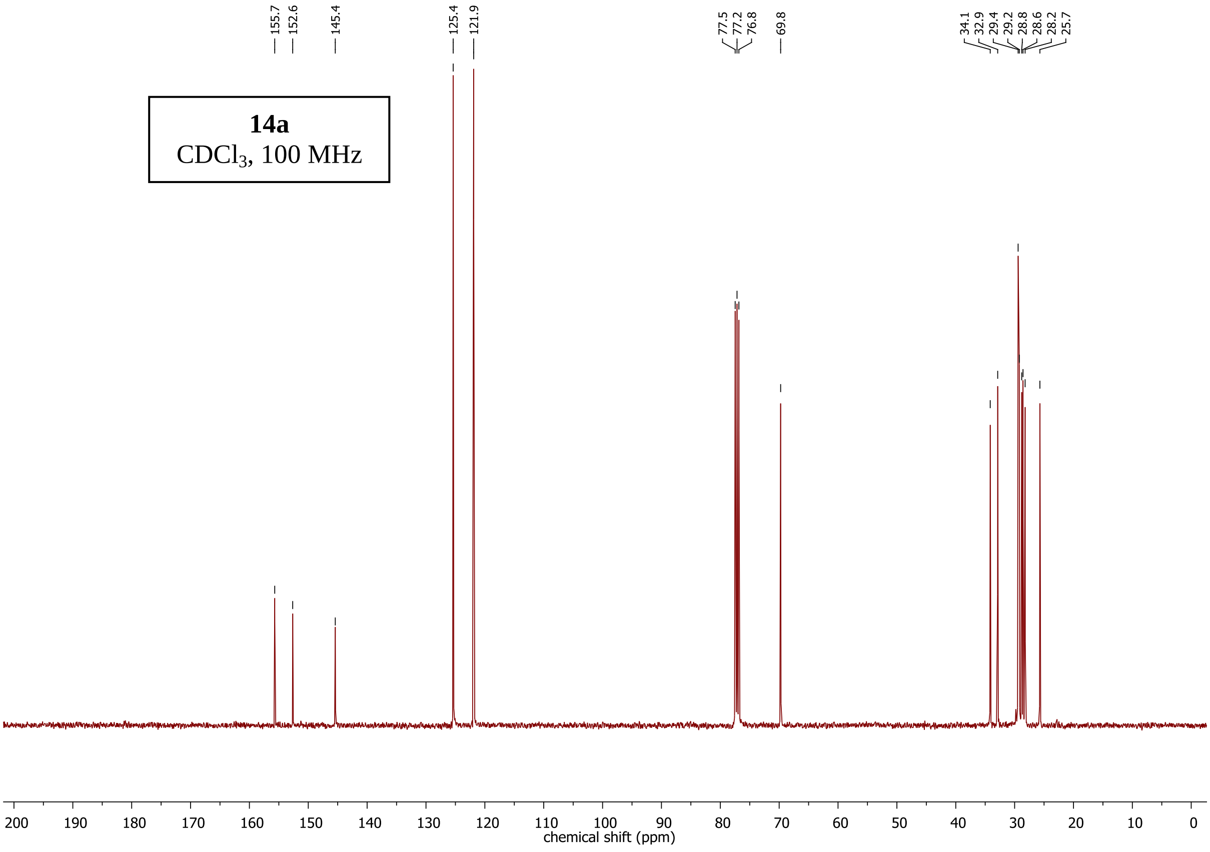
155.7 152.6 145.4 125.4 121.9 77.5 77.2 76.8 69.8 34.1 32.9 29.4 29.2 28.8 28.6 28.2 25.7
14a
CDCl3, 100 MHz
200 190 180 170 160 150 140 130 120 110 100 90 80 70 60 50 40 30 20 10 0
chemical shift (ppm)

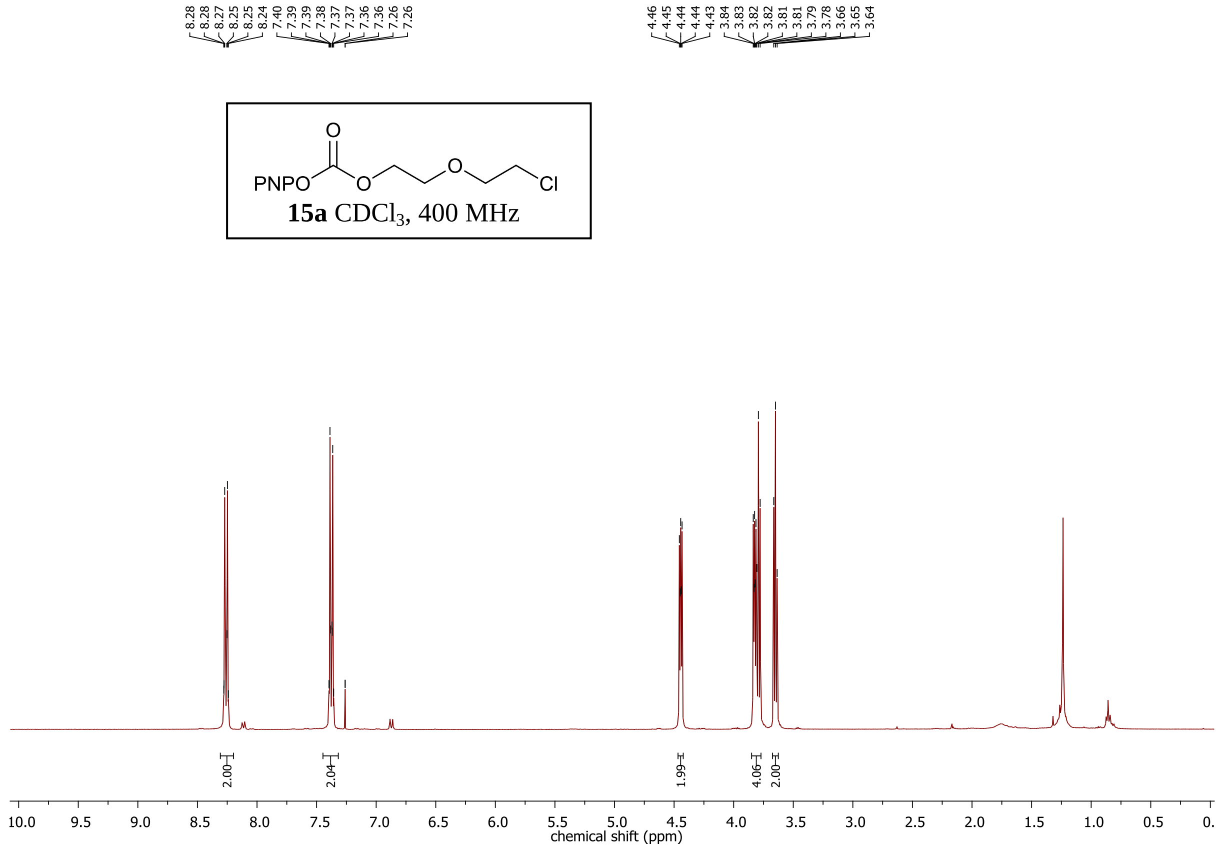

8.28
8.28
8.27
8.25
8.25
8.24
7.40
7.39
7.39
7.38
7.37
7.37
7.36
7.36
7.26
7.26
4.46
4.45
4.44
4.44
4.43
3.84
3.83
3.82
3.82
3.81
3.81
3.79
3.78
3.66
3.65
3.64
PNPO
O
O
O
Cl
15a CDCl3, 400 MHz
2.00
2.04
1.99
4.06
2.00
chemical shift (ppm)

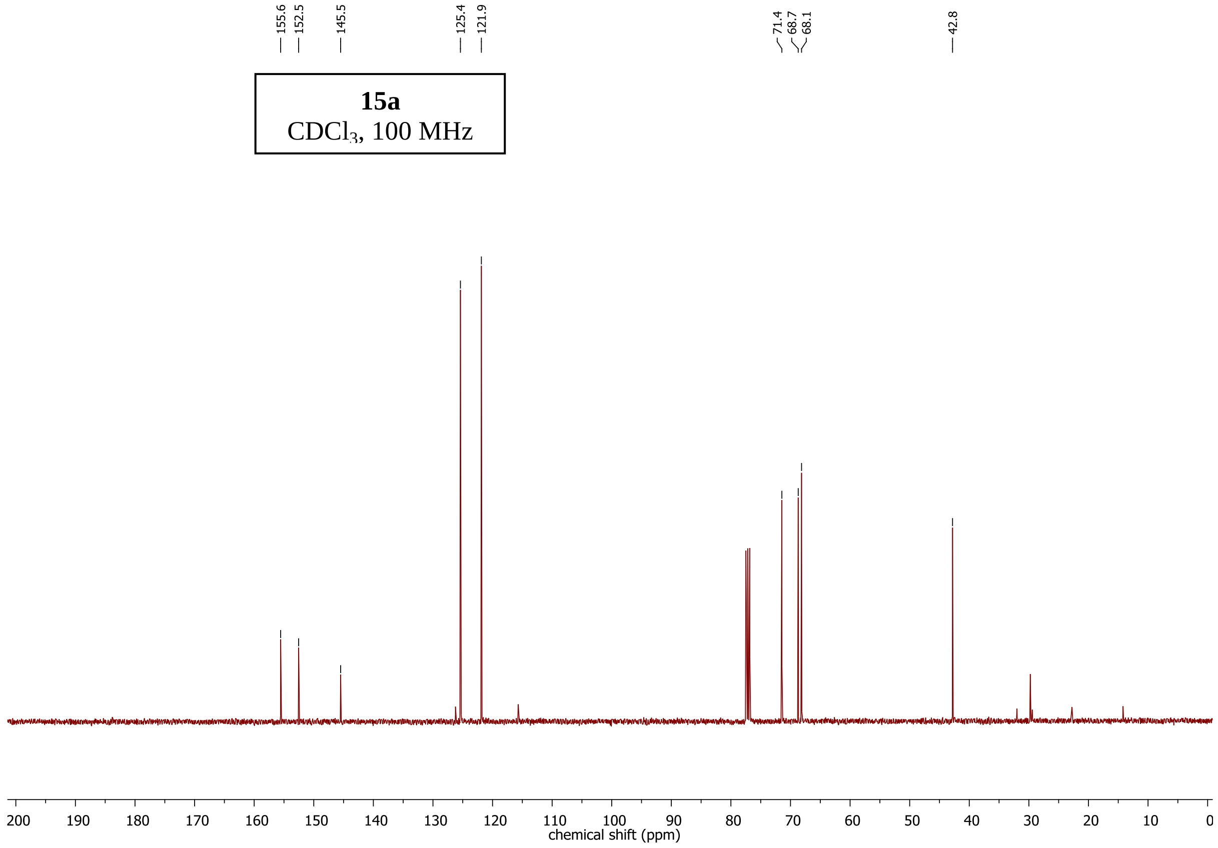

155.6
152.5
145.5
125.4
121.9
71.4
68.7
68.1
42.8
15a
CDCl3, 100 MHz
chemical shift (ppm)

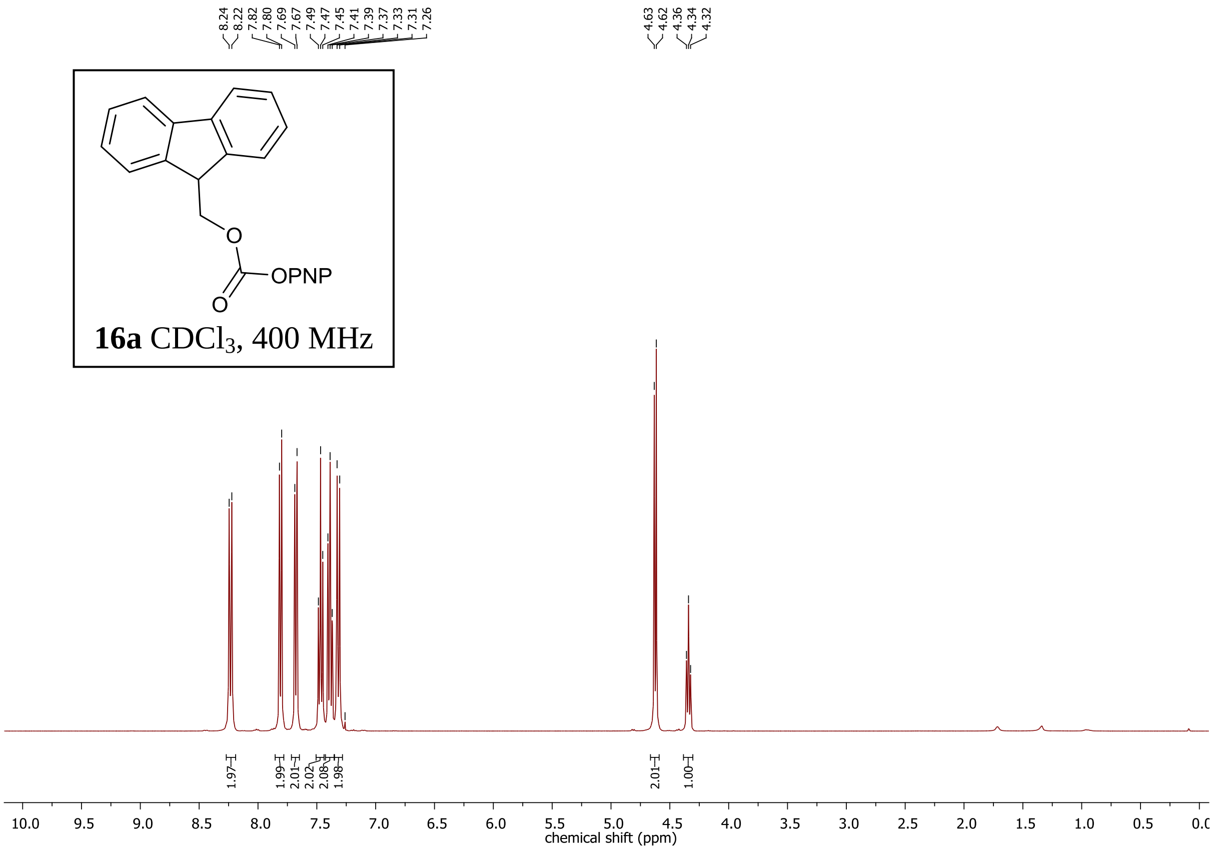
OPNP
16a CDCl3, 400 MHz
chemical shift (ppm)

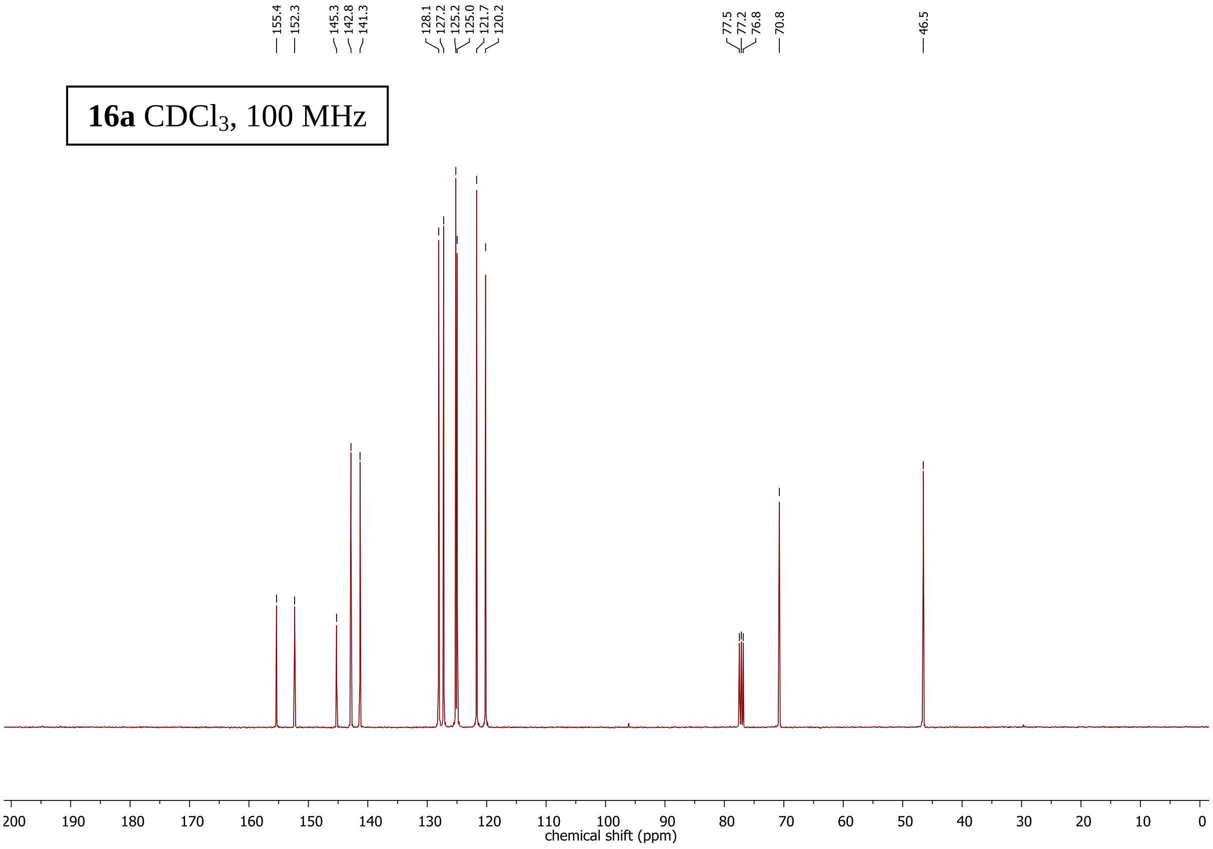
16a CDCl3, 100 MHz
chemical shift (ppm)

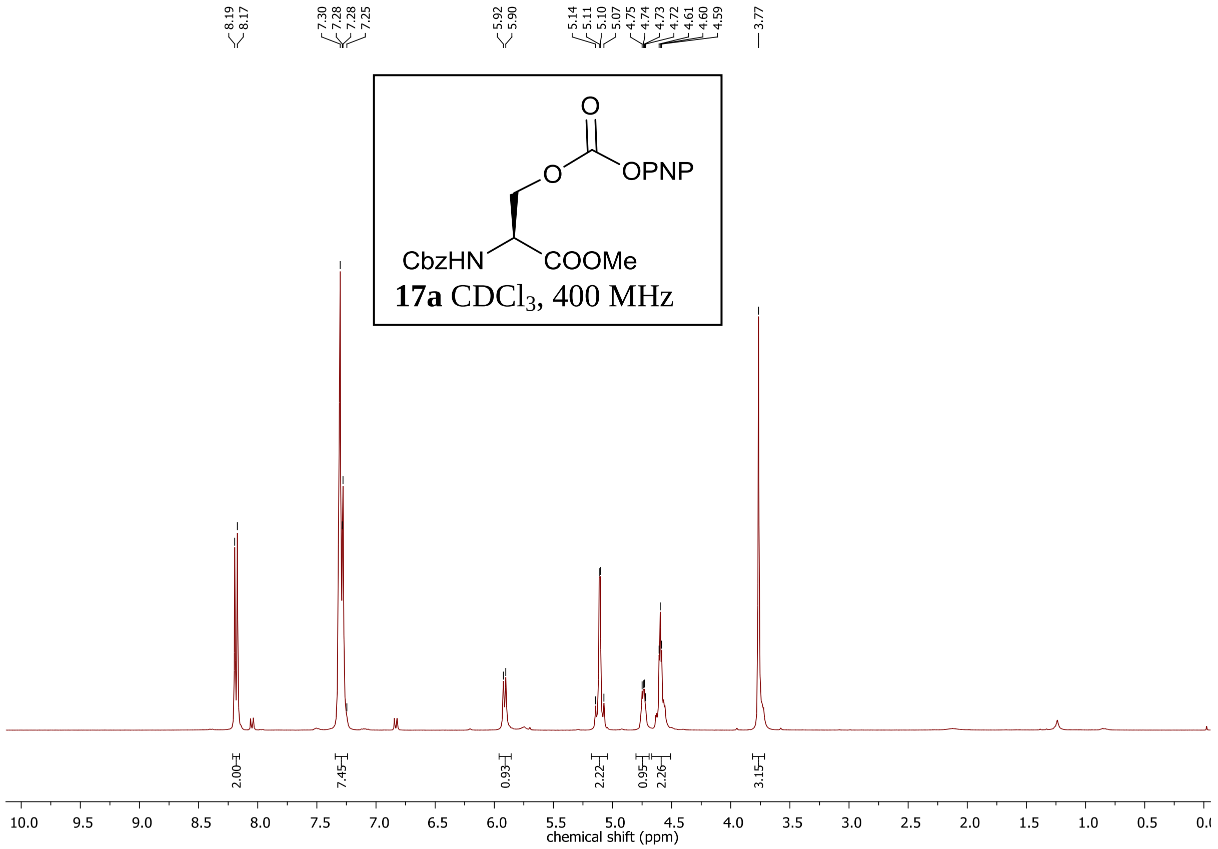
8.19
8.17
7.30
7.28
7.28
7.25
5.92
5.90
5.14
5.11
5.10
5.07
4.75
4.74
4.73
4.72
4.61
4.60
4.59
3.77
O
OPNP
O
CbzHN
COOMe
17a CDCl3, 400 MHz
2.00
7.45
0.93
2.22
0.95
2.26
3.15
chemical shift (ppm)

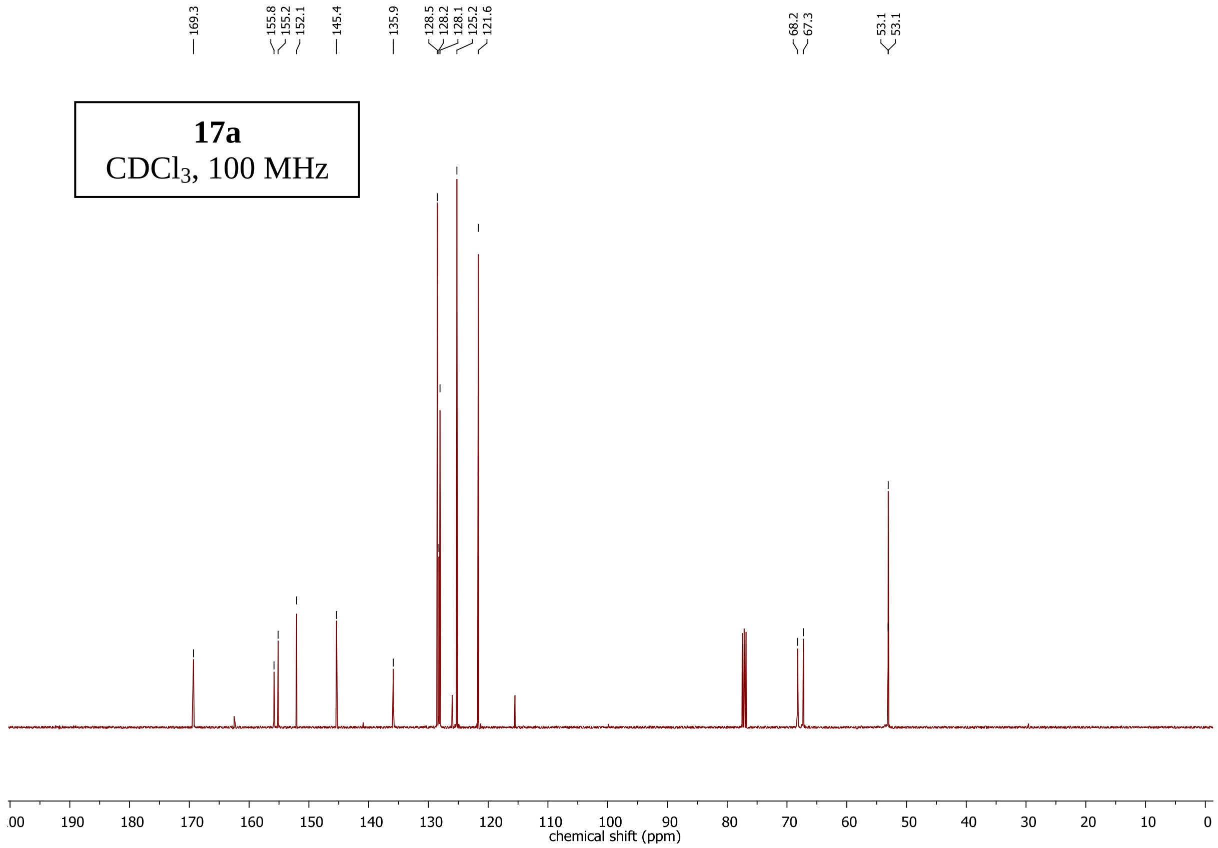
169.3
155.8
155.2
152.1
145.4
135.9
128.5
128.2
128.1
125.2
121.6
68.2
67.3
53.1
53.1
17a
CDCl3, 100 MHz
chemical shift (ppm)

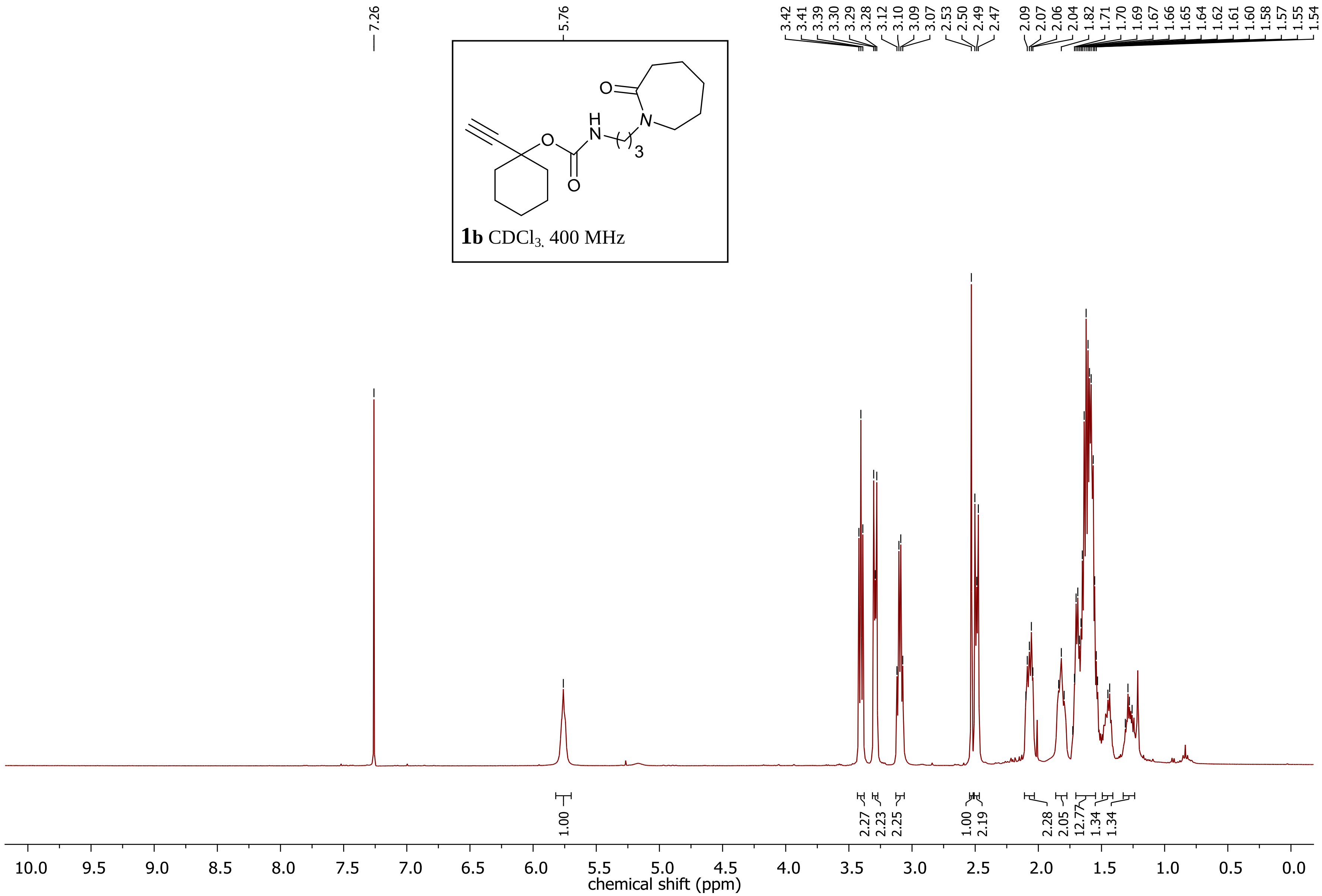
7.26
5.76
3.42 3.41 3.39 3.30 3.29 3.28 3.12 3.10 3.09 3.07 2.53 2.50 2.49 2.47
2.09 2.07 2.06 2.04 1.82 1.71 1.70 1.69 1.67 1.66 1.65 1.64 1.62 1.61 1.60 1.58 1.57 1.55 1.54
1b CDCl3, 400 MHz
1.00
2.27 2.23 2.25
1.00 2.19
2.28 2.05 12.77 1.34 1.34
10.0 9.5 9.0 8.5 8.0 7.5 7.0 6.5 6.0 5.5 5.0 4.5 4.0 3.5 3.0 2.5 2.0 1.5 1.0 0.5 0.0
chemical shift (ppm)

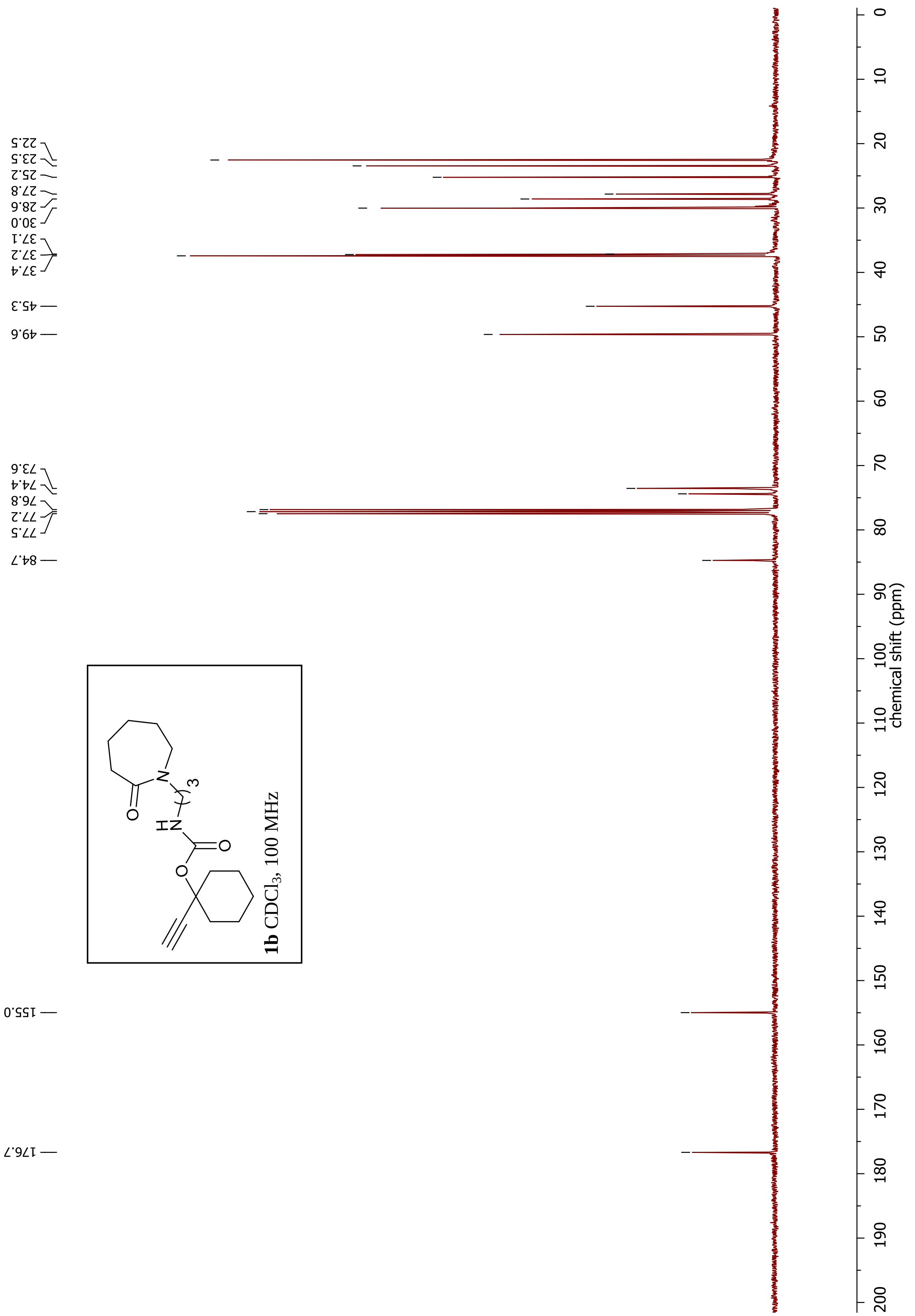
22.5
23.5
25.2
27.8
28.6
30.0
37.1
37.2
37.4
45.3
49.6
73.6
74.4
76.8
77.2
77.5
84.7
155.0
176.7
1b CDCl3, 100 MHz
0
10
20
30
40
50
60
70
80
90
100
110
120
130
140
150
160
170
180
190
200
chemical shift (ppm)

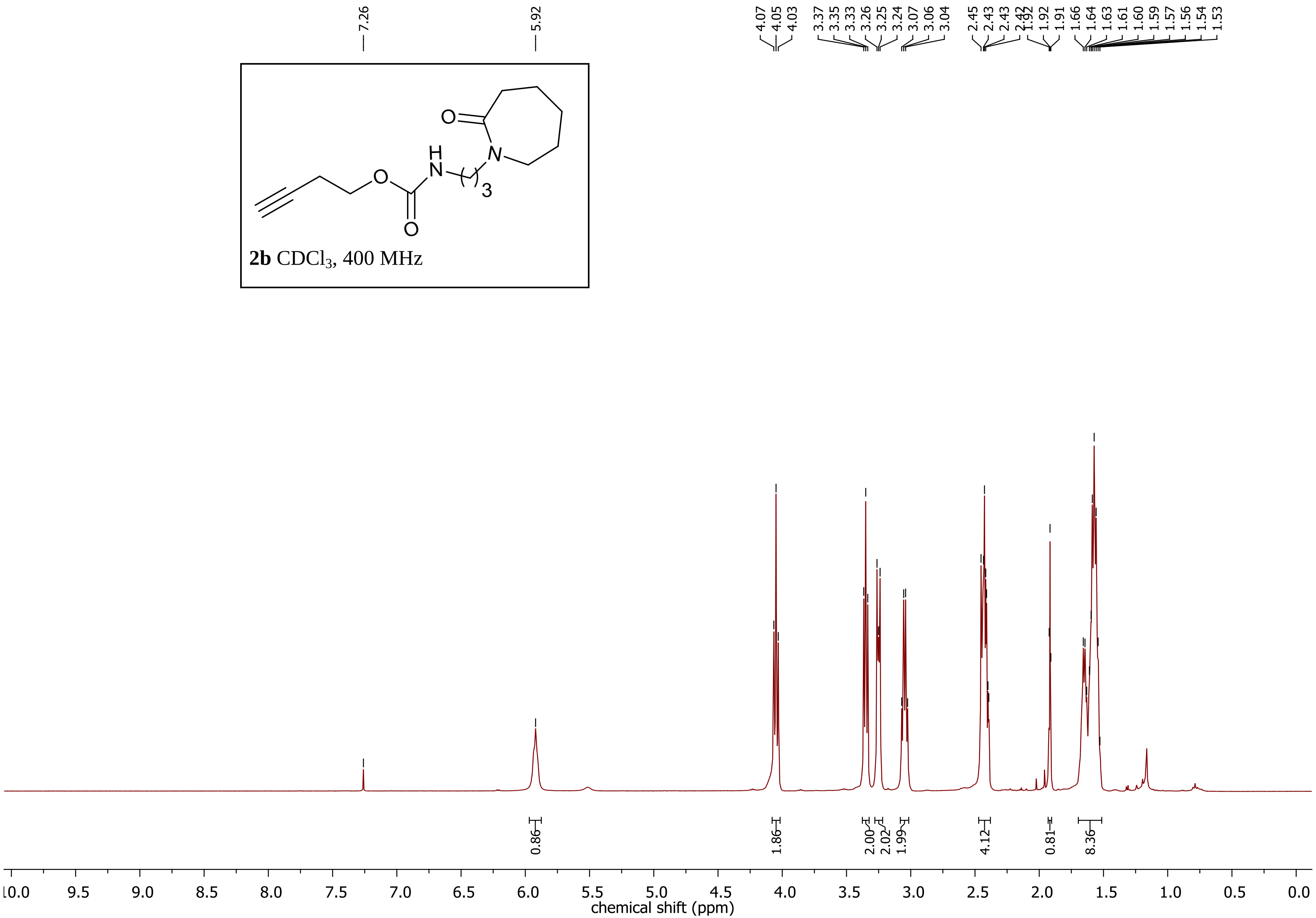

2b $CDCl_3$, 400 MHz

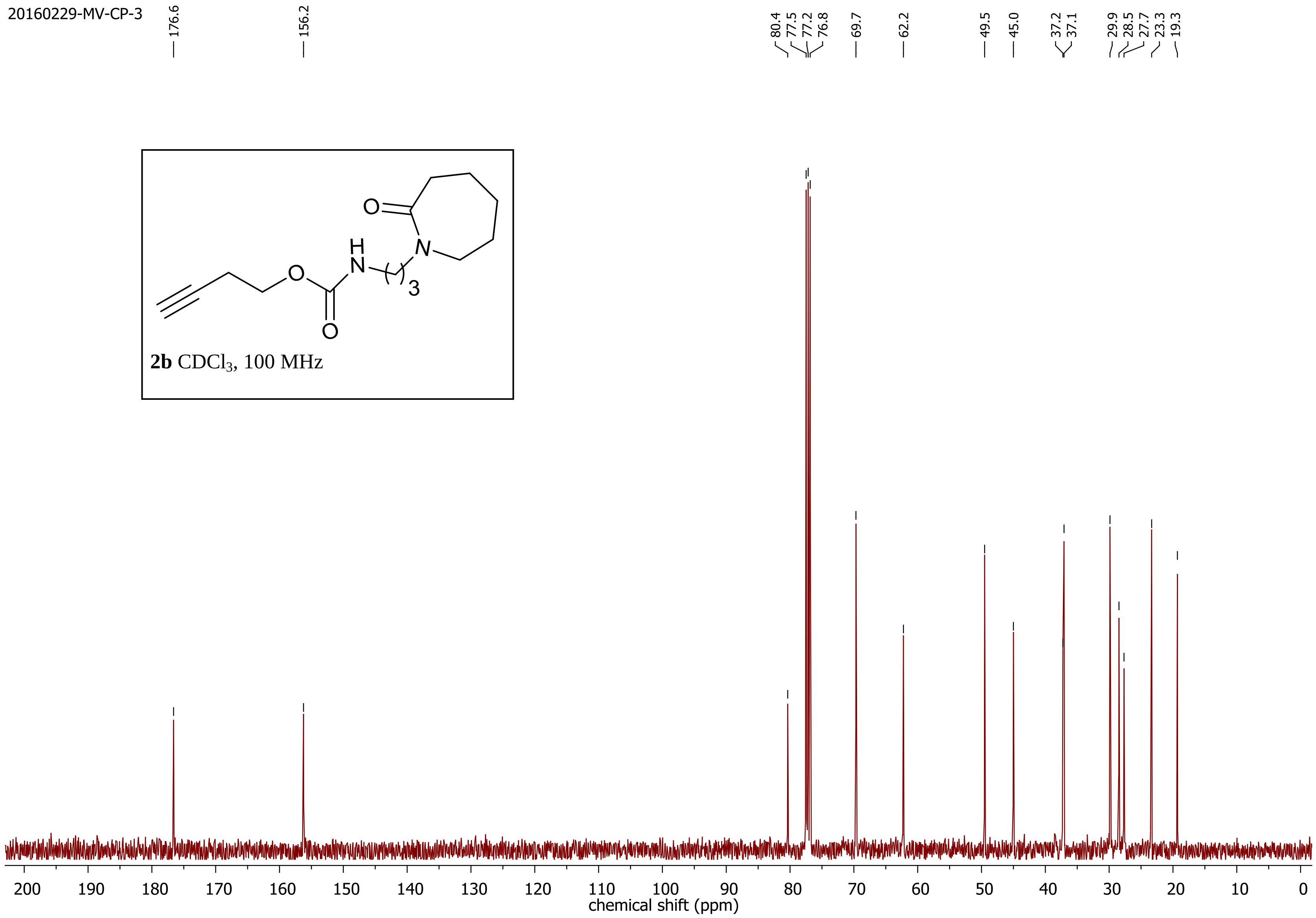

20160229-MV-CP-3
176.6
156.2
80.4
77.5
77.2
76.8
69.7
62.2
49.5
45.0
37.2
37.1
29.9
28.5
27.7
23.3
19.3
O
N
H
N
3
O
O
2b CDCl3, 100 MHz
200
190
180
170
160
150
140
130
120
110
100
90
80
70
60
50
40
30
20
10
0
chemical shift (ppm)

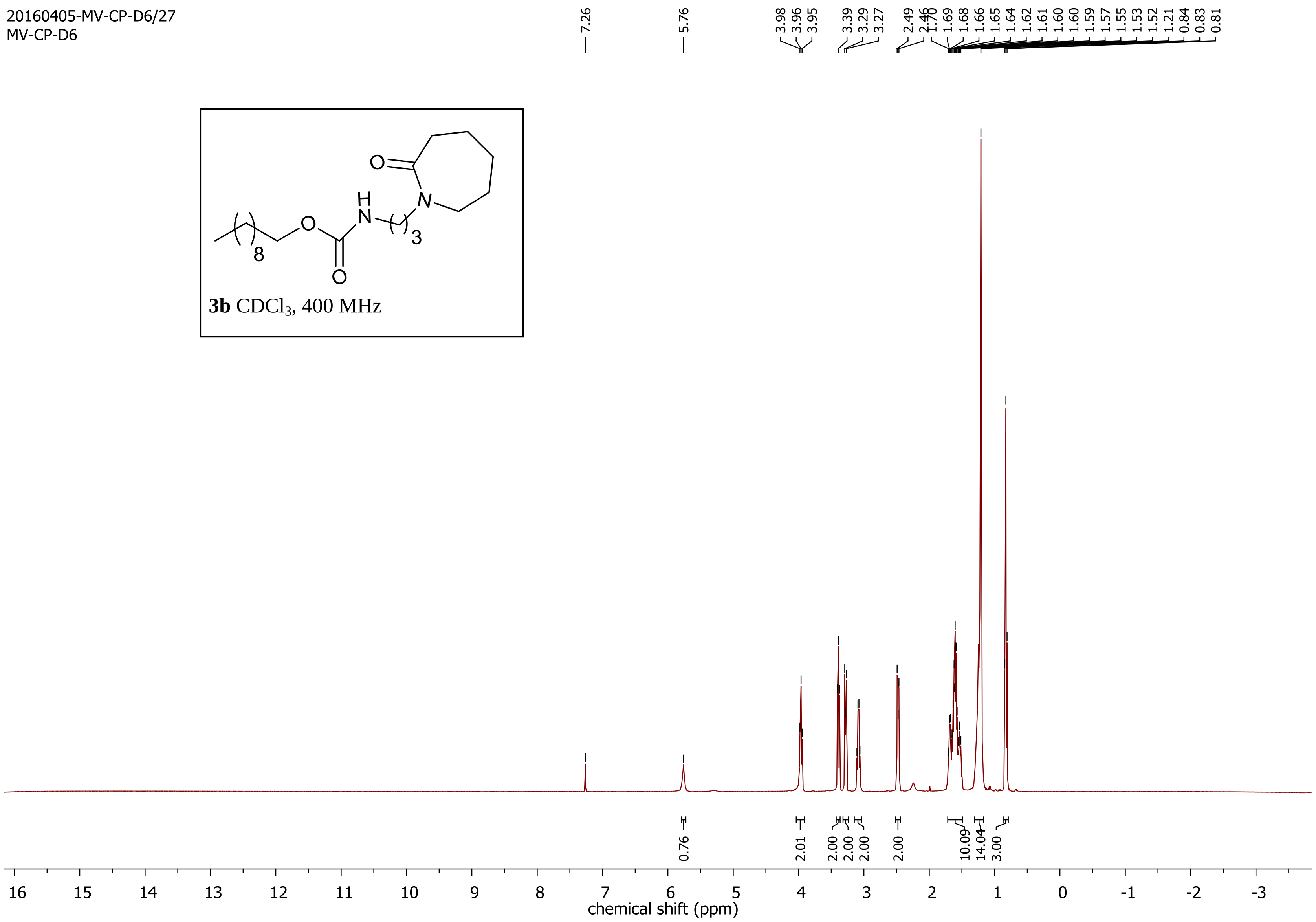

20160405-MV-CP-D6/27
MV-CP-D6
7.26
5.76
3.98
3.96
3.95
3.39
3.29
3.27
2.49
2.46
1.70
1.69
1.68
1.66
1.65
1.64
1.62
1.61
1.60
1.60
1.59
1.57
1.55
1.53
1.52
1.21
0.84
0.83
0.81
O
H
N
N
O
O
8
3
3b $CDCl_3$, 400 MHz
0.76
2.01
2.00
2.00
2.00
2.00
10.09
14.04
3.00
16 15 14 13 12 11 10 9 8 7 6 5 4 3 2 1 0 -1 -2 -3
chemical shift (ppm)

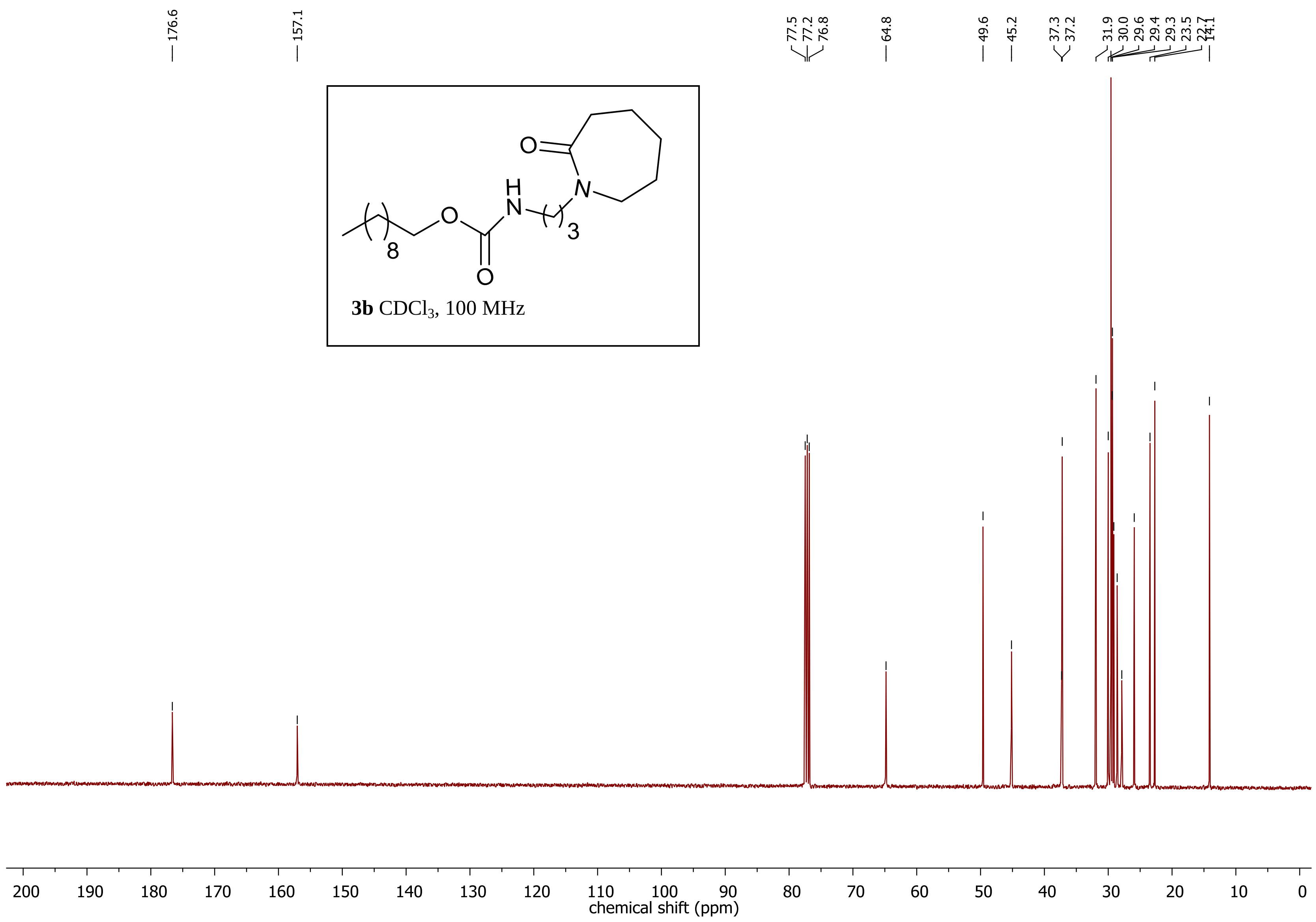
176.6
157.1
77.5
77.2
76.8
64.8
49.6
45.2
37.3
37.2
31.9
30.0
29.6
29.4
29.3
23.5
22.7
14.1
3b CDCl3, 100 MHz
chemical shift (ppm)

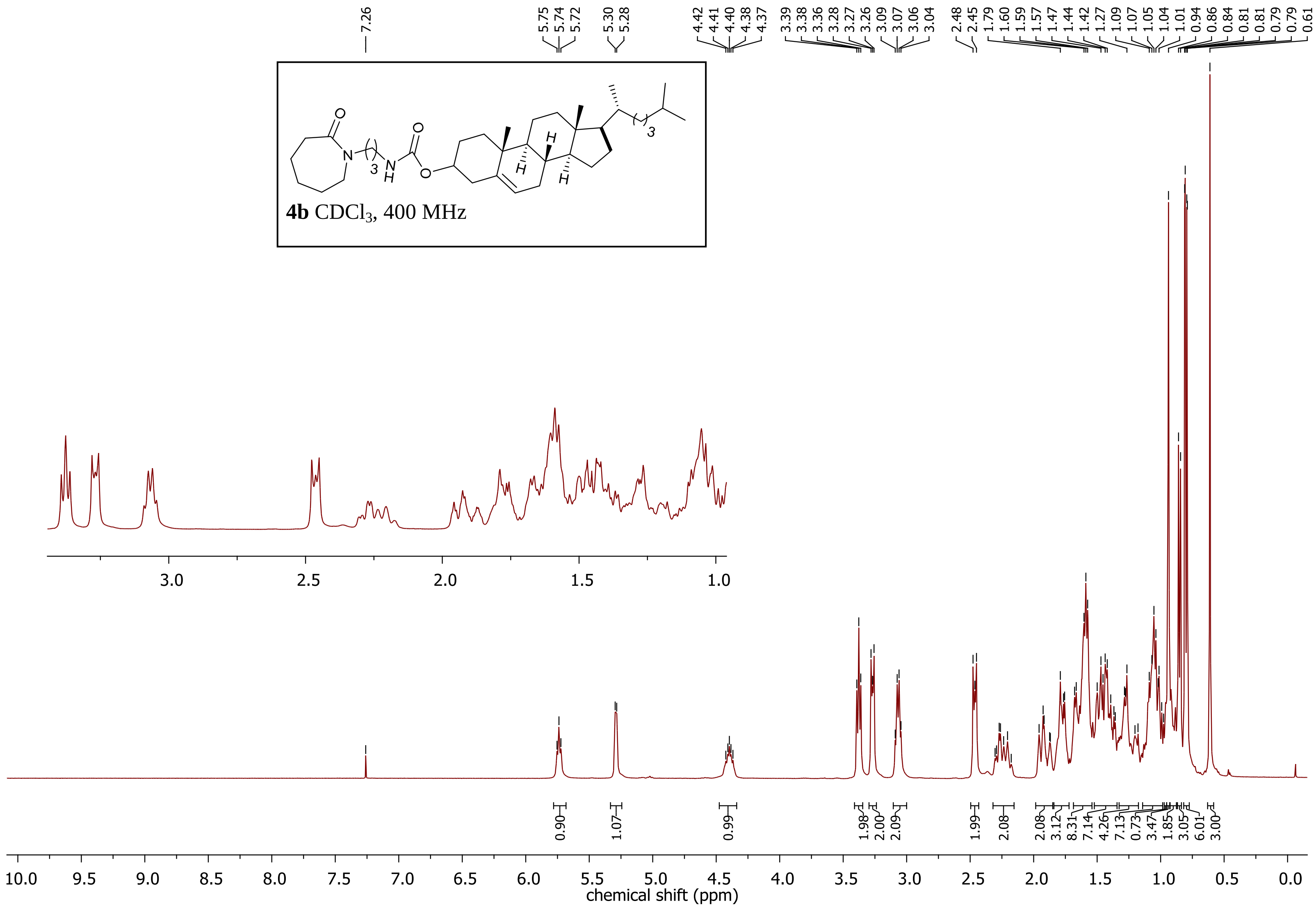

4b $CDCl_3$, 400 MHz

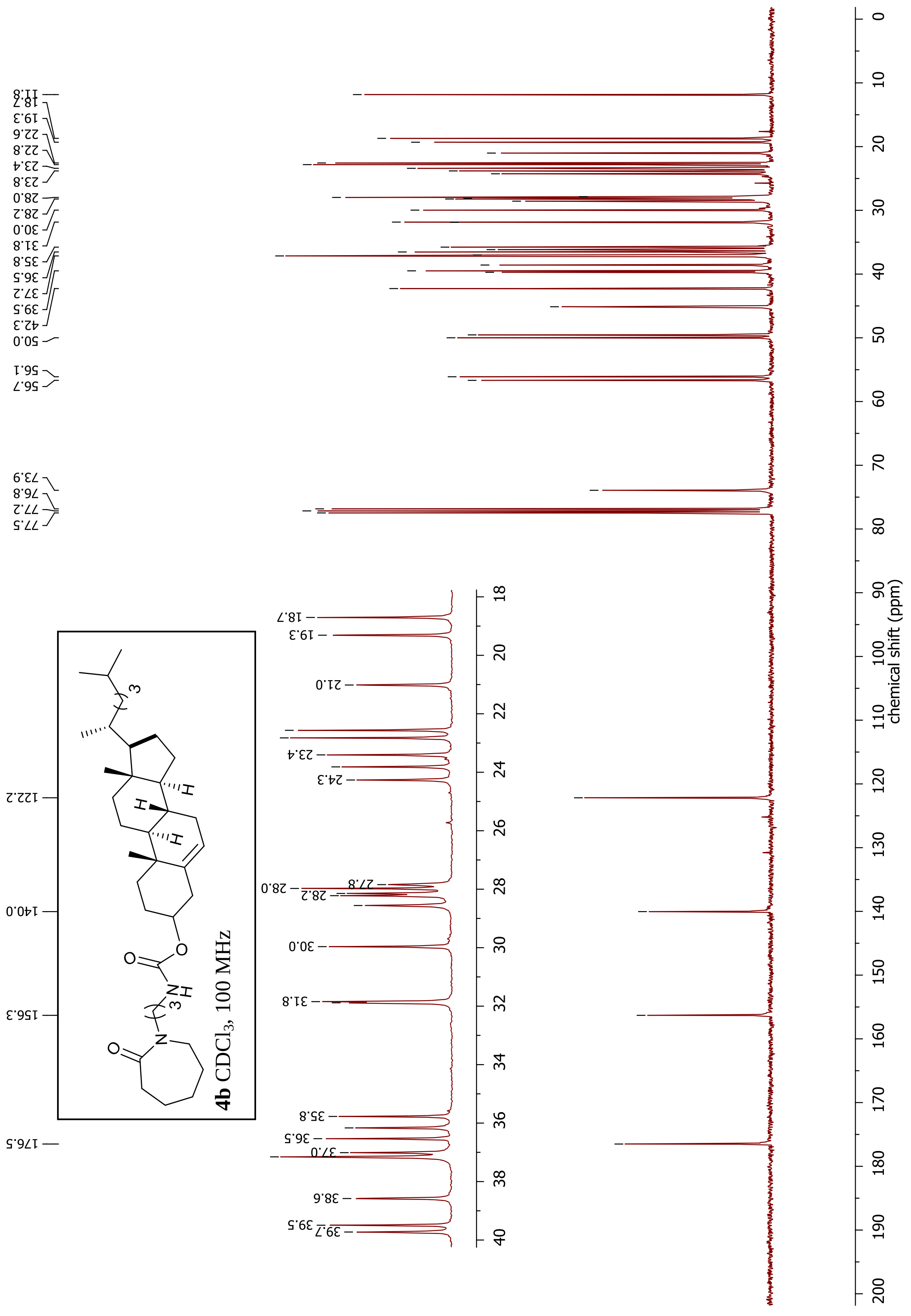
4b CDCl$_3$, 100 MHz
176.5
156.3
140.0
122.2
77.5
77.2
76.8
73.9
56.7
56.1
50.0
42.3
39.5
37.2
36.5
35.8
31.8
30.0
28.2
28.0
23.8
23.4
22.8
22.6
19.3
18.7
11.8
39.7
39.5
38.6
37.0
36.5
35.8
31.8
30.0
28.2
28.0
27.8
24.3
23.4
21.0
19.3
18.7
chemical shift (ppm)

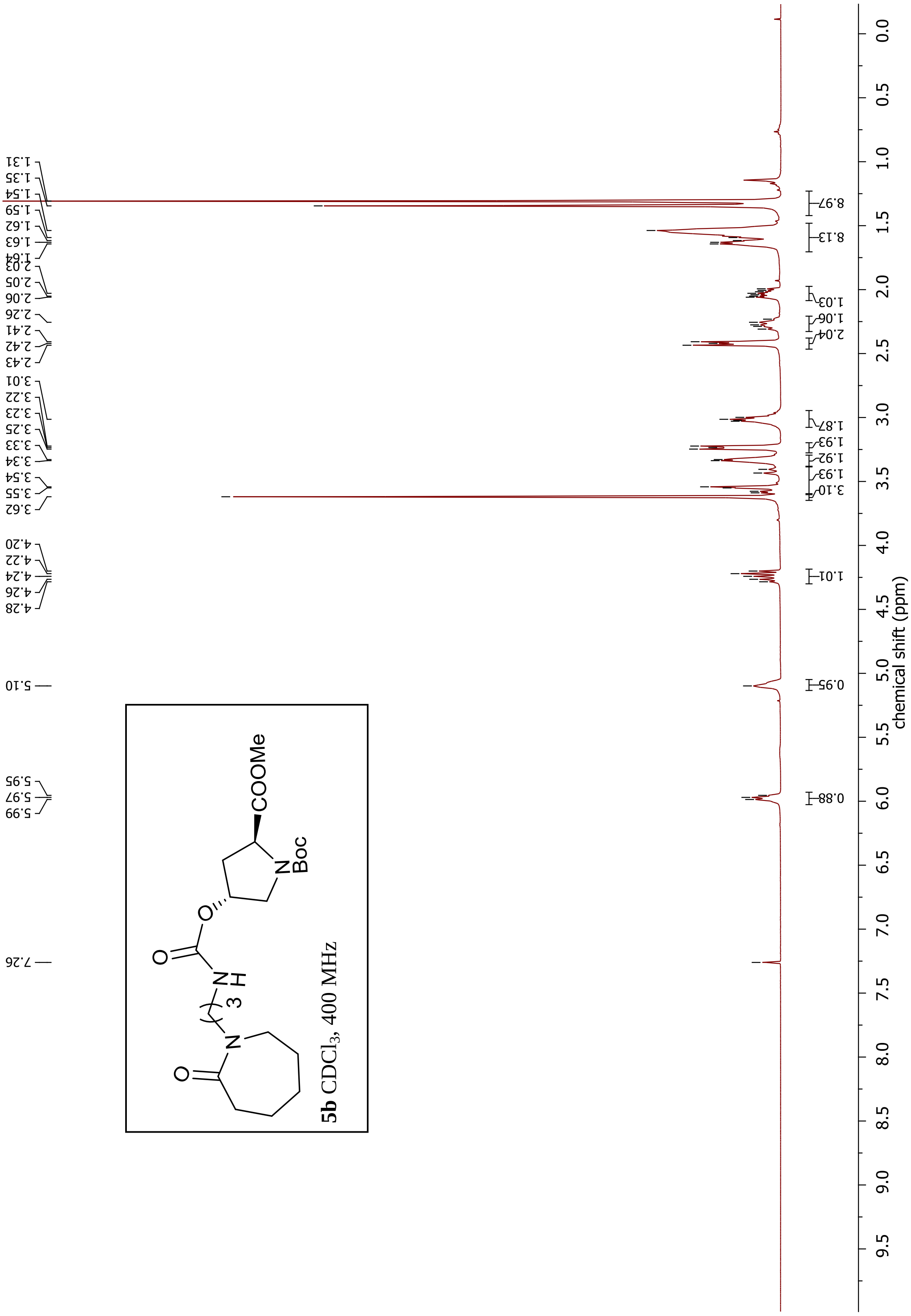
5b CDCl$_3$, 400 MHz
COOMe
Boc
chemical shift (ppm)

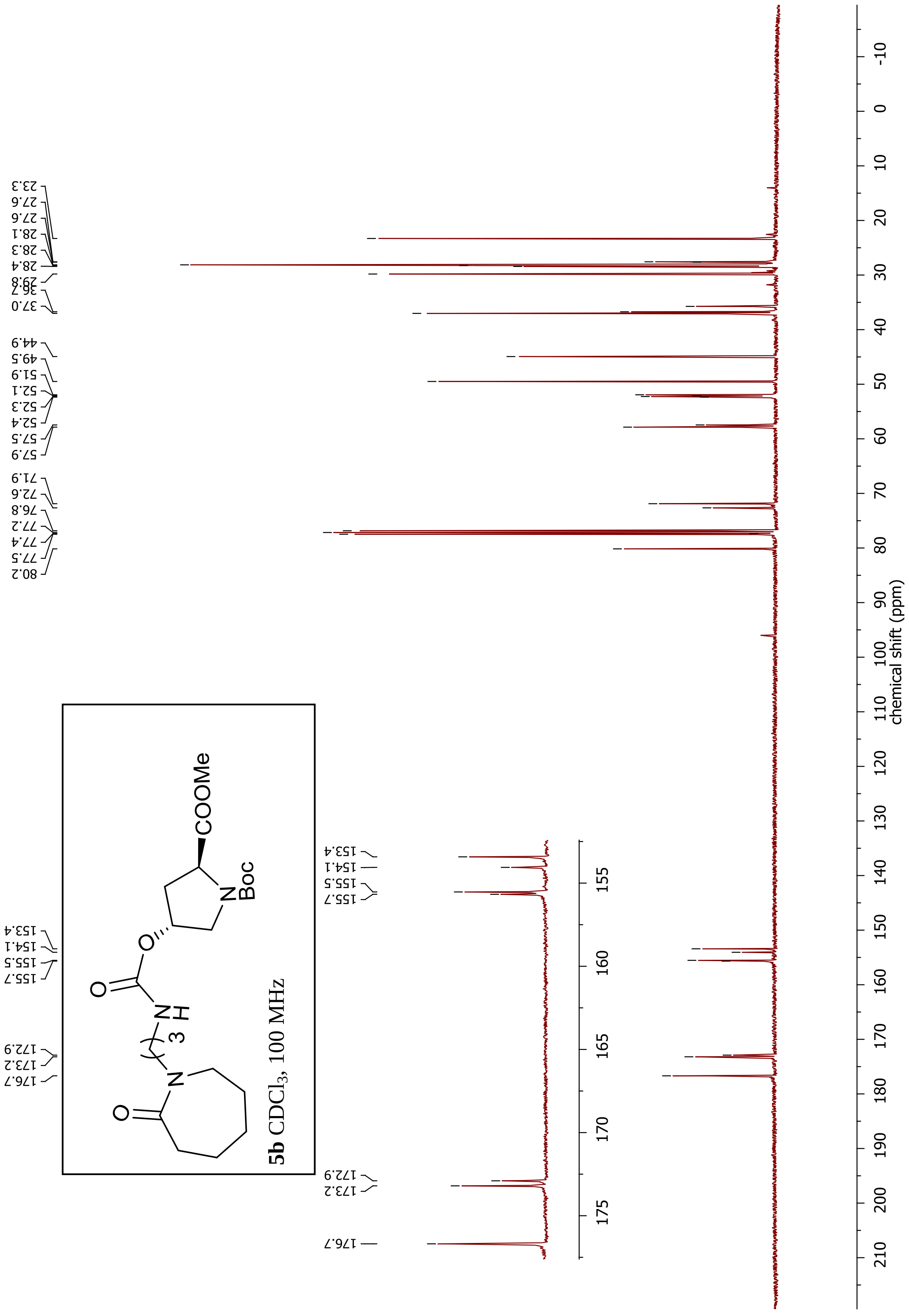
5b CDCl3, 100 MHz
COOMe
Boc
176.7
173.2
172.9
155.7
155.5
154.1
153.4
80.2
77.5
77.4
77.2
76.8
72.6
71.9
57.9
57.5
52.4
52.3
52.1
51.9
49.5
44.9
37.0
36.7
29.8
28.4
28.3
28.1
27.6
27.6
23.3
chemical shift (ppm)

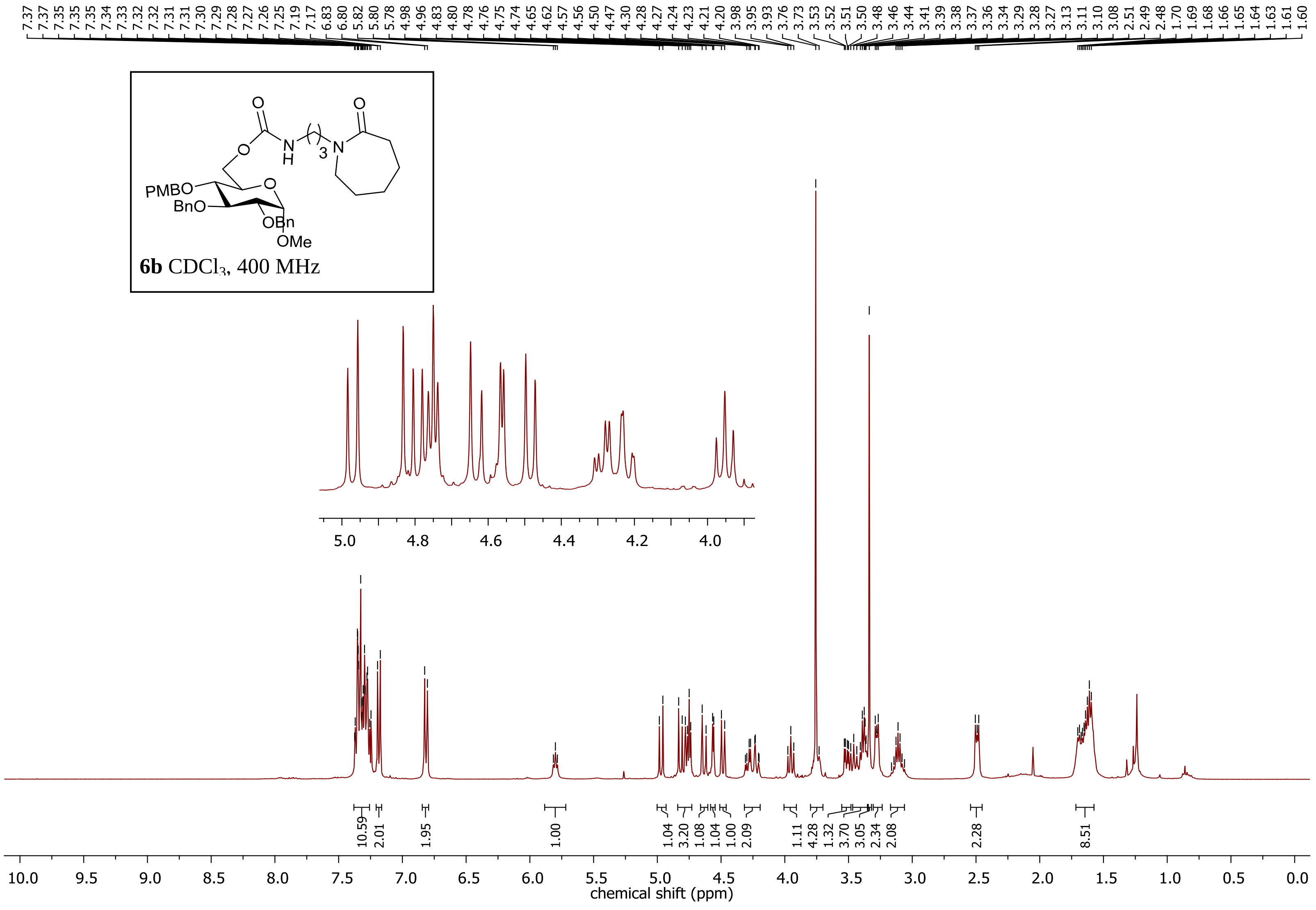

6b $CDCl_3$, 400 MHz

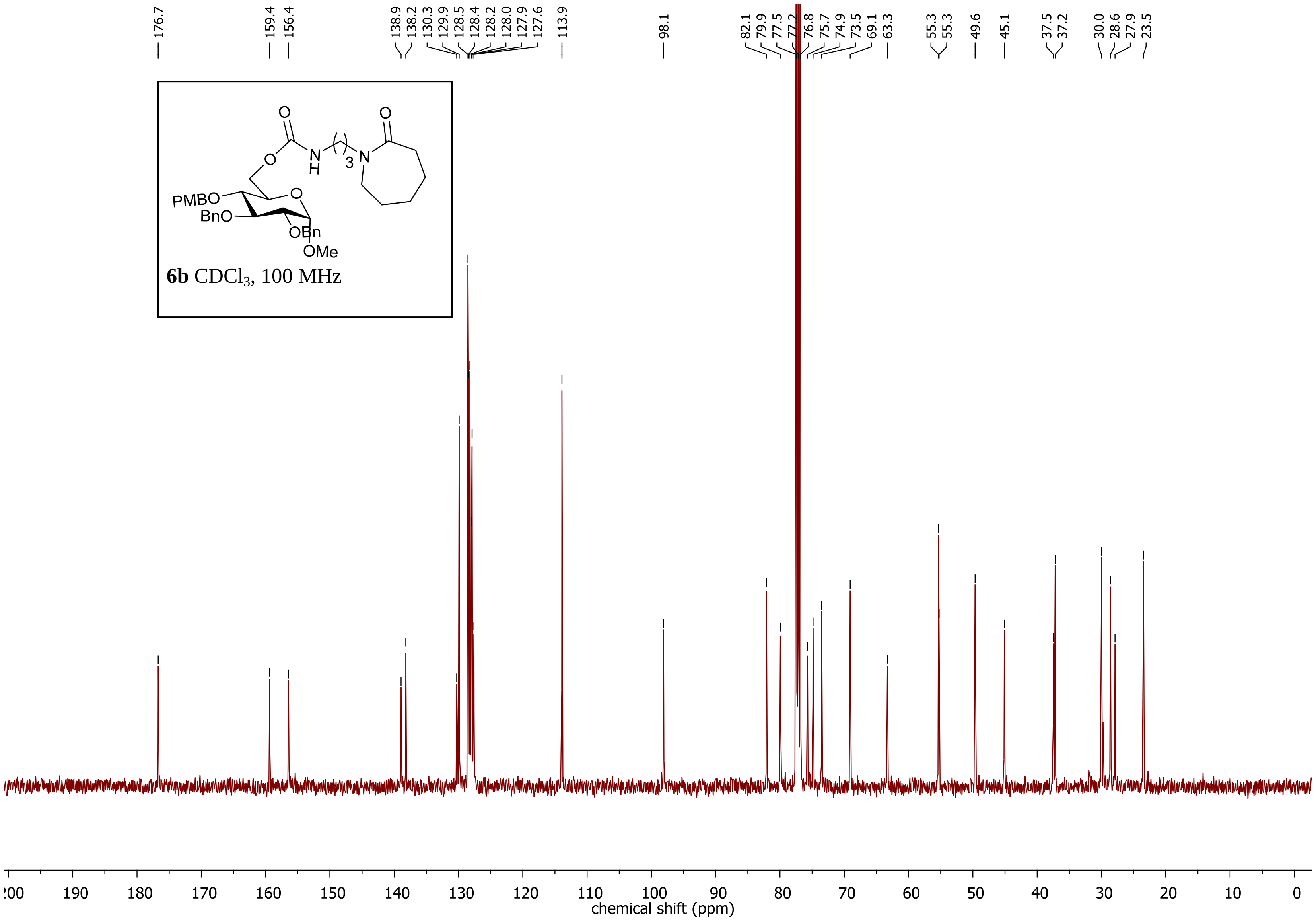
176.7
159.4
156.4
138.9
138.2
130.3
129.9
128.5
128.4
128.2
128.0
127.9
127.6
113.9
98.1
82.1
79.9
77.5
77.2
76.8
75.7
74.9
73.5
69.1
63.3
55.3
55.3
49.6
45.1
37.5
37.2
30.0
28.6
27.9
23.5
PMBO
BnO
OBn
OMe
O
N
H
3
6b CDCl3, 100 MHz
chemical shift (ppm)

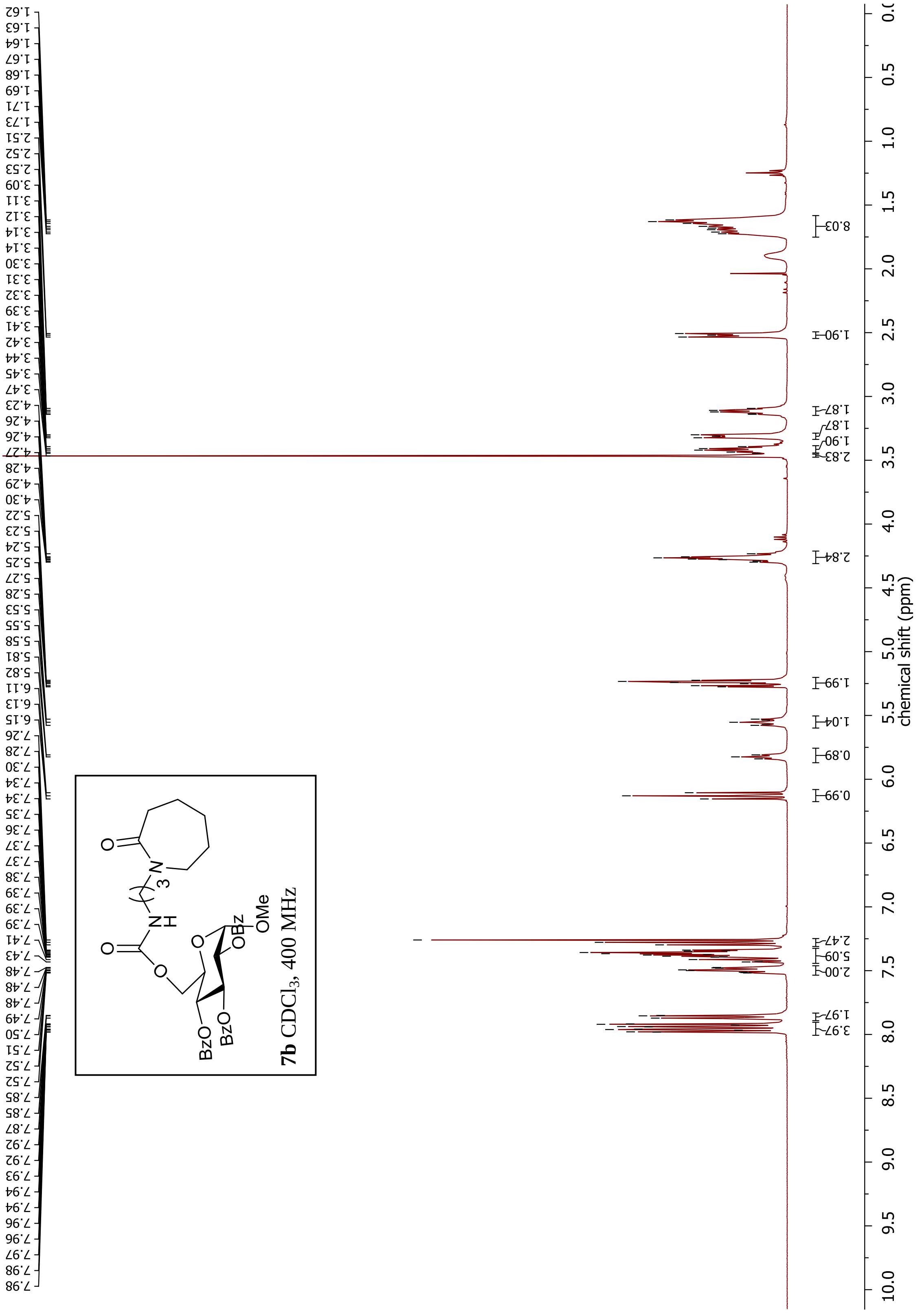
7b CDCl3, 400 MHz
chemical shift (ppm)

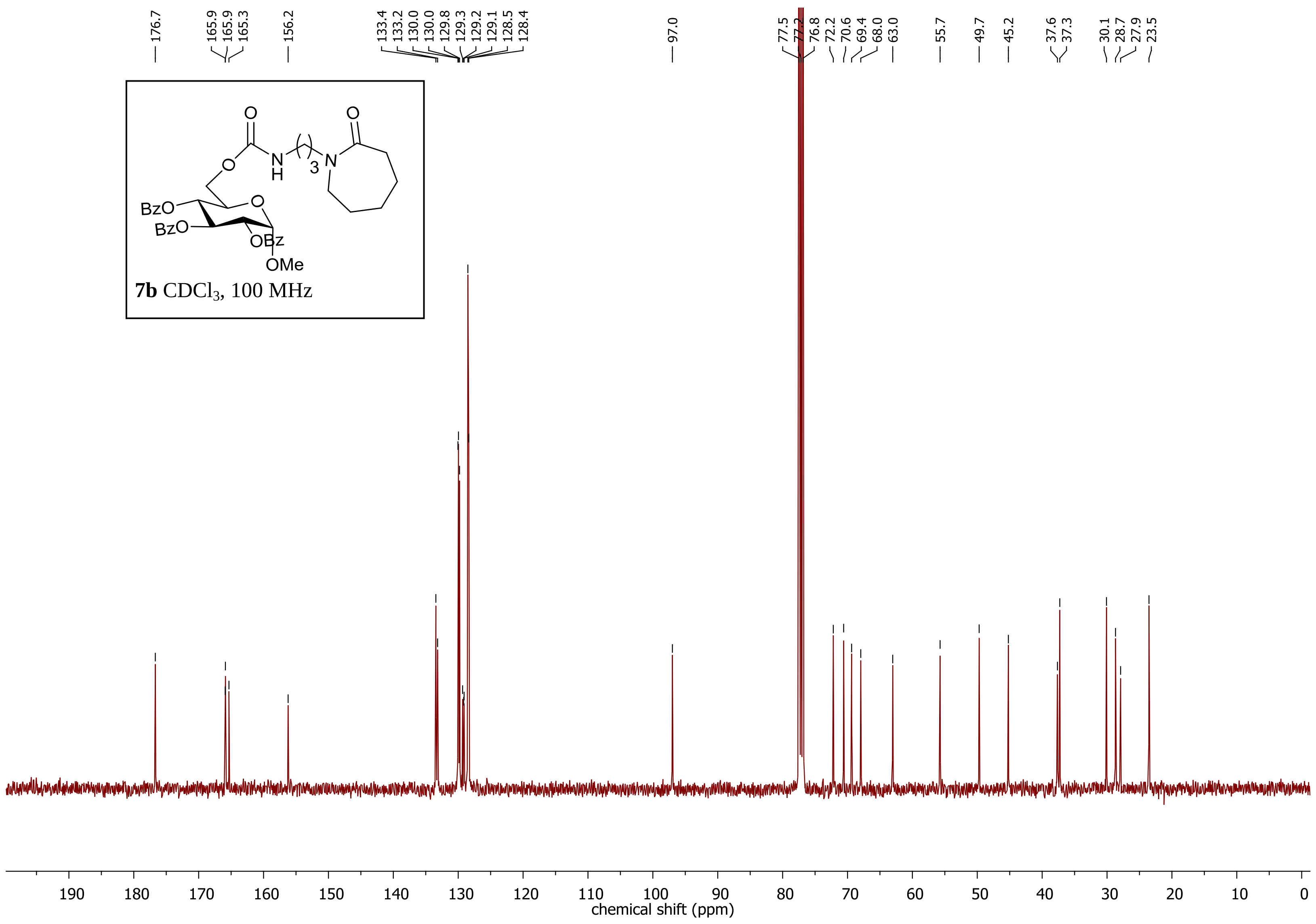
176.7
165.9
165.9
165.3
156.2
133.4
133.2
130.0
130.0
129.8
129.3
129.2
129.1
128.5
128.4
97.0
77.5
77.2
76.8
72.2
70.6
69.4
68.0
63.0
55.7
49.7
45.2
37.6
37.3
30.1
28.7
27.9
23.5
BzO
BzO
OBz
OMe
O
O
N
H
3
N
O
7b CDCl3, 100 MHz
190
180
170
160
150
140
130
120
110
100
90
80
70
60
50
40
30
20
10
0
chemical shift (ppm)

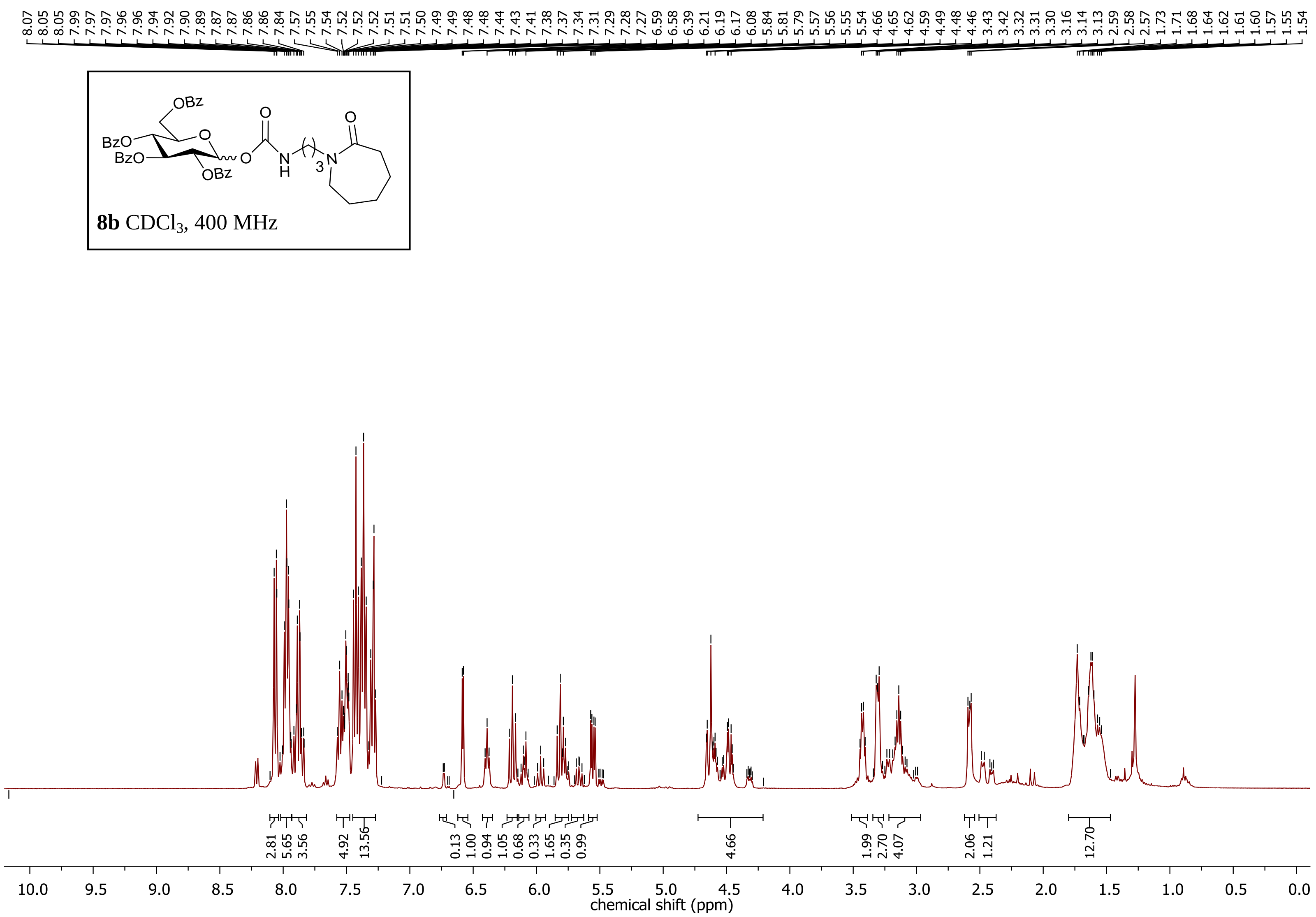

8b $CDCl_3$, 400 MHz

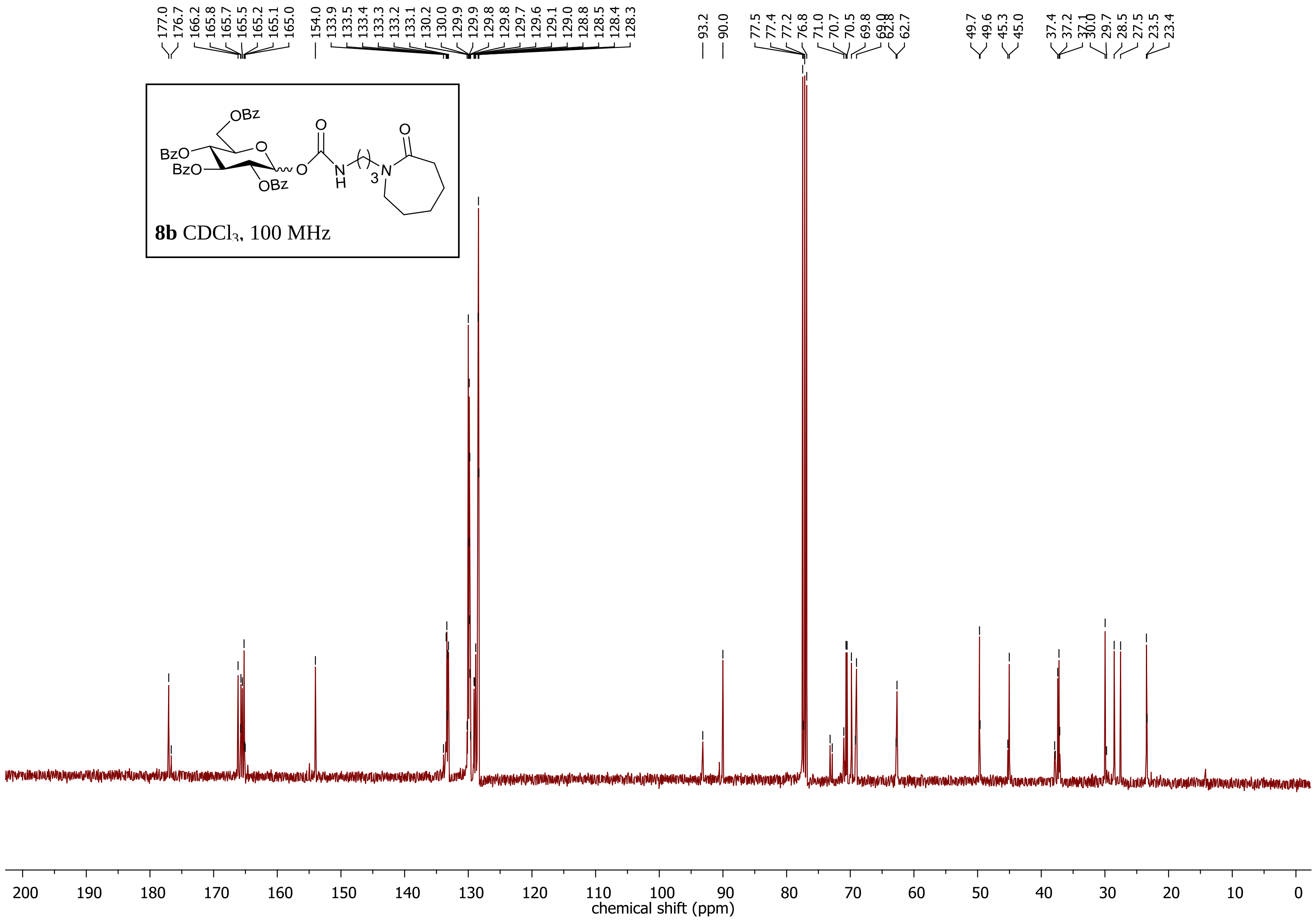

8b $CDCl_3$, 100 MHz

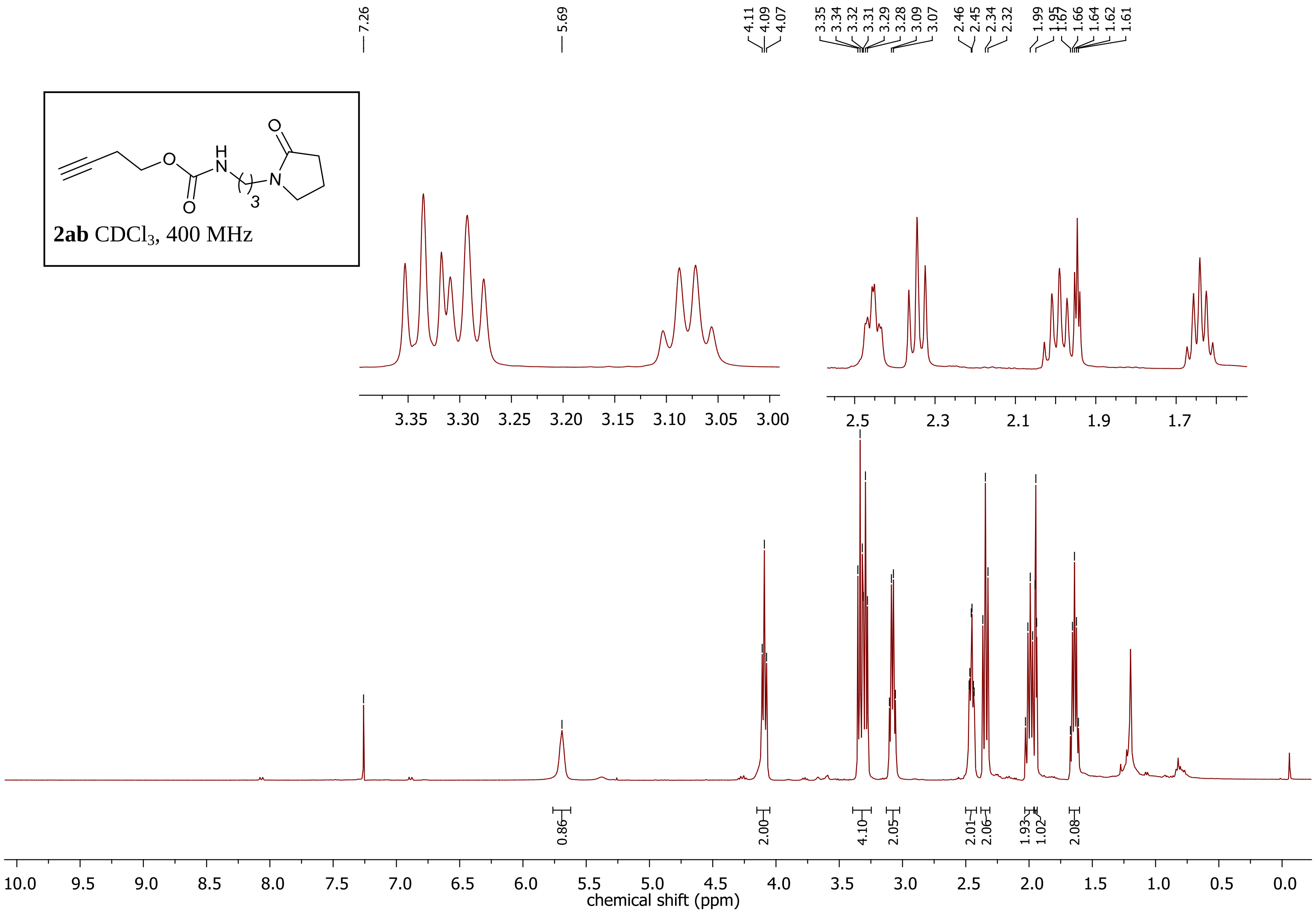

2ab CDCl3, 400 MHz
7.26
5.69
4.11
4.09
4.07
3.35
3.34
3.32
3.31
3.29
3.28
3.09
3.07
2.46
2.45
2.34
2.32
1.99
1.95
1.67
1.66
1.64
1.62
1.61
0.86
2.00
4.10
2.05
2.01
2.06
1.93
1.02
2.08
chemical shift (ppm)

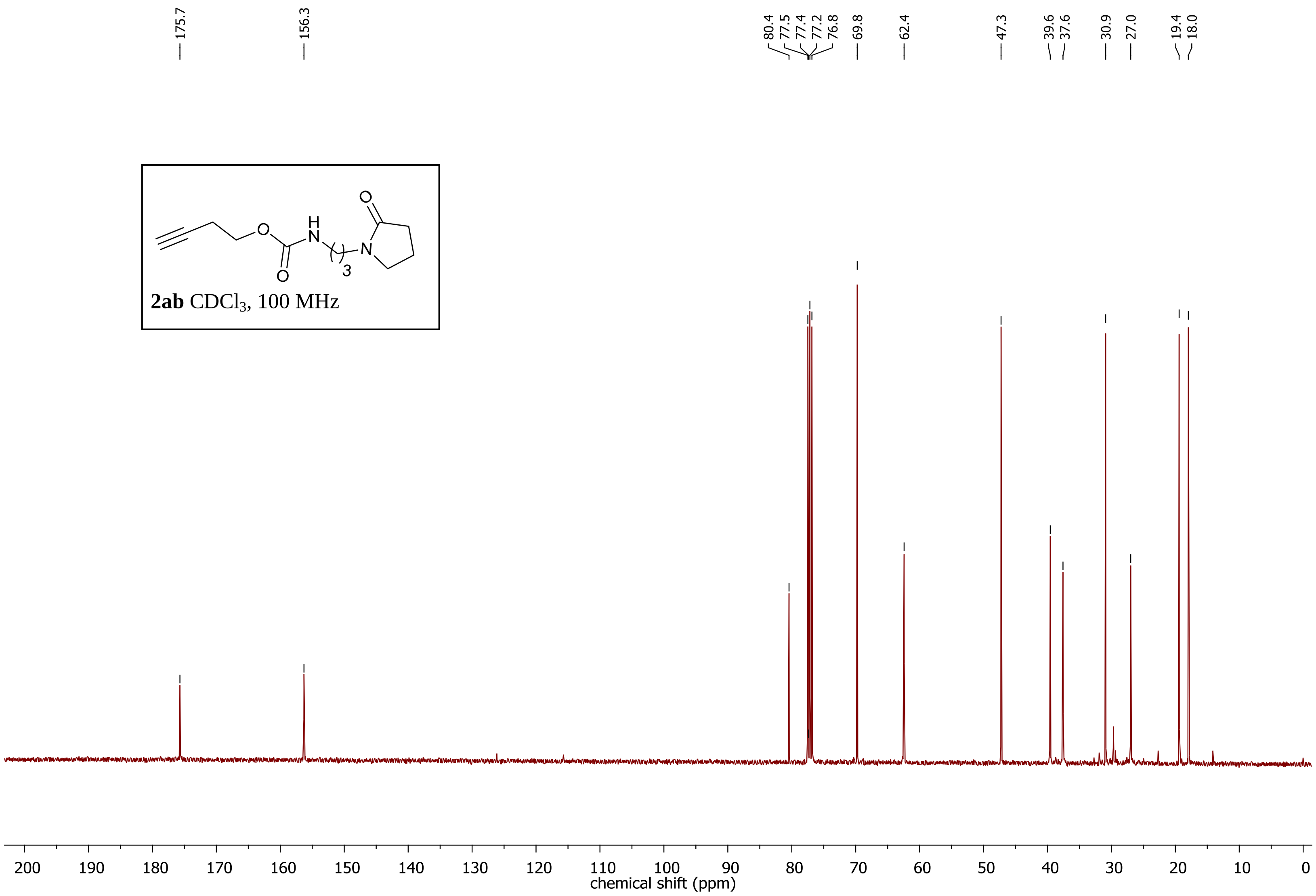
175.7
156.3
80.4
77.5
77.4
77.2
76.8
69.8
62.4
47.3
39.6
37.6
30.9
27.0
19.4
18.0
2ab CDCl3, 100 MHz
200 190 180 170 160 150 140 130 120 110 100 90 80 70 60 50 40 30 20 10 0
chemical shift (ppm)

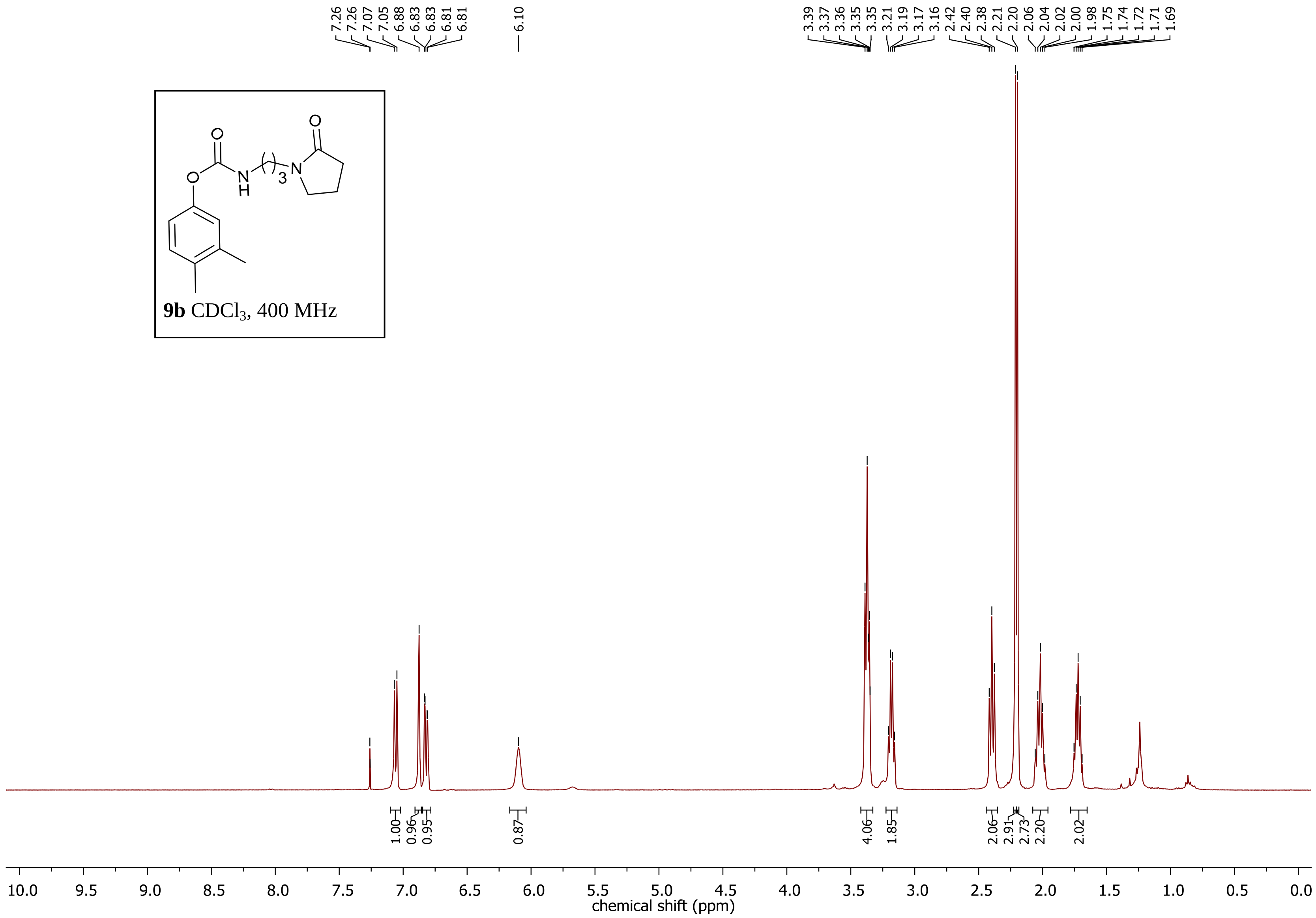
7.26
7.26
7.07
7.05
6.88
6.83
6.83
6.81
6.81
6.10
3.39
3.37
3.36
3.35
3.35
3.21
3.19
3.17
3.16
2.42
2.40
2.38
2.21
2.20
2.06
2.04
2.02
2.00
1.98
1.75
1.74
1.72
1.71
1.69
9b CDCl3, 400 MHz
1.00
0.96
0.95
0.87
4.06
1.85
2.06
2.91
2.73
2.20
2.02
10.0
9.5
9.0
8.5
8.0
7.5
7.0
6.5
6.0
5.5
5.0
4.5
4.0
3.5
3.0
2.5
2.0
1.5
1.0
0.5
0.0
chemical shift (ppm)

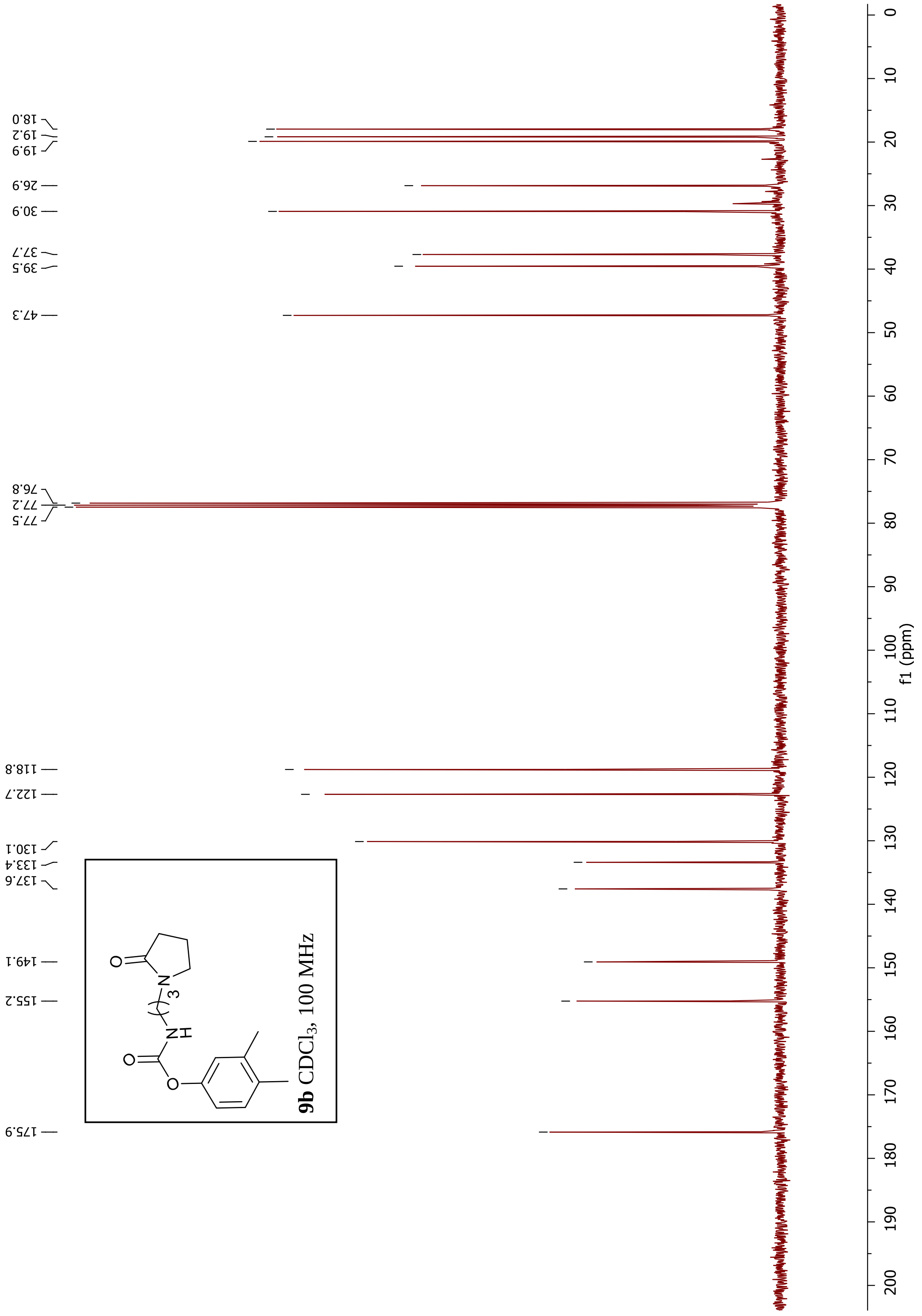
18.0
19.2
19.9
26.9
30.9
37.7
39.5
47.3
76.8
77.2
77.5
118.8
122.7
130.1
133.4
137.6
149.1
155.2
175.9
f1 (ppm)
9b $CDCl_3$, 100 MHz

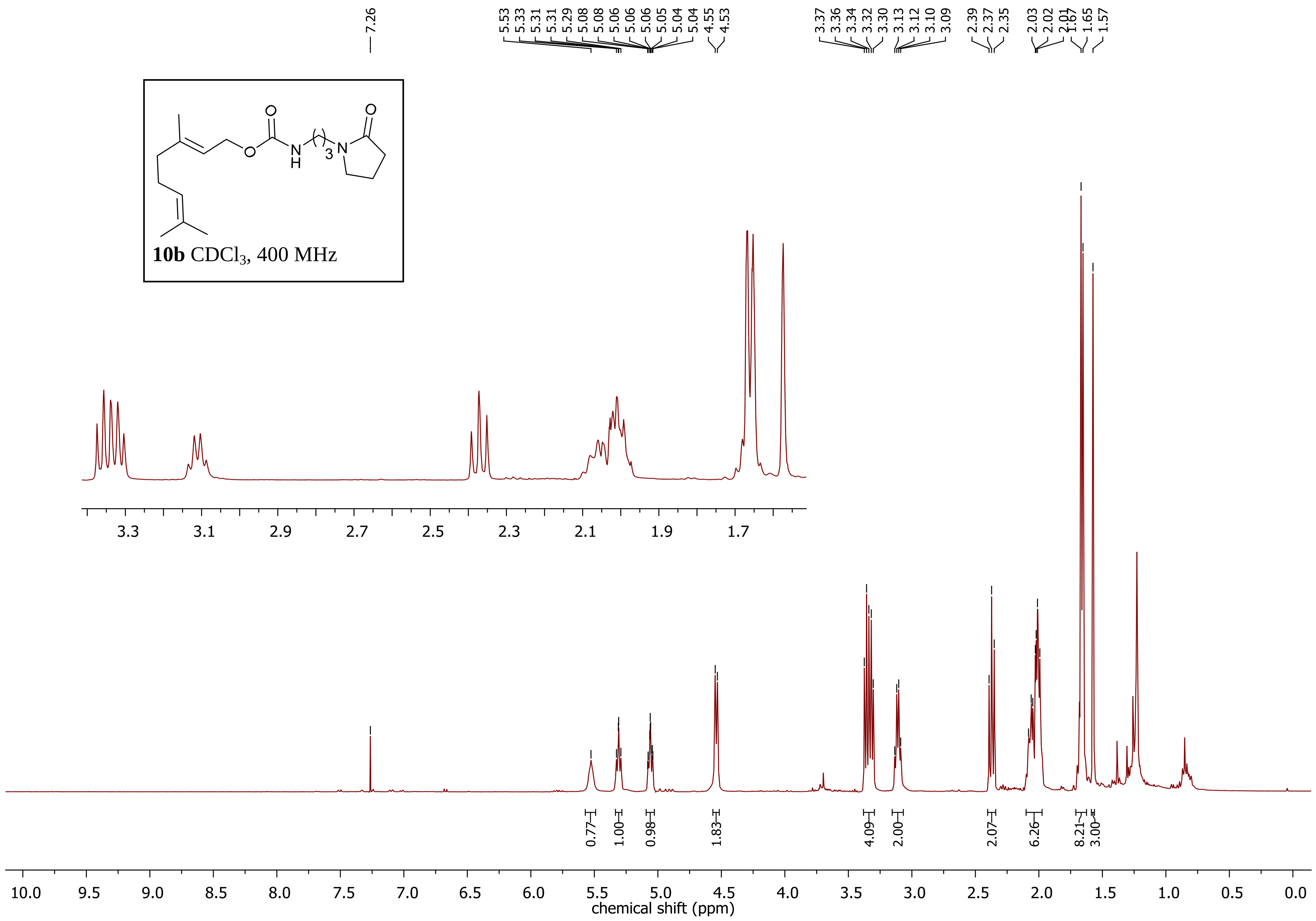
10b CDCl$_3$, 400 MHz
7.26
5.53 5.33 5.31 5.31 5.29 5.08 5.08 5.06 5.06 5.06 5.05 5.04 5.04 4.55 4.53
3.37 3.36 3.34 3.32 3.30 3.13 3.12 3.10 3.09
2.39 2.37 2.35 2.03 2.02 2.01 1.67 1.65 1.57
0.77 1.00 0.98 1.83 4.09 2.00 2.07 6.26 8.21 3.00
3.3 3.1 2.9 2.7 2.5 2.3 2.1 1.9 1.7
10.0 9.5 9.0 8.5 8.0 7.5 7.0 6.5 6.0 5.5 5.0 4.5 4.0 3.5 3.0 2.5 2.0 1.5 1.0 0.5 0.0
chemical shift (ppm)

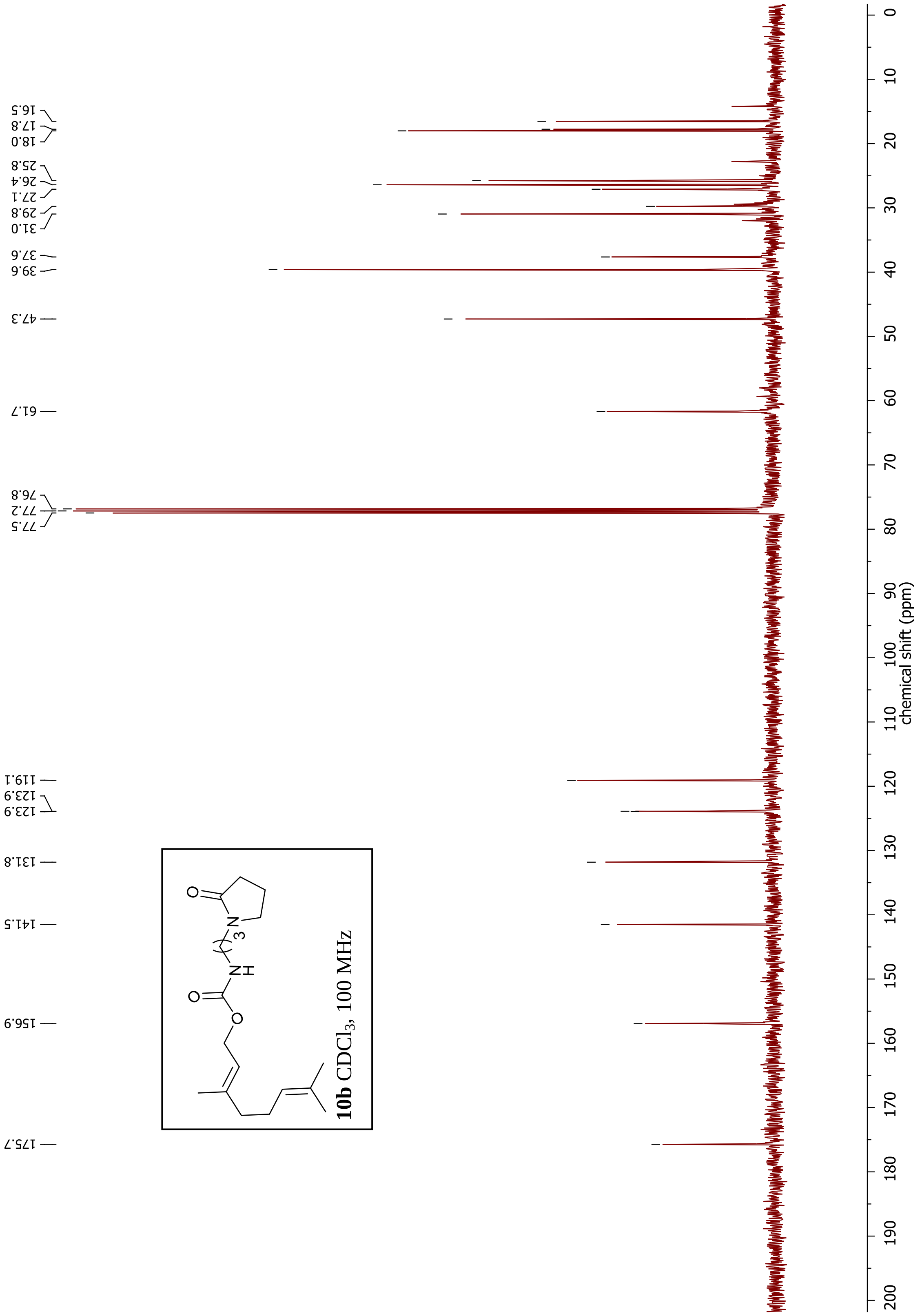
16.5
17.8
18.0
25.8
26.4
27.1
29.8
31.0
37.6
39.6
47.3
61.7
76.8
77.2
77.5
119.1
123.9
123.9
131.8
141.5
156.9
175.7
0
10
20
30
40
50
60
70
80
90
100
110
120
130
140
150
160
170
180
190
200
chemical shift (ppm)
10b CDCl3, 100 MHz

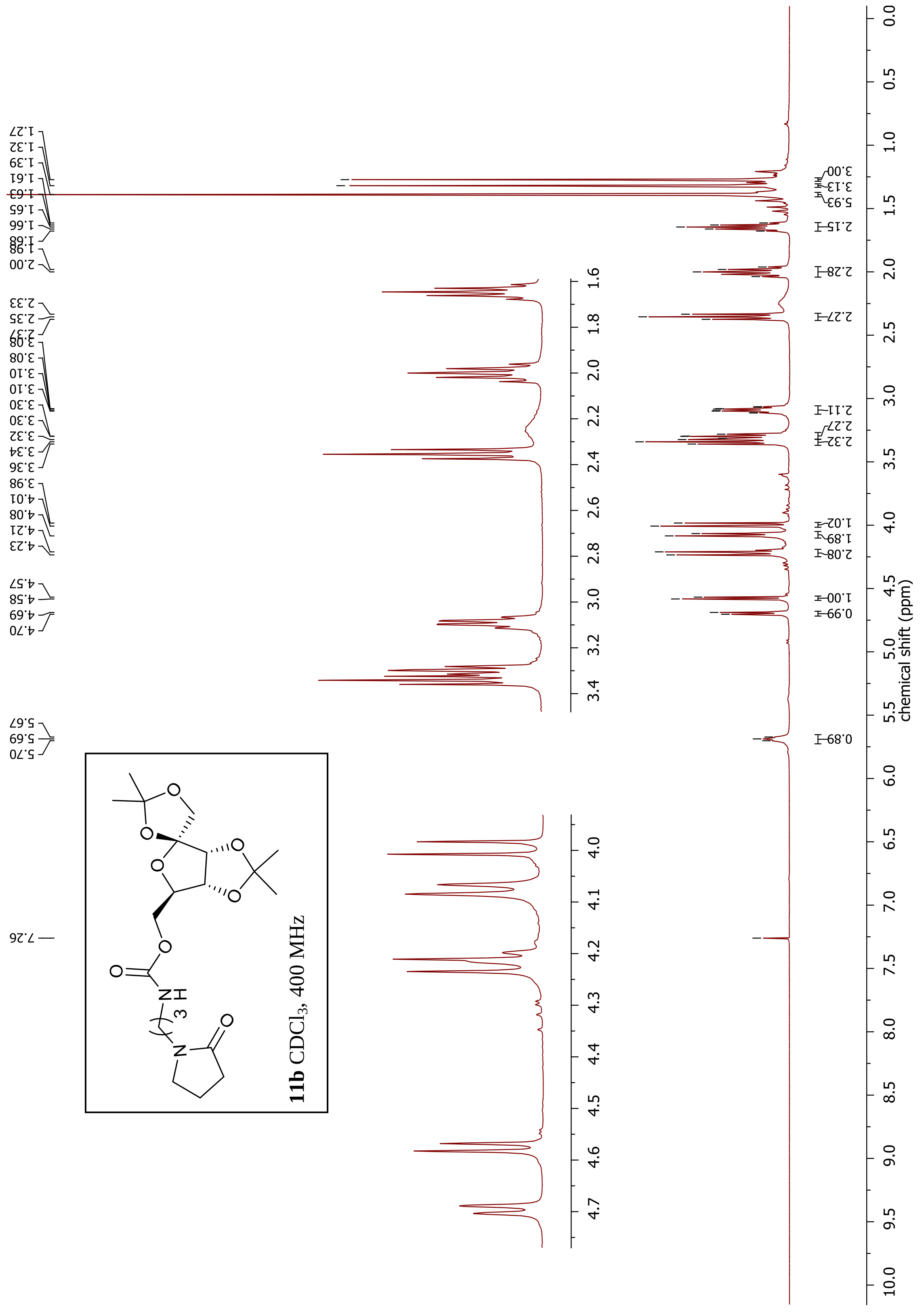
11b CDCl3, 400 MHz
chemical shift (ppm)

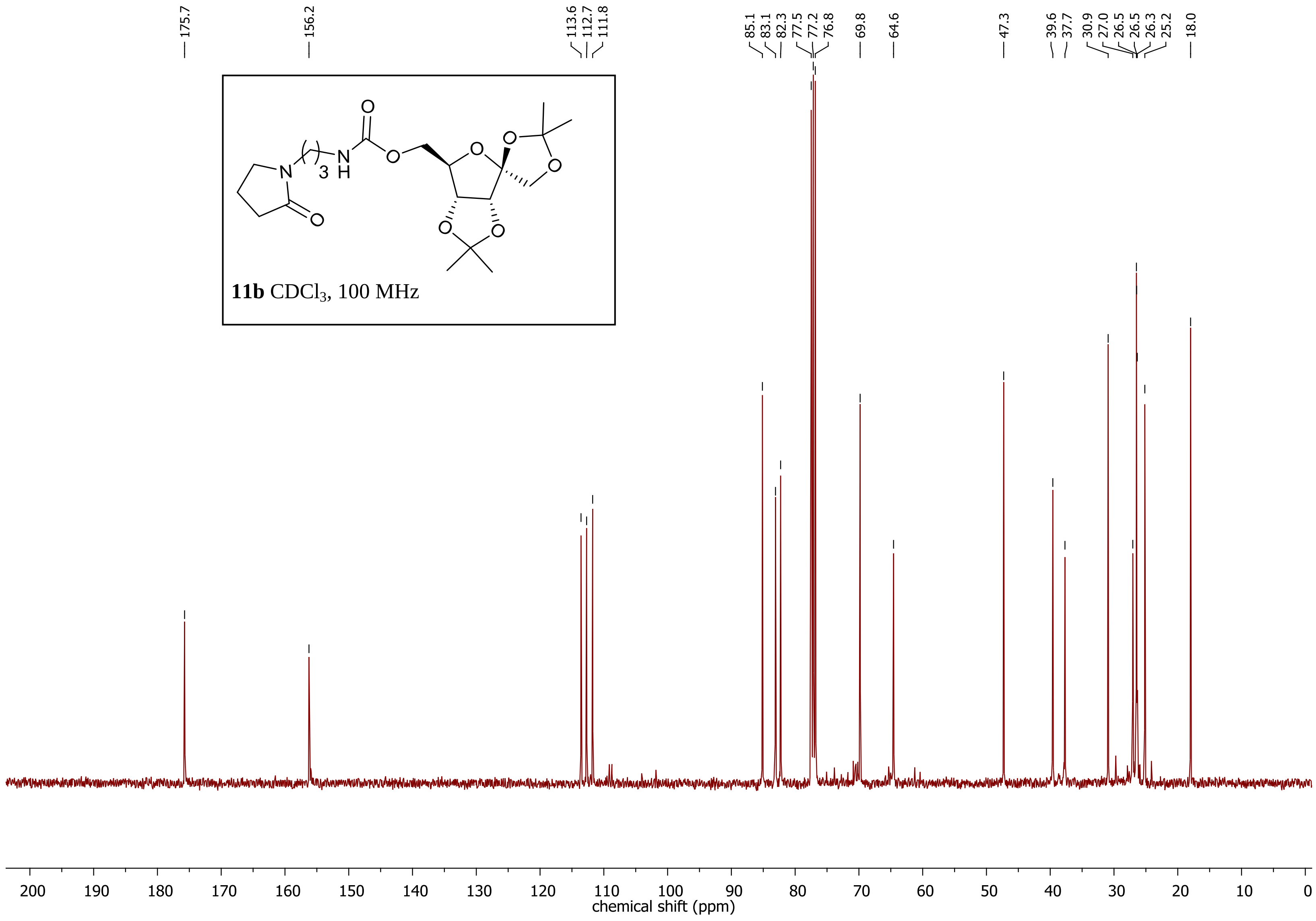

11b $CDCl_3$, 100 MHz

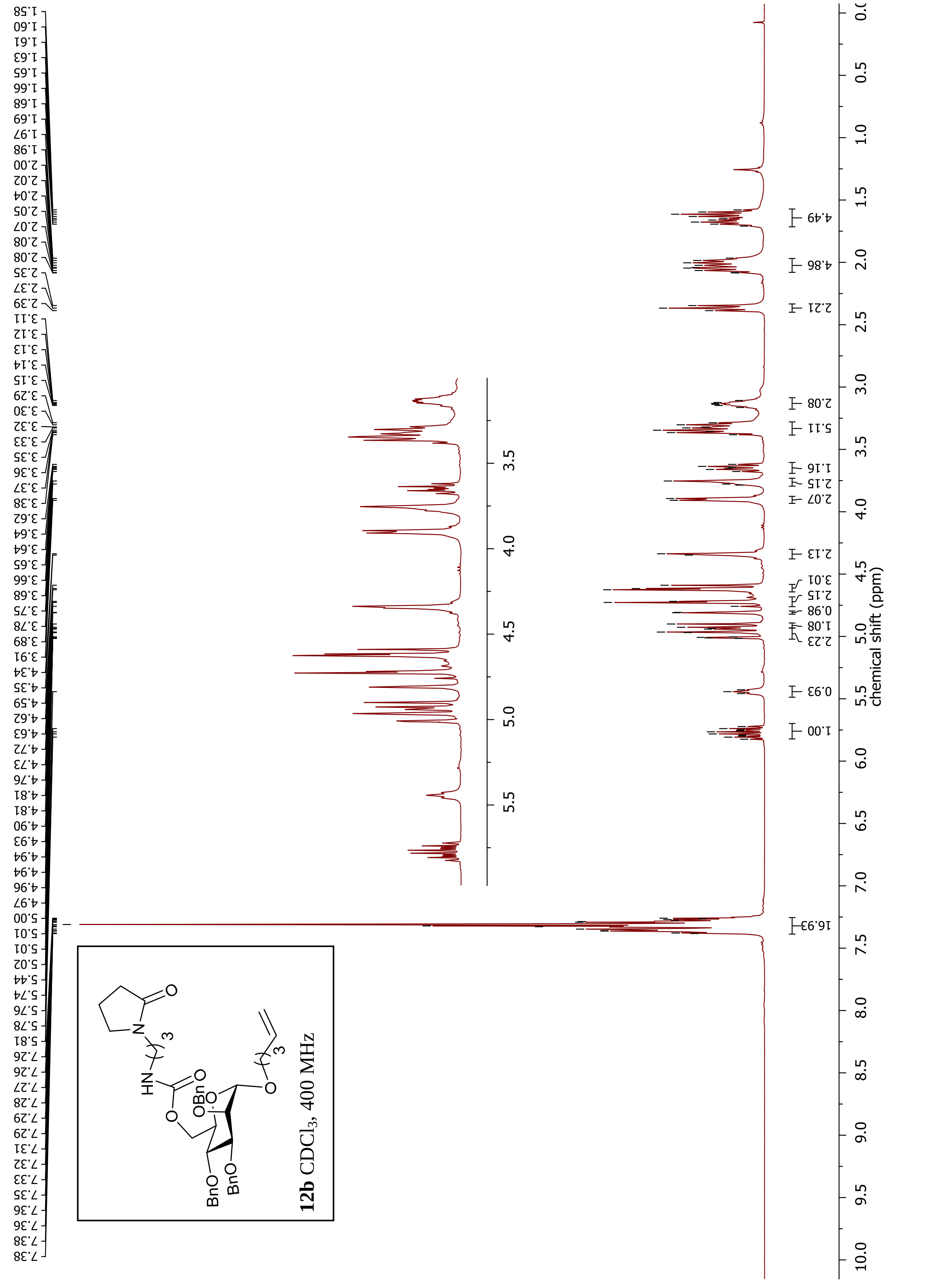
12b CDCl3, 400 MHz
chemical shift (ppm)

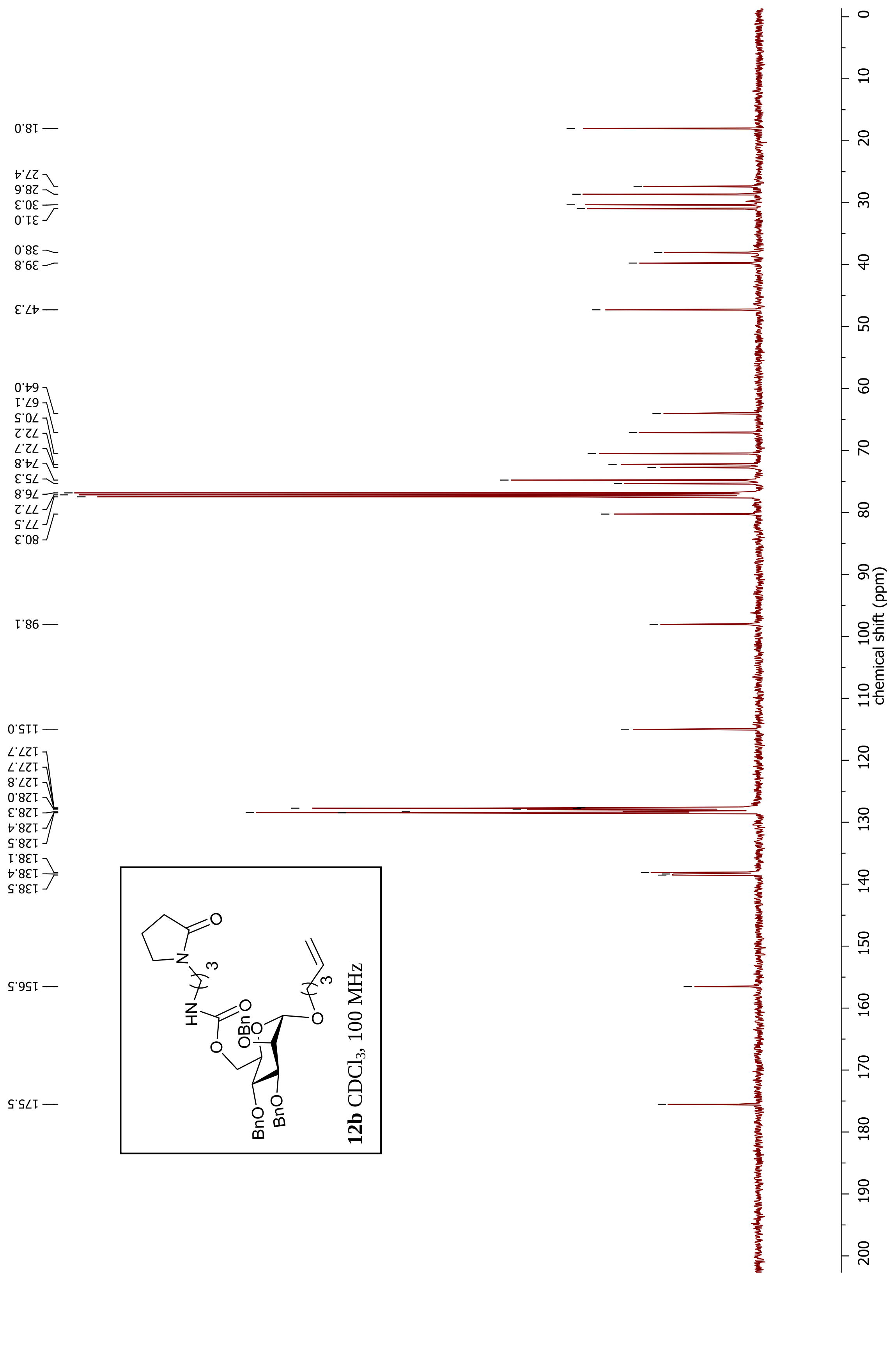
18.0
27.4
28.6
30.3
31.0
38.0
39.8
47.3
64.0
67.1
70.5
72.2
72.7
74.8
75.3
76.8
77.2
77.5
80.3
98.1
115.0
127.7
127.7
127.8
128.0
128.3
128.4
128.5
138.1
138.4
138.5
156.5
175.5
chemical shift (ppm)
12b CDCl3, 100 MHz

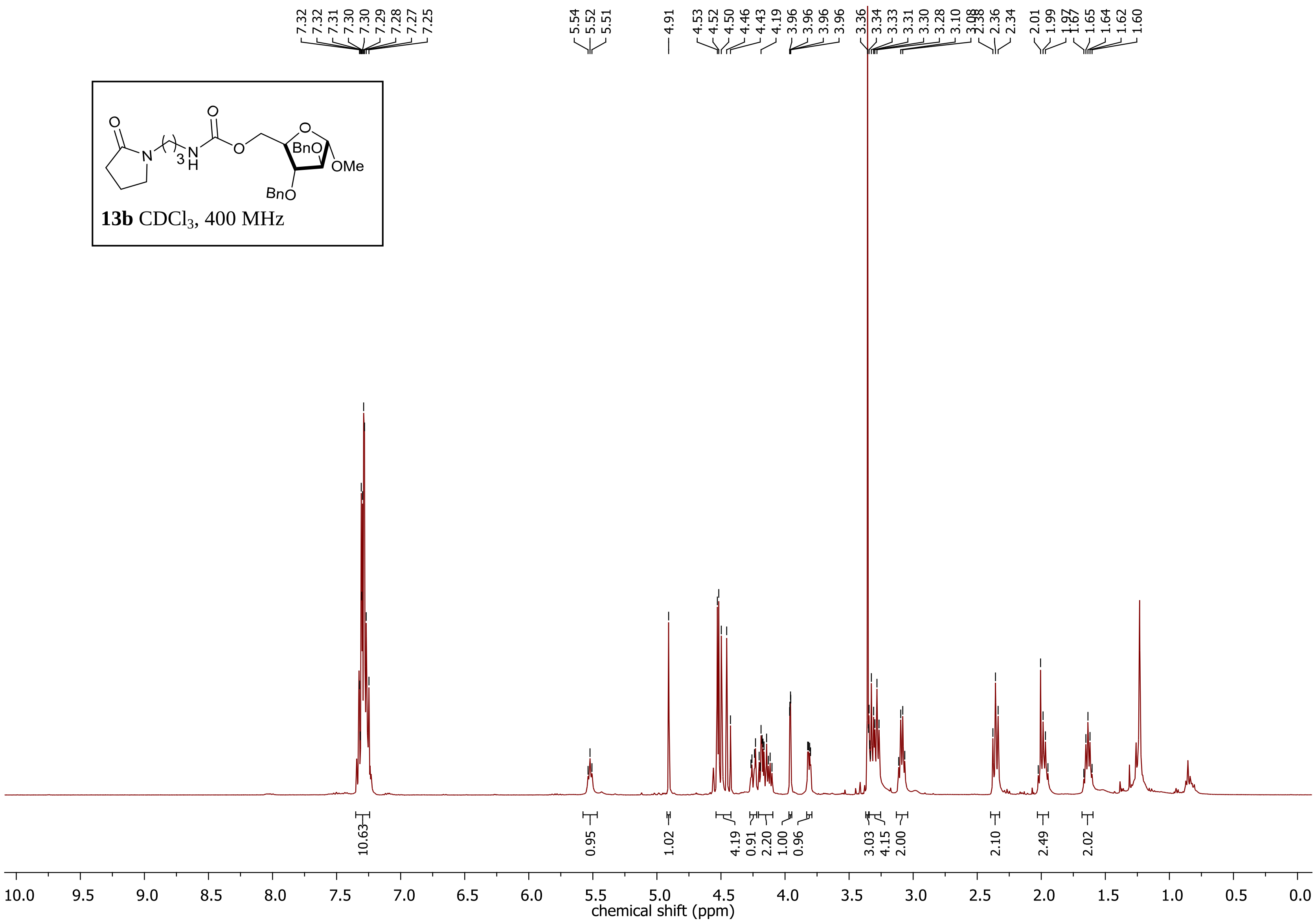

13b $CDCl_3$, 400 MHz

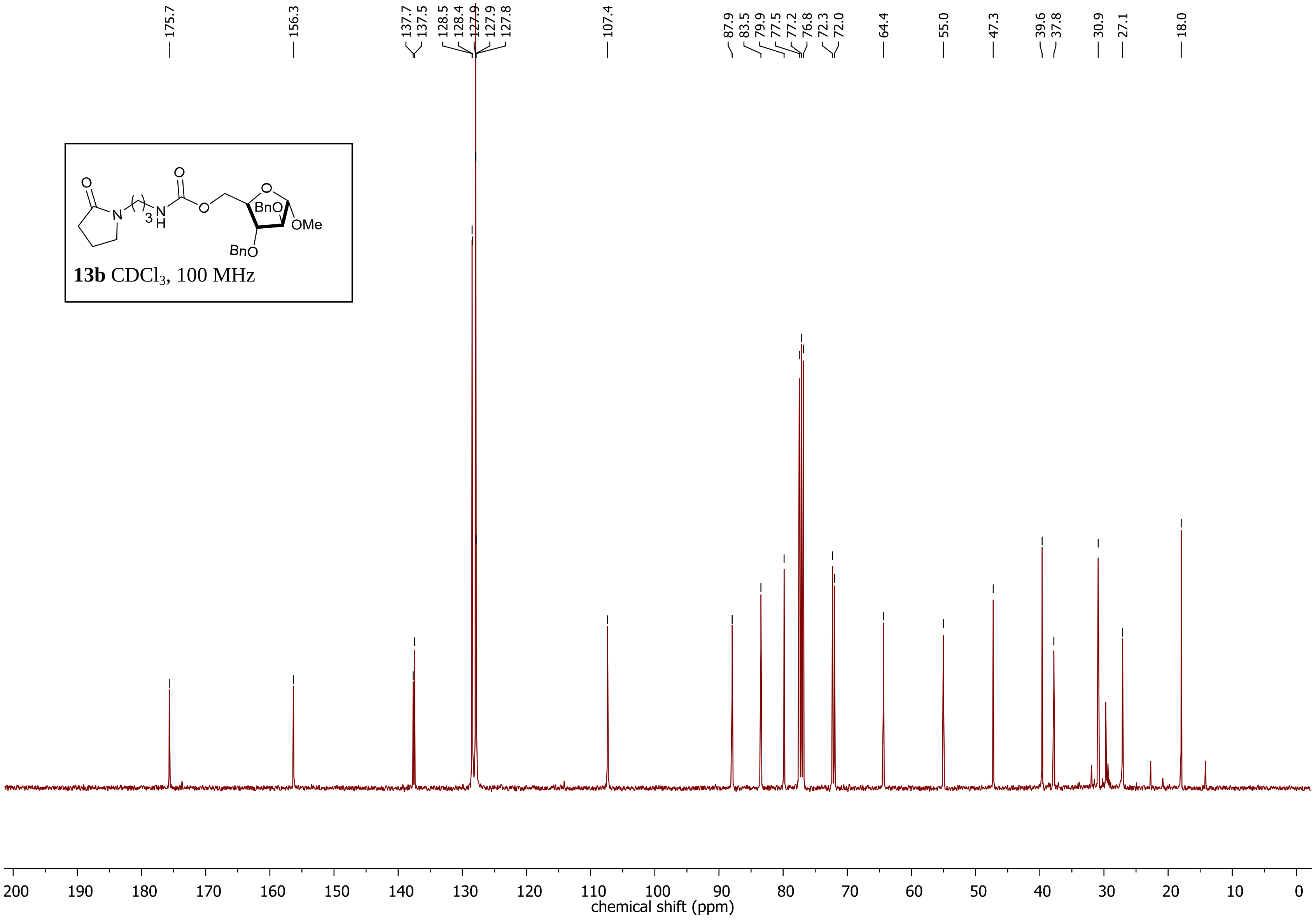
175.7
156.3
137.7
137.5
128.5
128.4
127.9
127.9
127.8
107.4
87.9
83.5
79.9
77.5
77.2
76.8
72.3
72.0
64.4
55.0
47.3
39.6
37.8
30.9
27.1
18.0
O
N
3
H
O
O
BnO
OMe
BnO
13b CDCl3, 100 MHz
200 190 180 170 160 150 140 130 120 110 100 90 80 70 60 50 40 30 20 10 0
chemical shift (ppm)

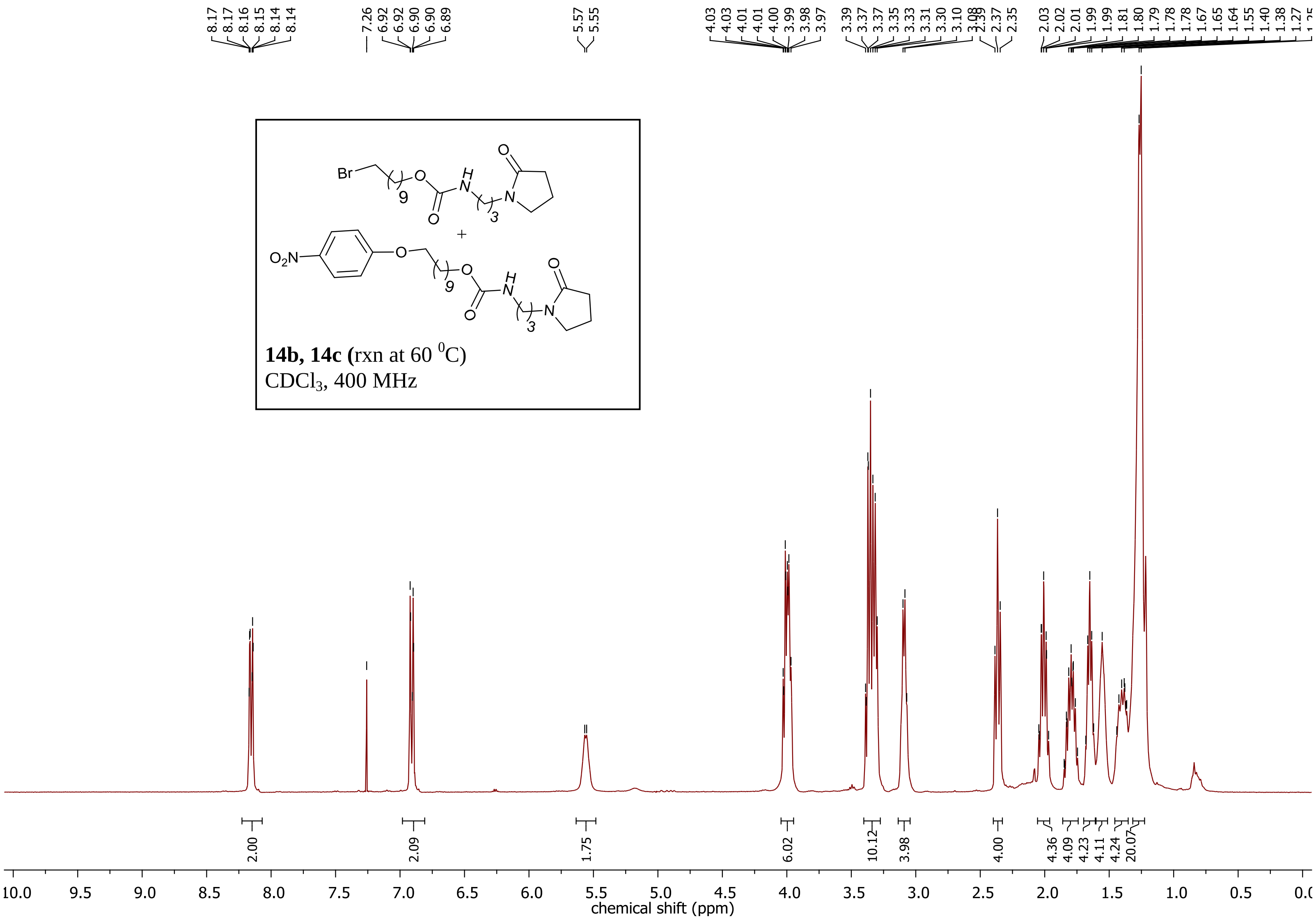
Br
O
H
N
O
N
O
9
3
+
O2N
O
O
H
N
O
N
O
9
3
14b, 14c (rxn at 60 0C)
CDCl3, 400 MHz
8.17 8.17 8.16 8.15 8.14 8.14
7.26
6.92 6.92 6.90 6.90 6.89
5.57 5.55
4.03 4.03 4.01 4.01 4.00 3.99 3.98 3.97
3.39 3.37 3.37 3.35 3.33 3.31 3.30 3.10 3.08
2.39 2.37 2.35
2.03 2.02 2.01 1.99 1.99 1.81 1.80 1.79 1.78 1.78 1.67 1.65 1.64 1.55 1.40 1.38 1.27
2.00
2.09
1.75
6.02
10.12
3.98
4.00
4.36
4.09
4.23
4.11
4.24
20.07
10.0 9.5 9.0 8.5 8.0 7.5 7.0 6.5 6.0 5.5 5.0 4.5 4.0 3.5 3.0 2.5 2.0 1.5 1.0 0.5
chemical shift (ppm)

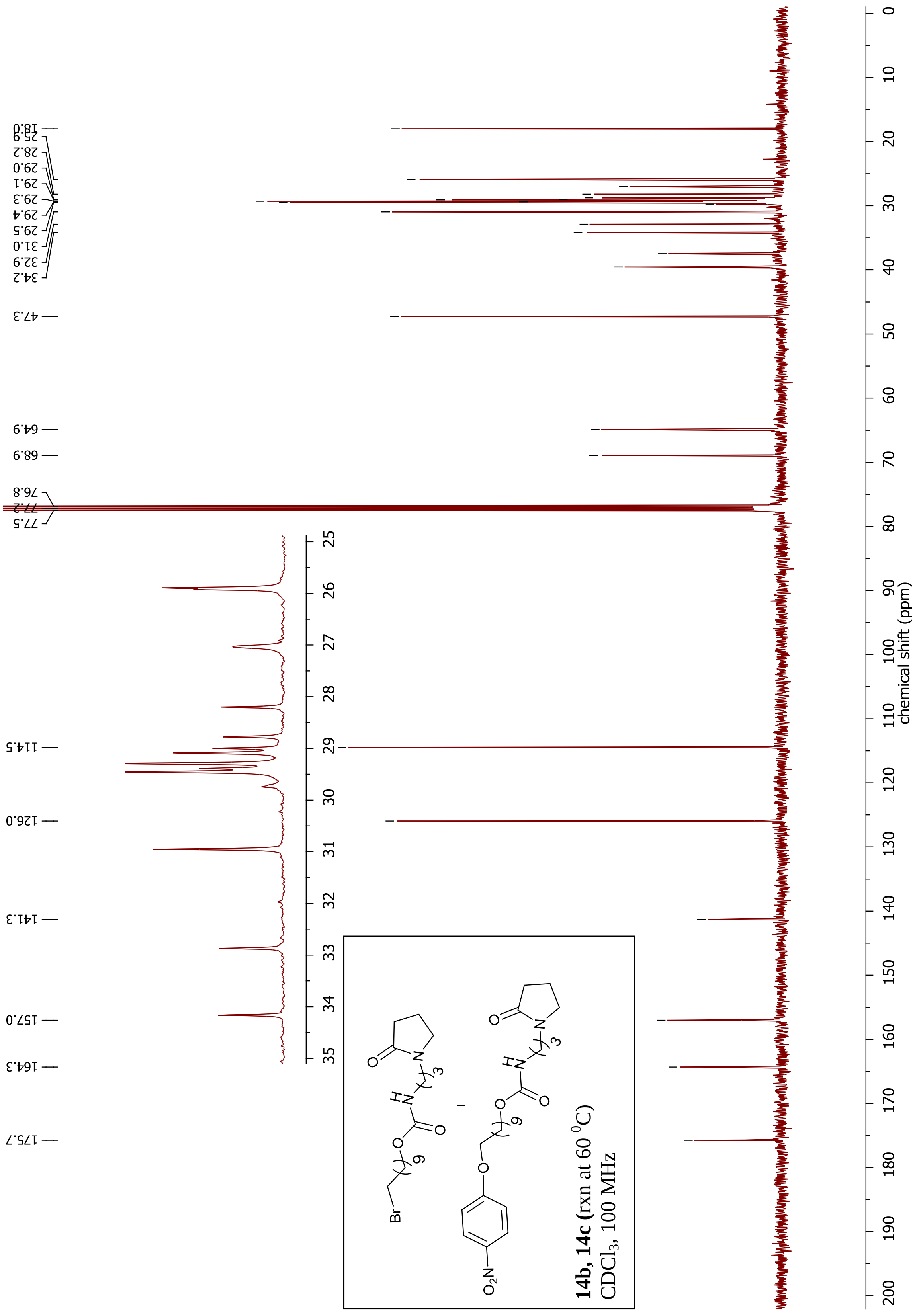

18.0
25.9
28.2
29.0
29.1
29.3
29.4
29.5
31.0
32.9
34.2
47.3
64.9
68.9
76.8
77.2
77.5
114.5
126.0
141.3
157.0
164.3
175.7
chemical shift (ppm)
14b, 14c (rxn at 60 °C)
CDCl3, 100 MHz

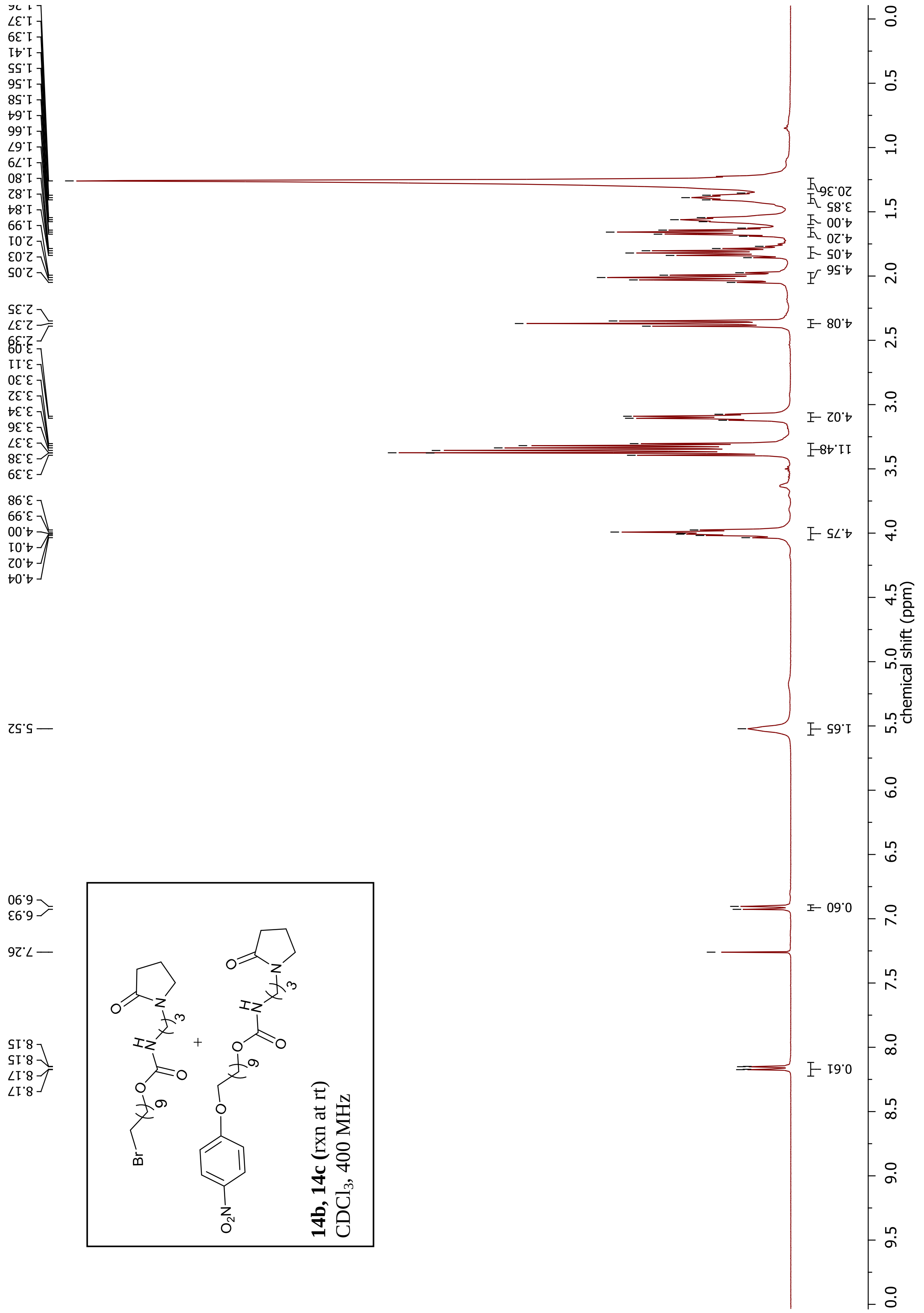
14b, 14c (rxn at rt)
$CDCl_3$, 400 MHz
chemical shift (ppm)
8.17 8.17 8.15 8.15
7.26
6.93 6.90
5.52
4.04 4.02 4.01 4.00 3.99 3.98
3.39 3.38 3.37 3.36 3.34 3.32 3.30 3.11 3.09
2.39 2.37 2.35
2.05 2.03 2.01 1.99 1.84 1.82 1.80 1.79 1.67 1.66 1.64 1.58 1.56 1.55 1.41 1.39 1.37 1.36
0.61 0.60 1.65 4.75 11.48 4.02 4.08 4.56 4.05 4.20 4.00 3.85 20.36

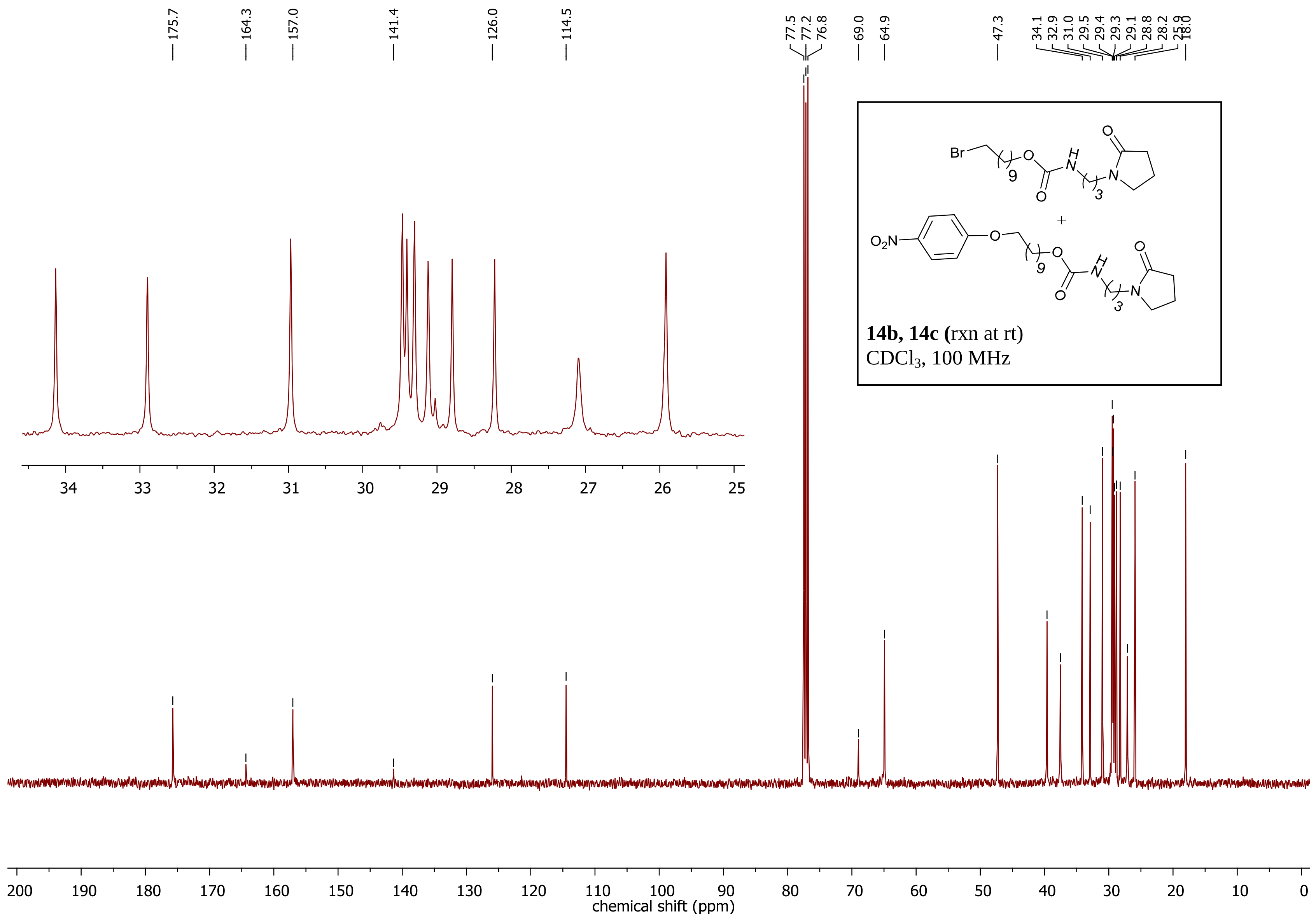
175.7
164.3
157.0
141.4
126.0
114.5
77.5
77.2
76.8
69.0
64.9
47.3
34.1
32.9
31.0
29.5
29.4
29.3
29.1
28.8
28.2
25.9
18.0
Br
O
H
N
9
3
+
O_2N
14b, 14c (rxn at rt)
$CDCl_3$, 100 MHz
chemical shift (ppm)

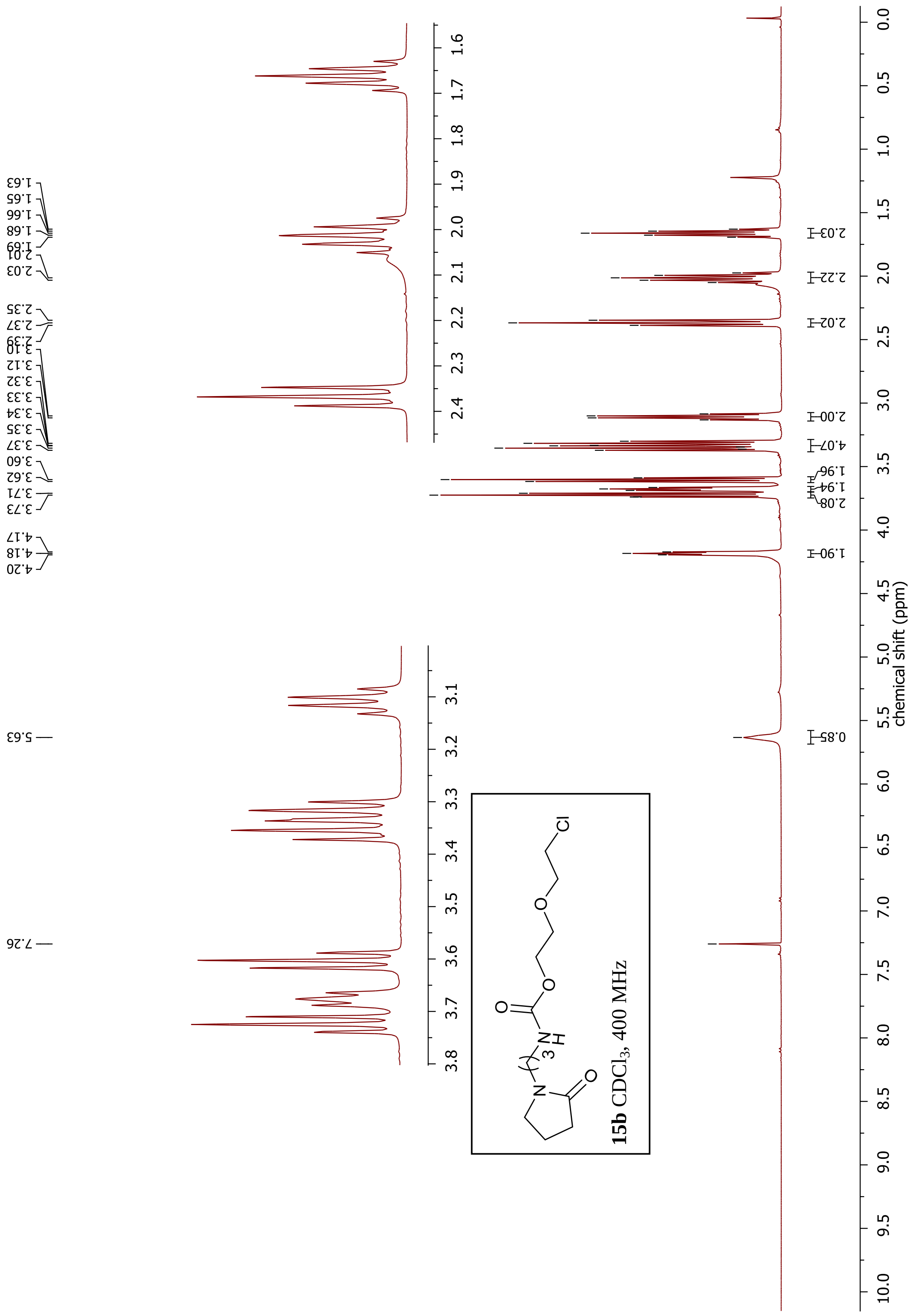

15b CDCl3, 400 MHz
chemical shift (ppm)

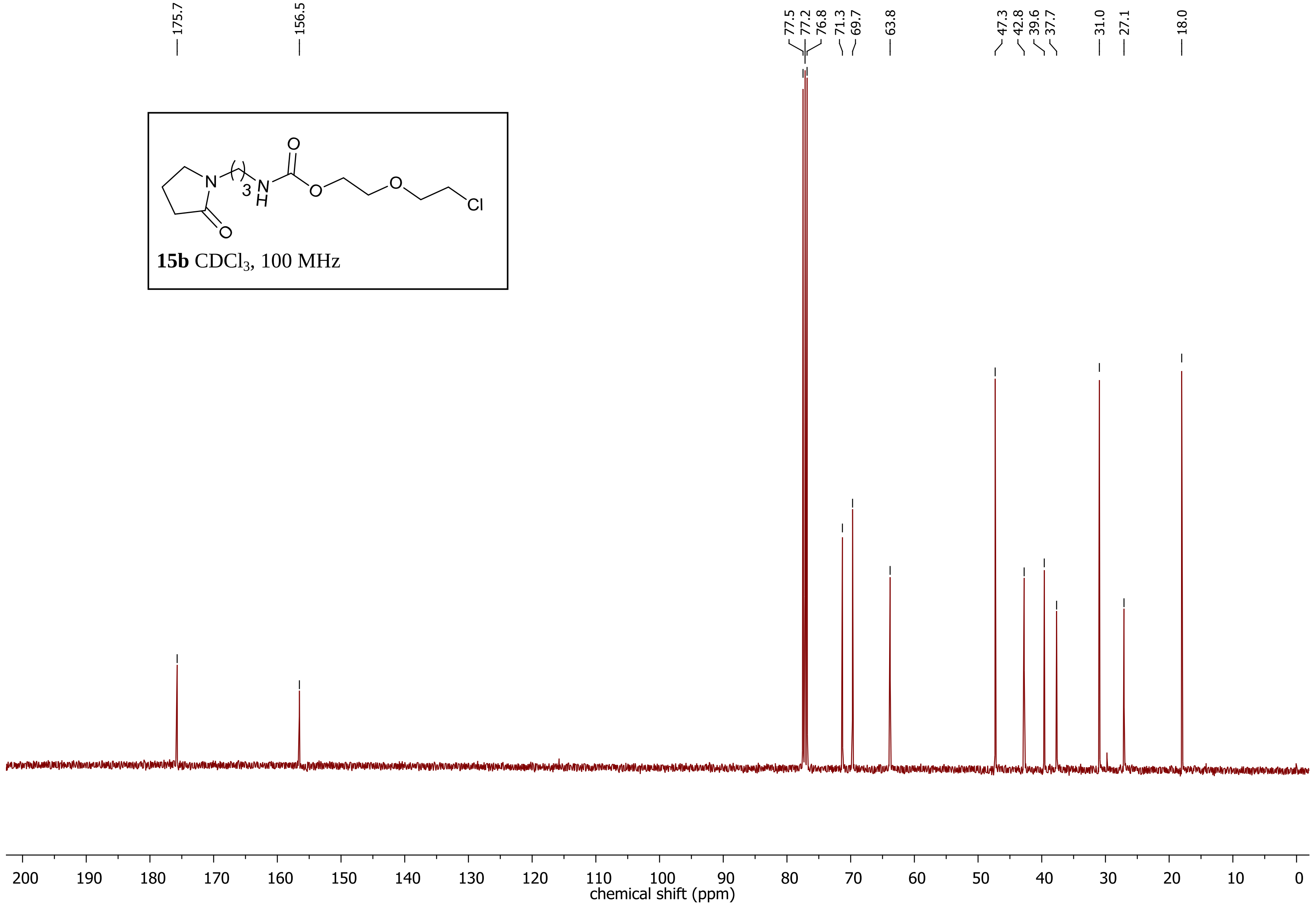

15b $CDCl_3$, 100 MHz
